# Supplementation of Saussurea costus root alleviates sodium nitrite-induced hepatorenal toxicity by modulating metabolic profile, inflammation, and apoptosis

**DOI:** 10.3389/fphar.2024.1378249

**Published:** 2024-05-30

**Authors:** Samy E. Elshaer, Gamal M. Hamad, Sherien E. Sobhy, Amira M. Galal Darwish, Hoda H. Baghdadi, Hebatallah H. Abo Nahas, Fatma M. El-Demerdash, Sanaa S. A. Kabeil, Abdulmalik S. Altamimi, Ebtesam Al-Olayan, Maha Alsunbul, Omaima Kamel Docmac, Mariusz Jaremko, Elsayed E. Hafez, Essa M. Saied

**Affiliations:** ^1^ Department of Environmental Studies, Institute of Graduate Studies and Research, Alexandria University, Alexandria, Egypt; ^2^ Department of Food Technology, Arid Lands Cultivation Research Institute (ALCRI), City of Scientific Research and Technological Applications (SRTA-City), Alexandria, Egypt; ^3^ Department of Plant Protection and Biomolecular Diagnosis, Arid Lands Cultivation Research Institute (ALCRI), City of Scientific Research and Technological Applications (SRTA-City), Alexandria, Egypt; ^4^ Food Industry Technology Program, Faculty of Industrial and Energy Technology, Borg Al Arab Technological University (BATU), Alexandria, Egypt; ^5^ Zoology Department, Faculty of Science, Port Said University, Port Said, Egypt; ^6^ Department of Protein Research, Genetic Engineering and Biotechnology Research Institute (GEBRI), City of Scientific Research and Technological Applications (SRTA-City), Alexandria, Egypt; ^7^ Department of Pharmaceutical Chemistry, College of Pharmacy, Prince Sattam Bin Abdulaziz University, Alkharj, Saudi Arabia; ^8^ Department of Zoology, College of Science, King Saud University, Riyadh, Saudi Arabia; ^9^ Department of Pharmaceutical Sciences., College of Pharmacy, Princess Nourah bint Abdulrahman University, Riyadh, Saudi Arabia; ^10^ Anatomy and Embryology Department, Faculty of Medicine, Tanta University, Tanta, Egypt; ^11^ Smart-Health Initiative and Red Sea Research Center, Division of Biological and Environmental Sciences and Engineering, King Abdullah University of Science and Technology, Thuwal, Saudi Arabia; ^12^ Chemistry Department (Biochemistry Division), Faculty of Science, Suez Canal University, Ismailia, Egypt; ^13^ Institute for Chemistry, Humboldt Universität zu Berlin, Berlin, Germany

**Keywords:** food additives, sodium nitrite, Saussurea costus, phytochemical profile, hepatorenal protective, metabolic analysis, histopathology analysis, immunohistochemistry analysis

## Abstract

Sodium nitrite (NaNO_2_) is a widely used food ingredient, although excessive concentrations can pose potential health risks. In the present study, we evaluated the deterioration effects of NaNO_2_ additives on hematology, metabolic profile, liver function, and kidney function of male Wistar rats. We further explored the therapeutic potential of supplementation with *S. costus* root ethanolic extract (SCREE) to improve NaNO_2_-induced hepatorenal toxicity. In this regard, 65 adult male rats were divided into eight groups; Group 1: control, Groups 2, 3, and 4 received SCREE in 200, 400, and 600 mg/kg body weight, respectively, Group 5: NaNO_2_ (6.5 mg/kg body weight), Groups 6, 7 and 8 received NaNO_2_ (6.5 mg/kg body weight) in combination with SCREE (200, 400, and 600 mg/kg body weight), respectively. Our results revealed that the NaNO_2_-treated group shows a significant change in deterioration in body and organ weights, hematological parameters, lipid profile, and hepatorenal dysfunction, as well as immunohistochemical and histopathological alterations. Furthermore, the NaNO_2_-treated group demonstrated a considerable increase in the expression of TNF-α cytokine and tumor suppressor gene P53 in the kidney and liver, while a significant reduction was detected in the anti-inflammatory cytokine IL-4 and the apoptosis suppressor gene BCL-2, compared to the control group. Interestingly, SCREE administration demonstrated the ability to significantly alleviate the toxic effects of NaNO_2_ and improve liver function in a dose-dependent manner, including hematological parameters, lipid profile, and modulation of histopathological architecture. Additionally, SCREE exhibited the ability to modulate the expression levels of inflammatory cytokines and apoptotic genes in the liver and kidney. The phytochemical analysis revealed a wide set of primary metabolites in SCREE, including phenolics, flavonoids, vitamins, alkaloids, saponins and tannins, while the untargeted UPLC/T-TOF–MS/MS analysis identified 183 metabolites in both positive and negative ionization modes. Together, our findings establish the potential of SCREE in mitigating the toxic effects of NaNO_2_ by modulating metabolic, inflammatory, and apoptosis. Together, this study underscores the promise of SCREE as a potential natural food detoxifying additive to counteract the harmful impacts of sodium nitrite.

## 1 Introduction

Food additives are natural or synthetic substances that are incorporated into food products to preserve or enhance their flavor, appearance, and taste (Yılmaz et al., 2009; Bari and Yeasmin, 2018). There are several types of additives, including emulsifiers, stabilizers, preservatives, and coloring agents (Wu et al., 2022). Sodium nitrite is a key component in food additives, commonly used in meat, fish, and certain cheeses for coloring, preservation, and antibacterial agent, and also imparts flavor and color to meat (Osman et al., 2021; Cvetković et al., 2019). Its inhibitory effect on the synthesis of iron-sulfur complexes, particularly notable in frozen meats, effectively suppresses the development of *clostridium botulinum* spores (Milkowski et al., 2010). The vibrant color in meat is the result of the reaction of sodium nitrite with myoglobin that leads to the formation of nitrosyl myoglobin (Sindelar and Milkowski, 2012). Moreover, sodium nitrite effectively delays the onset of oxidative rancidity by binding to heme proteins and metal ions and neutralizing free radicals (Sullivan et al., 2012). However, sodium nitrite can also react with amines and amides in gastric juices, leading to the formation of nitrosamines, potent carcinogens. In addition, this reaction results in the production of free radicals, further complicating the potential health implications of sodium nitrite consumption (Hassan and Ali, 2010; Hassan and Yousef, 2010). Nitrite is also a potent generator of nitric oxide with harmful biological effects (Jensen, 2007). Prolonged consumption of sodium nitrite in food has the potential to induce tissue damage, cardiac toxicity, hepatotoxicity, nephrotoxicity, inflammation, fibrosis, and apoptosis (Hassan et al., 2009; Hassan et al., 2010; El-Sheikh and Khalil, 2011; Salama et al., 2013; Al-Gayyar et al., 2015; Fadda et al., 2018a). Consequently, the rise of considerable environmental and health concerns underscores the importance of intensifying efforts towards the innovation of new and safe food additives and preservatives. These innovations aim to mitigate the deteriorative impact associated with the use of sodium nitrite additives.

Phytochemical substances or secondary metabolites continue to be considered the most promising options. Recent research has placed increasing emphasis on the importance of secondary metabolites in plants, given their numerous biological and medicinal applications. Natural products continue to be a significant source of innovation in drug discovery (Abdallah et al., 2017). Dolomiaea costus (Falc.) Kasana and A.K. Pandey [Asteraceae], a member of the Asteraceae family commonly known as Saussurea costus (Falc.) Lipsch. and Saussurea lappa (Decne.) Sch. Bip is a highly prevalent botanical species employed for several therapeutic purposes. The primary metabolites of Saussurea costus (S. costus) are sesquiterpene lactones, specifically costunolide, dehydrocostus lactone, and cynaropicrin (Abd El-Rahman et al., 2020). The Saussurea genus encompasses many species that are known to contain sesquiterpene lactones, triterpenes, steroids, lignans, and flavonoids. It is worth noting that certain metabolites within this group have intriguing biological activity (Julianti, 2014; Attallah et al., 2023). The essential oil of the roots of S. costus is mainly composed of sesquiterpenoids, which account for approximately 79.80% of its total composition (Hanh et al., 2021). Costunolide, a sesquiterpene lactone that is commonly obtained from the roots of S. costus, exhibits a wide spectrum of biological activity, including antioxidant, anti-inflammatory, neuroprotective, and antidiabetic effects (Moujir et al., 2020). The roots of S. costus were shown to contain acetylated flavone glycosides, palmitic and linoleic acids, as well as chlorogenic acid (El Gizawy et al., 2022). Recent studies have demonstrated that these metabolites exhibit various biological properties, including antifungal (Barrero et al., 2000), antidiabetic, anticancer, and antiprotozoal (Ko et al., 2005), immunostimulant (Kulkarni, 2001), antiulcer (Sutar et al., 2011), antimicrobial (Khalid et al., 2011), anti-inflammatory (Sunkara et al., 2010) and anti-hepatotoxic (Yaeesh et al., 2010) activities. In a recent study conducted by Deabes et al. (2021), it was shown that extracts derived from S. costus have notable efficacy in combating multiantibiotic-resistant human infections. These findings suggest that these extracts could serve as a viable alternative to antibiotics for the treatment of certain infections. Elshaer et al. (2022) investigated the impact of three distinct extracts derived from roots of S. costus, namely, ethanol, methanol, and water when used as a food additive.

To comprehensively analyze the diverse chemical classes and properties, as well as the wide range of metabolite concentrations in plants, it is necessary to utilize a diverse range of analytical techniques in plant metabolomics. Liquid chromatography tandem mass spectrometry (LC-MS/MS) is a robust analytical technique widely used for the identification and characterization of plant metabolites (Lu et al., 2008). Various LC-MS-based metabolomics platforms have been developed so far for the targeted analysis of primary metabolites (Sawada et al., 2009), photosynthetic intermediates (Arrivault et al., 2009), lipids and fatty acids (Okazaki et al., 2013; Bromke et al., 2015), phytohormones (Seo et al., 2011), secondary metabolites (Tohge and Fernie, 2010), and untargeted metabolome analysis (Shahaf et al., 2016). Analytical approaches are based on untargeted mass spectrometry. The application of advanced liquid chromatography tandem mass spectrometry (LC-MS/MS), distinguished by its exceptional resolution, has enabled thorough exploration of the metabolome in diverse biological samples, including those from herbal medicine. Through the analysis of mass fragmentation (MS/MS) spectra, valuable information on the structural characteristics of metabolites can be inferred from their unique spectral patterns (Beniddir et al., 2021).

Given the wide range of biological activity exhibited by the *S. costus* extract and our ongoing efforts to discover new bioactive molecules with pharmacological potential (Samaha et al., 2020; Khirallah et al., 2022; Salem et al., 2022; Mohamed et al., 2022a; Mohamed et al., 2022b), in this study, we aimed to gain insight into the hepatorenal deterioration effect of sodium nitrite additive in an animal model and to explore the possible protective activity of 70% SCREE to ameliorate these effects. In this regard, we performed several analytical techniques to characterize phytochemical metabolites in SCREE employing several chemical and analytical techniques (HPLC and UPLC/T-TOF–MS/MS-based analysis). Furthermore, we unveiled the possible hepatorenal protective potency of SCREE against NaNO2-induced liver and kidney toxicity by performing multiinformative molecular network analysis including hematological, molecular, metabolic, differential display PCR, histopathological, and immunohistology evaluations.

## 2 Materials and methods

### 2.1 Materials

The roots were obtained from an herbal establishment (Mady Herbs) located in Alexandria, Egypt. Subsequently, these roots were subjected to scrutiny, identification, and validation by a plant taxonomy specialist; Assoc. Prof. Maha Elshamy, Department of Botany, Faculty of Science, Mansoura University, Egypt. A voucher specimen (MU_B_Sc10) was kept at the Department of Botany. The roots obtained were recognized and authenticated as Dolomiaea costus (Falc.) Kasana and A.K. Pandey [Asteraceae], a member of the Asteraceae family commonly known as Saussurea costus (Falc.) Lipsch. and Saussurea lappa (Decne.) Sch. Bip. Sodium nitrite was obtained from El-Gomhouria Company for the Trading of Drugs, Chemicals, and Medical Supplies, located in Alexandria, Egypt. Methanol and formic acid (LC-MS grade) were obtained from Fischer Scientific (UK). Acetonitrile (LC-MS grade) was obtained from Sigma-Aldrich (Germany).

### 2.2 Collection and extraction of S. Costus roots

The ethanol extract was prepared as described by Elshaer et al. (2022), the roots were dried in an oven at 65°C for 3 days and ground to a fairly coarse powder as previously reported by Srivastava et al. (2012). The powder was then carefully kept in sealed containers to prevent exposure to air, ensuring its preservation for subsequent use in the extraction procedure. The powder (400 g) obtained was immersed in 1 L of ethanol (70%) at room temperature and left to soak for 3 days. The solution was subjected to filtration using a Whatman grade-1 filter paper in a funnel under vacuum. Subsequently, the filtrate underwent the rotary evaporation process, in which the liquid was dried and evaporated under conditions of reduced pressure. The crude ethanolic extract of roots of S. costus was obtained and subjected to lyophilization to obtain a dry powder (36 g, 9% wt/wt) (Tag et al., 2016a). Subsequently, the samples were stored at 4°C until use.

### 2.3 Quantitative assessment of chemical metabolites

The identification and detection of active metabolites in the SCREE was achieved by using chemical assays. Various phytochemicals, including phenolics, flavonoids, alkaloids, saponins, and tannins, were identified using established testing methods.

#### 2.3.1 Phenolics detection

The fat-free specimen was subjected to a boiling process with 50 mL of ether to extract the phenolic metabolite, with the extraction process lasting 15 min. A volume of 5 mL of the extract was transferred using a pipette into a 50 mL flask, followed by the addition of 10 mL of distilled water. In addition, a 2 mL aliquot of ammonium hydroxide solution and 5 mL of concentrated amyl alcohol were introduced. The samples were prepared and then allowed to undergo 30 min of reaction to facilitate color development. The measurement was carried out at a wavelength of 505 nm (Edeoga et al., 2005).

#### 2.3.2 Flavonoids detection

A plant sample weighing 10 g was subjected to multiple extractions using 100 mL of 80% aqueous methanol solution at ambient temperature. The entire solution was subjected to filtration using Whatman filter paper with a diameter of 125 mm. Subsequently, the filtrate was transferred to a crucible and subjected to evaporation until complete dryness was achieved using a water bath. The resulting residue was then repeatedly weighed until a consistent weight was obtained (Boham and Kocipai-Abyazan, 1974; Edeoga et al., 2005).

#### 2.3.3 Alkali detection

Five g of the sample was placed in a beaker with a volume of 250 mL, subsequently; 200 mL of the solution (10% acetic acid in ethanol) was added to the beaker and incubated for 4 h at room temperature. The solution was filtered, followed by concentration in a water bath, resulting in a final volume that was one quarter of the initial volume. The extract was subjected to dropwise addition of concentrated ammonium hydroxide until precipitation reached completion. The entire solution was allowed to undergo sedimentation, and subsequently, the resulting solid was gathered and subjected to a rinsing process utilizing a solution of diluted ammonium hydroxide. Following this, the solids were separated from the solution through filtration. The alkaloid, which had been dried and measured, constitutes the residual (Mir et al., 2016).

#### 2.3.4 Saponin detection

A conical flask was used to contain a total of 20 g of the sample, which was then combined with 100 mL of aqueous ethanol solution with a concentration of 20%. The samples were subjected to a heating process for a duration of 4 h while continuously stirred, using a 55°C water bath. The solution was subjected to filtration and the remaining solid was subsequently subjected to another extraction using an additional 200 mL of 20% ethanol. The mixed extracts were concentrated to a final volume of 40 mL using a water bath maintained at 90°C. The concentrated solution was carefully transferred into a separatory funnel with a volume of 250 mL. Subsequently, 20 mL of diethyl ether was added to the funnel and violently agitated. The aqueous phase was retrieved and the ether phase was discarded. Sixty microliters of n-butanol was added. The n-butanol extracts were mixed and subjected to two washes using 10 mL of a 5% aqueous sodium chloride solution. The solution that remained was subjected to heating using a water bath. Following the evaporation process, the samples were dried in an oven until a consistent weight was achieved. Subsequently, the saponin content was calculated (Obadoni and Ochuko, 2002).

#### 2.3.5 Tannin detection

A quantity of 500 mg of the sample was measured and placed in a plastic bottle with a volume of 50 mL. A volume of 50 mL of distilled water was added and stirred for 1 h. The solution was carefully transferred to a volumetric flask with a capacity of 50 mL and then adjusted to the required volume. Next, 5 mL of the filtered solution was transferred using a pipette into a test tube. Subsequently, it was combined with a volume of 2 mL of a solution containing 0.1 M FeCl3 in 0.1 N HCl and 8 mM potassium ferrocyanide. The absorbance measurement was conducted at a wavelength of 120 nm for 10 min (Robinson and Van Burden, 1981).

### 2.4 Evaluation of extract proximate composition

2016 AOAC procedures were used, although with minor modifications, to conduct chemical analysis on extract samples, specifically focusing on protein, lipid and ash content (Al-Zayadi et al., 2023). The crude protein content of the materials was determined by the Kjeldahl procedure. Furthermore, the weight of the powdered material was determined using Soxhlet. The determination of the crude fat content of the extract was performed using petroleum ether. The quantity of ash produced is determined by subjecting the substance to combustion at 500°C for 2 h. The quantification of the carbohydrate content was carried out using the methodology proposed by DuBois et al. (1956). This involved the conversion of carbohydrates into furfural derivatives through dehydration, followed by their reaction with phenol to provide a color that could be measured at a wavelength of 490 nm.

### 2.5 Assessment of phenol metabolites by HPLC analysis

The ethanolic root extract of S. costus was subjected to HPLC analysis using an Agilent 1,260 series to quantify phenolic metabolites (Elshaer et al., 2022). The separation was performed using a Kromasil C18 column of 4.6 mm by 250 mm by 5 mm. The mobile phase consisted of water (A) and 0.05% trifluoroacetic acid in acetonitrile (B), which was flowing at a rate of 1 mL/min. The following linear gradient was sequentially coded into the mobile phase: 12–15 min (85% A), 15–16 min (82% A), 0 min (82% A), 0–5 min (80% A), 5–8 min (60%) and 8–12 min (60%). The wavelength detector was monitored at 280 nm. Specifically, for each of the test solutions, 10 µL of injection volume was used. The column was maintained at a temperature of 35°C (Kolaylı et al., 2010). All standards, including gallic acid, catechin, methyl gallate, chlorogenic acid, caffeic acid, pyro catechol, syringic acid, rutin, coumaric acid, ellagic acid, vanillin, naringenin, ferulic acid, taxifolin and kaempferol, were injected after dissolved in ethanol. The concentration of phenolic metabolites was calculated on the basis of the area under the peak of the standards, and their identities were established by comparing the retention times and UV-vis spectra of the metabolites to those of the standards.

### 2.6 Untargeted metabolomic analysis by ultraperformance liquid chromatography (UPLC/T-TOF–MS/MS)

Analysis was carried out using an Exion LC Triple TOF 5600+ system manufactured by SCIEX in Framingham, MA, USA. The system was run at a temperature of 40°C and was fitted with an X-select HSS T3 C-18 column provided by Waters Corporation in Milford, CT, USA. The column had dimensions of 2.1 × 150 mm and a particle size of 2.5 µm. Furthermore, a pre-column consisting of Phenomenex In-Line filter discs with dimensions of 0.5 µm × 3.0 mm was used. A SCREE solution (50 mg) was prepared by dissolving it in a solvent working solution consisting of MilliQ water, methanol and acetonitrile in a 50:25:25 ratio. The resulting solution was subjected to sonication for 10 min, followed by centrifugation at a speed of 10,000 rpm for 10 min. The stock solution, consisting of 50 μL, was diluted by adding 1,000 µL of the working solvent. SCREE metabolites were subjected to analysis using UPLC/T-TOF–MS/MS in both negative and positive ionization modes (Eissa et al., 2020). Samples (10 µL), with a concentration of 1 μg/μL were introduced into the system using the designated mobile phases. In the negative mode, solvent A consisted of a 5 mM ammonium format buffer at pH 8, prepared using NaOH, with the addition of 1% methanol. In contrast, in the positive mode, solvent A consisted of a 5 mM ammonium format buffer at pH 3, prepared using formic acid, with the inclusion of 1% methanol. In both modes, solvent B consisted of 100% acetonitrile. The gradient elution procedure was executed in the following manner: The chromatographic method employed in this study involved a series of solvent compositions. The solvent composition was initially established at a ratio of 90% solvent A to 10% solvent B for 0–1 min. Subsequently, a linear gradient was applied, transitioning from 90% solvent A to 10% solvent A and 90% solvent B over 1.1–20.9 min. Following this gradient, the solvent composition was held isocratic in a 10% solvent A to 90% solvent B ratio for 21–25 min. Finally, the solvent composition was kept isocratic at 90% solvent A and 10% solvent B for 25.1–28 min. The recorded flow rate was 0.3 mL/min. A blank sample consisting of the working solvent (10 µL) was injected. Metabolites identified were documented using the Analyst TF 1.7.1 program, Peak view 2.2 software (SCIEX, Framingham, MA, USA), and MS-DIAL 3.70 software for data processing (Tsugawa et al., 2015). Mass spectrometry (MS) analysis was performed using a Triple TOF 5600+ system with a Duo-Spray source working in the electrospray ionization (ESI) mode, manufactured by AB SCIEX in Framingham, MA, USA. The mass range covered during the analysis ranged from 50 to 1,100 m/z. The process of characterization of compounds involved generating a candidate formula while adhering to a mass accuracy restriction of 10 ppm. Additionally, other factors such as retention time (Rt), MS2 data, databases, and reference literature were taken into account (Singh et al., 2017).

### 2.7 Experimental design

Adult male Wistar Albino rats weighing 160–180 g (9–11 weeks old) were donated by the Animal House of the Institute of Graduate Studies and Research at Alexandria University in Alexandria, Egypt. The Local Ethics Committee and the Animal Research Committee authorized the study design and the Laboratory Animal Care Guidelines from the National Institutes of Health (NIH) were followed when handling the animals. (AU14-210126-2-3). The animals were housed in cages with good ventilation and 12-h light/dark cycles every day that ranged in temperature from 20° to 25°C. The rats were supplemented daily with a standard pelleted diet and water *ad libitum*. The animals underwent 2 weeks of monitoring before the study to ensure their successful adaptation. Randomly, eight equal groups of rats were formed, each of seven: Group 1 received distilled water (1 mL/kg body weight), Group 2 received SCREE (200 mg/kg body weight) (Tag et al., 2016a), Group 3 received SCREE (400 mg/kg body weight) (Tag et al., 2016a), Group 4 received SCREE (600 mg/kg body weight) (Tag et al., 2016a), Group 5 was treated with sodium nitrite (NaNO2, 6.5 mg/kg body weight, 1/25 LD50) (Fouad et al., 2017; Abo-EL-Sooud et al., 2019), Groups 6, 7 and 8 received NaNO2 (6.5 mg/kg body weight, 1/25 LD50) in combination with SCREE (200, 400, and 600 mg/kg body weight), respectively. Rats were given NaNO2 and SCREE daily by oral gavage for 28 days. After the experiment, the rats fasted for 12 h before taking blood samples. The rats were hypnotized with isoflurane, sacrificed and then blood, livers, and kidneys were collected for different analyzes.

### 2.8 Body weight, body weight gain (BWG) and weight of the organs

Rat weights were observed before and after the experimental period, and BWG was also calculated. After scarification, both the liver and kidney weight of the rat were recorded.

### 2.9 Blood and serum samples

Blood samples were collected through heart piercing and allowed to coagulate for 30 min at 25°C prior to centrifuging for 15 min at 3,000 xg, and the clear serum was carefully separated and stored at −20°C for further analysis. Other blood samples were obtained in EDTA tubes for full blood count (CBC) analysis using an automated analyzer (ABX Micros 60 automated hematologic analyzer, HORIBA ABX Diagnostic Company, France).

### 2.10 Biochemical investigations of liver function biomarkers

Initially, an examination is conducted to assess the impact of SCREE on liver function in Albino rats treated with sodium nitrite. Rat serum was tested using kits available for purchase from the Cairo Biodiagnostic Company to assess the action of liver aminotransferase action AST; EC 2.6.1.1, CATALOG NO. AS 10 61 and ALT; EC 2.6.1.2, CATALOG NO. AL 10 31, alkaline phosphatase (ALP; EC 3.1.3.1) CATALOG NO. AP 10 20, albumin (CATALOG NO. AB 10 10) and gamma-glutamyl transferase (GGT) (CATALOG NO. GGT 124030) as an additional to the total bilirubin level (CATALOG NO. BR 1111).

### 2.11 Biochemical investigations of kidney function biomarkers

Second, a comprehensive evaluation is performed to analyze the influence of SCREE on renal function of Albino rats that have received sodium nitrite. The determination of serum levels of urea (CATALOGUE NO. URE 18100), creatinine (CATALOGUE NO. CRE 106100) and uric acid (CATALOGUE NO. UA 119240) was conducted using commercially available kits provided by Egy Chem for Lab Technology Company, located in Cairo, Egypt.

### 2.12 Biochemical investigation of the lipid profile

At the same time, we examined the impact of SCREE on the lipid profile to investigate its hepatoprotective effect against sodium nitrite-induced damage. A commercial kit from Biodiagnostic Company, Cairo, Egypt, was used to estimate the lipid profile, which included the concentrations of total cholesterol (CATALOG NO. CH 12 20), triglycerides (CATALOG NO. TR 20 30), high-density lipoprotein cholesterol (HDL-C) (CATALOG NO. CH 12 30), low-density lipoprotein cholesterol (LDL-C) (CATALOG NO. CH 12 31), and the very low-density lipoprotein -cholesterol (VLDL-C) concentration was calculated from the following equation.

VLDL−C=TG/5

### 2.13 Determination of the serum alpha-fetoprotein tumor marker

In this study, we investigated the hepatoprotective effect of SCREE (Sodium Nitrite-induced liver damage) by examining its impact on the level of AFP (Alpha-Fetoprotein). Elevated levels of alpha-fetoprotein (AFP) can be observed in benign and malignant liver conditions. Alpha-fetoprotein (AFP) is a tumor marker for the detection and diagnosis of liver, testicles, and ovary cancers and was estimated using kits from the Egyptian Company for Biotechnology (SAE) (CATALOG NO. 1317 001). This assay is a solid-phase enzyme immunoassay in the “sandwich” style that is based on an antigen-antibody complex that is created when a sample containing AFP is added to the wells, where it binds to the two antibodies.

### 2.14 Determination of C-reactive protein

To investigate SCREE’s renal protective effect against sodium nitrite-induced damage, we looked at how it affected the level of CRP. The levels of C-reactive protein have been observed to increase in correlation with deterioration of renal function. Nephelometry was used to determine an inflammation marker in rat serum based on CRP (CATALOG NO. 52009002) and was estimated using a commercial kit from AGAPPE Diagnostics Switzerland GmbH.

### 2.15 Molecular analysis using quantitative real-time PCR (Q-RTPCR)

#### 2.15.1 Extraction of RNA and production of cDNA

According to the manufacturer’s instructions (Wang et al., 2009), we used Trizol reagent (easy-BLUETM, INTRON Biotechnology, Korea, CATALOG No.17061) to extract total RNA from liver and kidney tissues. Finally, the total RNA was eluted using 20 L of RNase-free water. RNA concentrations and purity were evaluated using a UV spectrophotometer (Nanodrop 8,000, Thermo Scientific, USA). Reverse-transcribed total RNA was used to create cDNA in a 20-L reaction with the following components: A total of 20 L of sterile water, 3 L of total RNA, 2.5 L of dNTP (10 mM), 2.5 L of buffer (10x), 0.3 L of reverse transcriptase, and 5 L of oligo-dT primer were added to the reaction mixture. The completed mixture of the reaction was put into a thermal cycler and subjected to the following cycle: 2 h at 37°C, 20 min of inactivation at 65°C, and then storage at −20°C.

#### 2.15.2 Real-time polymerase chain reaction

The determination of the liver and kidney (IL-4), (P53), (BCL-2), and (TNFα) gene expressions by qRTPCR was performed according to the procedure of (Fan and Robetorye, 2010). Q-RTPCR was performed using an (SYBR^®^ Green PCR Master Mix Kit, CATALOG NO. RT500S) (Fermentas, USA) to examine the expression levels of the genes (IL-4), (P53), (BCL-2), and (TNF-α) genes, both in liver and kidney tissues, the primer sequences of the genes tested are shown in (Table 1). Internal controls were performed using the housekeeping gene, -actin. Several reactions were carried out in a 25 μL mixture, which contained 1 μL of 10 pmol/μ1 of each primer, 1 μL of template cDNA (50 ng), 12.5 μL of 2X SYBR Green PCR Master Mix, and 9.5 μL of nuclease-free water. Before loading the samples into the rotor wells, the samples were spun and a triple of each sample was tested (Supplementary Table S1). During the completion of the 10 min of 95° amplification, there were 40 cycles of denaturation at that temperature for 15 s, followed by 30 s of annealing at 60° and 30 s of extension at 72°. The melting curves were acquired after the cycle process to stop the manufacture of generic goods. Data collection took place during the extension process. A RotorGene 6,000 (QIAGEN, ABI System, USA) was utilized for the reaction. The primers utilized in this study are listed in (Table 1). The gene expression results were analyzed using the 2-ΔΔCTmethod (Livak and Schmittgen, 2001). For three separate amplifications, the data were reported as mean fold changes ±standard error.

**TABLE 1:** The main metabolites present in the ethanolic extract of the roots of S. costus (SCREE) were tentatively identified using UPLC/T-TOF–MS/MS in positive and negative ionization modes.

Title	RT (min)	Precursor (m/z)	Area	Error (PPM)	Adduct	Reference (m/z)	Formula	Ontology
D-(+)-Malic acid	0.8788	133.0137	5064285	−0.8	[M-H]-	133.01425	C_4_H_6_O_5_	Beta hydroxy acids and derivatives
Gluconate	0.9553	195.05	2141325	3.1	[M-H]-	195.05103	C_6_H_12_O_7_	Medium-chain hydroxy acids and derivatives
S-Lactoylglutathione	0.9894	380.0954	821,613	0.6	[M + H]+	380.11221	C_13_H_21_N_3_O_8_S	Oligopeptides
Choline	1.0284	104.1072	3664216	−0.3	[M]+	104.10645	C_5_H_14_NO	Cholines
L-β-homotryptophan-HCl	1.0922	219.0265	2495170	0.4	[M + H]+	219.11281	C_12_H_14_N_2_O_2_	Beta amino acids and derivatives
Sucrose	1.1089	341.1085	8329249	0.7	[M-H]-	341.10895	C_12_H_22_O_11_	O-glycosyl compounds
L-proline	1.1432	116.0685	6683844	14.6	[M + H]+	116.0706	C_5_H_9_NO_2_	Proline and derivatives
Adenosine 3':5′-cyclicmonophosphate	1.5327	330.0566	1103257	6.5	[M + H]+	330.05978	C_10_H_12_N_5_O_6_P	3′,5′-cyclic purine nucleotides
L-5-Oxoproline	1.6112	130.0489	882,512	1.8	[M + H]+	130.04987	C_5_H_7_NO_3_	Alpha amino acids and derivatives
Apigenin-7-O-glucoside	7.7027	431.101	3585188	−4.3	[M-H]-	431.09836	C_21_H_2_0O_10_	Flavonoid-7-O-glycosides
Isocitrate	8.5081	191.035	2382439	−0.2	[M-H]-	191.01973	C_6_H_8_O_7_	Tricarboxylic acids and derivatives
3′4′5 7-tetrahydroxyflavanone	10.9264	289.1409	1126044	0.5	[M + H]+	289.07068	C_15_H_12_O_6_	Flavanones
Acacetin	11.0540	285.0754	1473894	1	[M + H]+	285.07574	C_16_H_12_O_5_	4′-O-methylated flavonoids
Apigenin	13.3821	269.0452	38379868	0.8	[M-H]-	269.04553	C_15_H_10_O_5_	Flavones
Daidzein	16.8737	255.1357	3149348	0.3	[M + H]+	255.06519	C_15_H_10_O_4_	Isoflavones
γ-Linolenic acid	19.0706	277.2178	15435236	−0.7	[M-H]-	277.21732	C_18_H_30_O_2_	Lineolic acids and derivatives

#### 2.15.3 Differential display-polymerase chain reaction (DD-PCR)

DD-PCR is an effective method used to investigate the up- and downregulated genes that the treatment affects compared to the control, and DD-PCR was approached on cDNA extracted from the examined animals, blood or tissues, as a template. Furthermore, the conditions for the reaction were carried out according to (Hafez et al., 2013). The total reaction volume is 25 μL containing 2.5 μL 10X Taq buffer, 2.5 μL MgCl2, 2.5 μldNTPs, 1 U Taq DNA polymerase (CAT. No. MB101-0500), 3 μL of 10 pmol of arbitrary primers separately and 2 μL of each cDNA and finally 12 μL of sterile dH2O. The amplification program looked like this; one cycle at 94°C for 5 min (hot start), followed by 40 cycles at 94°C for 1 min, 35°C for 1 min, and 72°C for 1min, and finally extension step at 72°C for 10 min. The down- and up-expressed genes were removed from the 2% agarose gel after the PCR results were imaged using a gel documentation system (Gel Doc, 2000). The sequences of primers used are listed in Table 1, and the examinations were carried out on the quantity and size of the amplified fragments according to (Liang and Pardee, 1992; Hamad et al., 2018).

### 2.16 Histological and immunohistochemical investigation

For histological analysis using hematoxylin and eosin stain, the kidney and liver were fixed in a 10% buffer neutral formalin solution and handled to create serial paraffin segments, which were then viewed with a light microscope according to (Bancroft et al., 1990). Using DAB staining and the Avidin-Biotin Peroxidase (ABC) immunohistochemical method, the transforming growth factor-beta (TGF-β) protein (TGF-) in liver tissue was determined in deparaffinized sections (5 μm) (Buchwalow and Böcker, 2010).

### 2.17 Statistical evaluation

All data are shown as mean ± SE. All analyzes were performed using the Social Sciences Statistical Package, the parameters calculated for the Social Sciences (SPSS) program Version 16.0. Multiple comparisons were analyzed using one-way analysis of variance (ANOVA) followed by Tukey’s multiple comparison test to assess the data and determine how the groups differed from each other using GraphPad Prism nine software (GraphPad Software, San Diego, CA, USA). The *p* values were assigned significant if < 0.05 (*, * and ** representing *p* < 0.05, *p* < 0.01, *p* < 0.001 and *p* < 0.0001, respectively).

## 3 Results and discussion

### 3.1 Chemical metabolites and proximate analysis of roots of S. Costus

We focus primarily on exploring the phytochemical metabolites of SCREE. Toward this end, we conducted several chemical and analytical assays to gain valuable insight into the root composition and its potential pharmacological applications. As shown in (Supplementary Figure S1), our analysis revealed that SCREE consists of 0.26 g/mL of proteins, 19.26 mg/g of crude lipids, and 3.29 mg/g of carbohydrates, as well as a ash content of 4.72%. While the protein content may be relatively low, the substantial presence of crude lipids and carbohydrates, along with the mineral composition, underscores the potential nutritional and medicinal value of the roots of S. costus. The relatively high presence of crude lipids suggests the presence of fats, which could be important for energy storage and the absorption of fat-soluble vitamins. The carbohydrate content provides information on the energy potential of these roots, and the ash content reflects the mineral composition, which is important for both nutritional and medicinal considerations, as minerals play a crucial role in various physiological functions. Further exploration of metabolite constituents revealed that S. costus roots show notable concentrations of phenolic (58.85 mg/g) and flavonoid metabolites (97.15 mg/g). Phenolic and flavonoids are well recognized for their antioxidant properties (Tungmunnithum et al., 2018), indicating that these roots may possess potential health benefits and therapeutic applications. Additionally, SCREE demonstrated a considerable concentration of alkaloids (0.267 mg/g) and saponins (27.35 mg/g), suggesting the chemical diversity of the roots of S. costus that can facilitate a range of applications, from dietary supplementation to the development of traditional and alternative medicine. Taken together, the presence of macronutrients such as lipids, carbohydrates, and bioactive metabolites, notably phenolics and flavonoids, in SCREE underscores the multifaceted applications of the roots of S. costus in dietary and medicinal contexts. The findings of the phytochemical analysis indicate that the plant exhibits promising characteristics regarding its potential anti-inflammatory, antibacterial, and antioxidant capabilities (Kumar and Pundir, 2022). The primary source of natural antioxidants is derived from plants, predominantly in the form of phenolic chemicals, including flavonoids, phenolic acids, and tocopherols (Asif, 2015). Flavonoids exhibit notable antioxidant properties, anti-inflammatory and anticarcinogenic effects (Saleh-e-In et al., 2016; Vignesh et al., 2021). Tannins are multifaceted metabolites that exhibit a wide range of pharmacological properties, including antioxidant, antibacterial, and anti-inflammatory effects (Saif-Al-Islam, 2020). Saponins have been found to exhibit protective effects against conditions such as hyperglycemia, hypercholesterolemia, and hypertension (Parama et al., 2020), while also possessing antibacterial, anti-inflammatory, and wound healing capabilities (Fromm and DeGolier, 2021). Alkaloids have been documented to possess potent analgesic properties, as well as exerting effects such as reducing fever, lowering blood pressure, combating fungal infections, reducing inflammation, preventing fibrosis, promoting stimulation, inducing anesthesia, and inhibiting the growth of various bacterial strains (Boro et al., 2021; Heinrich et al., 2021). The results of our study indicate that SCREE contains beneficial antioxidants and anti-inflammatory metabolites. Furthermore, it is suggested that SCREE has the ability to reverse oxidative stress and positively impact hematologic parameters. This indicates that the plant has the potential to serve as a valuable source of natural antioxidants and substances that enhance blood function.

### 3.2 Evaluation of phenolic metabolites in SCREE

Based on the approximate analysis, our results indicated that SCREE has a substantial concentration of phenolic metabolites. Consequently, our investigations were extended to explore the phenolic metabolites of SCREE. In this regard, we conducted phenolic-targeted HPLC analysis for the SCREE. As indicated in (Supplementary Figure S2), our analysis revealed that SCREE exhibits considerable concentrations of gallic acid, emerging as the dominant phenolic metabolite with a concentration of 7881.15 μg/g. The following are closely followed by chlorogenic acid at 3,265.11 μg/g and naringenin at 1,197.63 μg/g. Although other metabolites, such as cinnamic acid, ferulic acid, vanillin, taxifolin, methyl gallate, and ellagic acid, were found in the extract, their concentrations were comparatively lower, ranging from 198.16 μg/g to 71.85 μg/g (Supplementary Figure S2). Caffeic acid and syringic acid were also present in trace amounts (59.77 μg/g and 51.71 μg/g, respectively). On the contrary, kaempferol, coumaric acid, catechin, pyrocatechol, and rutin were not detected in the extract (Supplementary Figure S2). These findings are of significance, as phenolic metabolites are renowned for their diverse biological activities, including antioxidant and anti-inflammatory properties. The presence of multiple phenolic metabolites, especially gallic acid and chlorogenic acid, suggests that SCREE may offer potential health benefits. The detected amounts of caffeic acid and syringic acid, although minimal, contribute to the overall phenolic diversity of the extract (Rahman et al., 2022). Collectively, these results shed light on the phenolic composition of SCREE and support the pharmacological and therapeutic potentials of SCREE.

### 3.3 Untargeted metabolomic analysis by UPLC/T-TOF-MS/MS analysis

**FIGURE 1:**
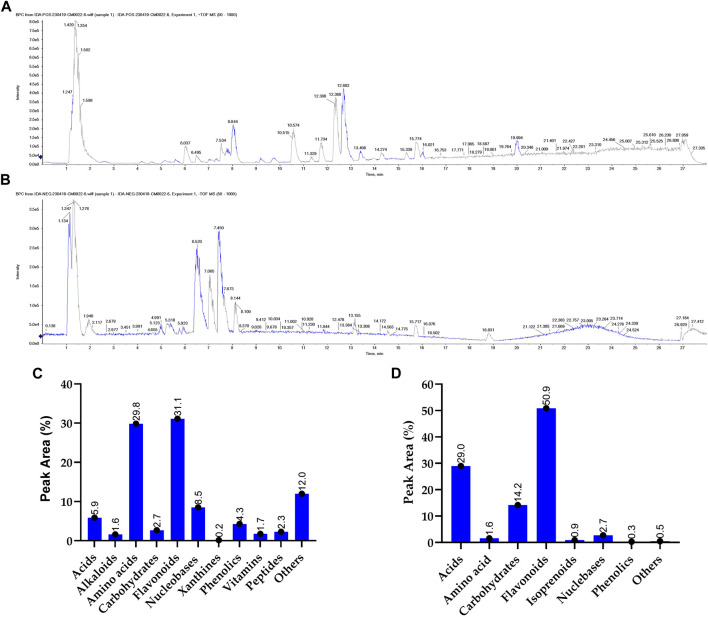
Untargeted metabolomic analysis of SCREE by UPLC/T-TOF-MS/MS analysis. The base peak chromatogram of SCREE in the positive **(A)** and negative **(B)** electrospray ionization modes. The classification of the identified primary metabolites in the positive **(C)** and negative **(D)** ionization modes.

Metabolomic analysis is a vital tool to explore the bioactivity of plant extracts, to elucidate their mode of action and to discover key metabolites. By comprehensively profiling and identifying small molecules in these extracts, metabolomics helps uncover the complex chemical compositions and interactions responsible for their biological effects. This approach is essential for understanding how plant metabolites impact human health, drug discovery, and uncovering potential therapeutic agents or bioactive metabolites from natural sources. To gain more insight into the exact metabolites in the SCREE extract, we performed a detailed untargeted metabolomic analysis utilizing UPLC/T-TOF-MS/MS. Analysis was carried out in both positive and negative modes, leading to the identification of 187 metabolites (Figure 3). In the positive ionization mode, a total of 104 metabolites have been detected, covering a wide spectrum of 11 distinct classes, including acids (5.9%), amino acids (30%), alkaloids (1.6%), nucleobases (8.52956%), xanthines (0.15%), vitamins (1.74%), carbohydrates (2.7%), peptides (2.3%), phenolic metabolites (4.3%), flavonoids (31%) and several other miscellaneous metabolites (12%) (Figure 1; Supplementary Table S4). This diverse array of metabolite classes highlights the comprehensive nature of the analysis, with various metabolites from different classes identified (Figure 1). Our findings underscored that more than 31% of the identified metabolites belong to the flavonoid class. Within this category, two subgroups have been distinguished: one comprising 15 flavonoid metabolites and the other comprising 15 flavonoid–O-glycosides (Supplementary Material). Among flavonoids, four specific flavonoids emerged as the most prevalent metabolites, including daidzein (8.2%), 3, 5, 7-trihydroxy-4′-methoxyflavone (6.7%), acacetin (3.8%), and 3′,4′,5,7-tetrahydroxyflavanone (2.9%). Regarding flavonoid-O-glycosides, apigenin-7-O-glucoside stands out as the most abundant metabolite within the group of 15 metabolites (1.7%). Regarding the detected amino acid metabolites, 15 different amino acid metabolites have been detected, which constitute approximately 30% of the total detected metabolites. L-proline was the most prevalent amino acid metabolite (17.5%), followed by L-ß-homotryptophane (6.5%) and L-5-oxoproline (2.3%). A set of 11 nucleobase-based metabolites was also detected in the extract, which represented about 9% of the total detected metabolites. Adenosine-3′,5′-cyclic monophosphate represented the most prevalent metabolite (2.9%), followed by 2′-deoxycytidine (1.9%), and xanthosine-5′-monophosphate (1.4%). The detected small peptide metabolites contributed approximately 2.3% of the total detected metabolites and comprised two metabolites, S-lactoylglutathione (2.16%) and leupeptin (0.12%). Phenolic metabolites detected represented approximately 4.3% of the total detected metabolites and were composed of seven metabolites. The metabolite most prominently presented was the coumarin-based metabolite scopoletin (1.61%), followed by 4-aminophenol (1.3%). Acid-based metabolites represented approximately 5.9% of the total detected metabolites, consisting of 17 different metabolites. Chlorogenic acid, a cinnamoyl-based acid, was shown to be the most prevalent metabolite in this class (1.67%), followed by N-acetylneuraminate (1.1%) and urocanic acid (0.69%).

Carbohydrate-based metabolites represented approximately 2.7% of the total detected metabolites and comprised four metabolites. α-Lactose represented the lead metabolite with the highest peak area (1.5%), followed by α-D-glucose-1-phosphate (0.91%). Furthermore, our analysis revealed a set of three xanthine metabolites (total PA%, 0.158) consisting of 3-methylxanthine, 1,3-dimethylurate and uric acid, with 3-methylxanthine being the predominant xanthine metabolite (0.069%). Two alkaloid metabolites were also identified (total PA%, 1.63%), scoulerin being the most prevalent (1.19%). Additionally, miscellaneous metabolites from various classes contributed approximately 12% to the total metabolites, consisting of approximately nine metabolites. In this class of metabolites, choline was the most prevalent (9.6%), followed by Hinokitiol (0.73%). Furthermore, a set of three vitamins were also detected that constitute approximately 1.6% of the total detected metabolites, including pyridoxamine (vit B6), calciferol (vit D2), and nicotinamide (vit B3) (Figure 1).

The negative mode of the SCREE UPLC/T-TOF-MS/MS analysis has successfully demonstrated the detection of 84 metabolites, covering a diverse range of eight classes, including acids, amino acids, carbohydrates, alkaloids, isoprenoids, nucleosides, phenols, and others (Figure 1; Supplementary Table S5). Similar to the positive mode analysis, our findings highlighted that more than 50% of the detected metabolites belong to the flavonoid class, with 20 distinct metabolites identified. The detected flavonoid-based metabolites comprised 11 flavonoid metabolites and nine flavonoid O-glycoside metabolites. Among the flavonoid metabolites, apigenin represented the most prevalent metabolite (37.9%), followed by pelargonidin-3-O-glucoside (3.7%) and apigenin-7-O-glucoside (3.5%). Acid-based metabolites represented the second most detected metabolites with 29% of the total detected metabolites. The detected acid-based metabolites comprised 21 different metabolites. γ-Linolenic acid was the most prevalent metabolite in this class with 15.2% of the total detected metabolites, followed by D- (+) -malic (5%) and isocitrate (2.3%). Our analysis also revealed that the SCREE extract contains a set of 11 carbohydrate-based metabolites with 14.2% of the total detected metabolites. Among this class of metabolites, sucrose was the most prominent metabolite (8.2%), followed by gluconate (2.1%). Nucleobase-based detected metabolites represented approximately 2.7% of the total detected metabolites, with eight metabolites identified in this class. Inosine-5′-monophosphate represented the most prominent metabolite in this class (1.3%), followed by 2′-deoxyinosine (0.8%). The amino acid metabolites represented only 1.6% of the total detected metabolites, with eight identified metabolites. DL-5-Hydroxylysine was the main amino acid detected in this group (0.35%). Our analysis also detected a set of three isoprenoid metabolites, which represented about 1% of the total detected metabolites. Gibberellin A4 represented the main isoprenoid metabolite with a peak area of 0.5%. Phenolic-based metabolites represented approximately 0.3% of the total detected metabolites, with three identified metabolites. The most abundant phenolic metabolite was syringaldehyde, accounting for 0.13%. Lastly, a set of eight miscellaneous metabolites was also detected, which represented less than 0.5% of the total detected metabolites. These metabolites represent different classes of metabolites including xanthine, peptides, and coumarin-based metabolites.

**FIGURE 2:**
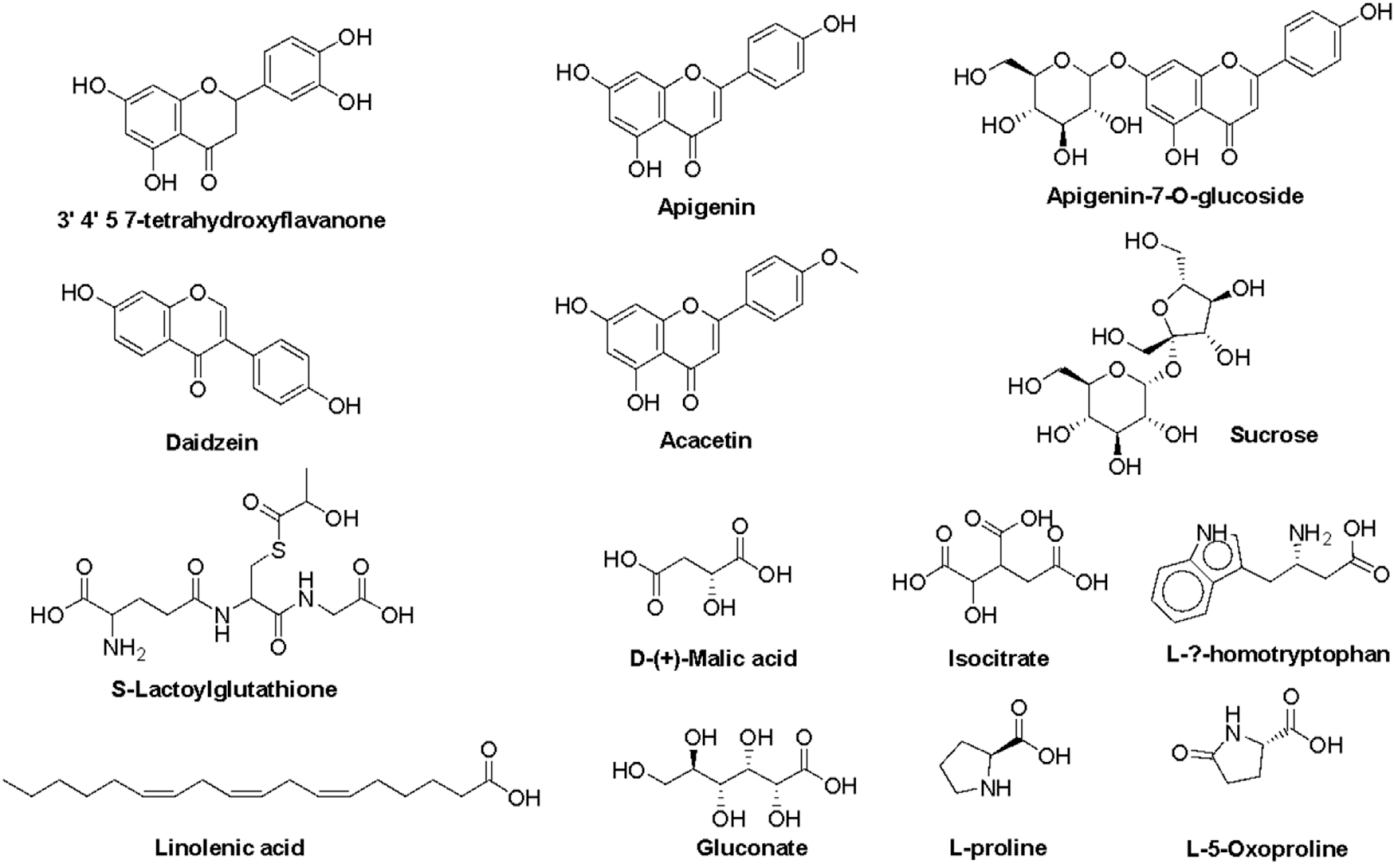
Representative metabolites tentatively detected in the roots of the ethanol extract of S. costus using a UPLC/T-TOF–MS/MS spectrometer.

The most prominent metabolites detected by untargeted metabolomic analysis are presented in (Table 1). Our LC/MS-MS analysis revealed a set of 16 metabolites that were identified with more than 2% of the total detected metabolites (Figure 2). These metabolites possess diverse biological activities, including anti-inflammatory, antioxidant, and antitumor effects, making them valuable candidates for natural remedies and complementary agents in the treatment of a wide spectrum of health concerns. Therefore, the pronounced biological activity of the SCREE extract attributed to the metabolites is prevalent. Our metabolomic analysis revealed that SCREE possesses a high content of flavonoids, including apigenin, apigenin-7-O-glucoside, daidzein, acacetin, and 3′,4′,5,7-tetrahydroxyflavanone. Apigenin, the most prominent metabolite in the SCREE extract, is renowned for its robust antioxidant and anti-inflammatory characteristics (Romanova et al., 2001; Wang et al., 2014b; Kashyap et al., 2022). It also demonstrates its complications in CNS-related disorders such as multiple sclerosis (Ginwala et al., 2019). Furthermore, apigenin demonstrated antitumor activity both *in vitro* and *in vivo*. It triggers cell apoptosis, induces cell cycle arrest, suppresses cell migration and invasion, and stimulates autophagy (Lotfi and Rassouli, 2024). The SCREE extract also showed a considerable content of daidzein, which functions as a phytoestrogen. It interacts with human estrogen receptors, influencing estrogen sulfation and potentially affecting hormone-related conditions such as breast cancer. Moreover, it exhibits antiviral characteristics and provides lung protection (Poschner et al., 2017). Daidzein demonstrates a potential anti-inflammatory effect by modulating NO levels, pro-inflammatory cytokines (IL-6, TNF-α), inflammatory indicators (COX-2, iNOS), and effectively suppressing NF-κB signaling. Acacetin demonstrates various biological activities, such as its potential as an antidiabetic agent, making it promising for diabetes management by improving insulin sensitivity and blood sugar regulation. It also has potential for cancer prevention, cardiac protection, neuroinflammation control, and antimicrobial effects (Singh et al., 2020). Furthermore, it exhibits potent anti-inflammatory effects by suppressing the expression of pro-inflammatory cytokines, including nitric oxide synthase (iNOS) and COX-2 (Ouyang et al., 2021). Apigenin-7-O-glucoside is known for its various biological activities, including its functions as an antioxidant, effectively countering oxidative stress, and as an anti-inflammatory agent, helping alleviate inflammation-related problems (Armeni et al., 2014; Gallo and GГЎmiz, 2023). Similarly, 3′,4′,5,7-tetrahydroxyflavanone is known for its potential biological activities, which can include antioxidant and anti-inflammatory effects (Lin et al., 2008). Furthermore, SCREE showed a considerable content of amino acid metabolites, including L-proline, L-ß-homo-tryptophan, and L-5-oxoproline. L-proline, the second highly detected metabolite, plays a crucial role in various biological activities, such as collagen synthesis, and contributes to wound healing and neurotransmitter regulation (Li et al., 2019). Another detected amino acid metabolite, L-ß-homotryptophan, serves as a precursor to serotonin, contributing to the modulation of growth and the immunometabolic state (Roager and Licht, 2018). Its structural similarity to tryptophan gives rise to the intriguing possibility of interacting with similar metabolic pathways and receptors, potentially affecting functions linked to immune regulation and neural processes. L-5-oxoproline is also recognized for its anticancer potential, which has been linked to its antioxidant properties, cell cycle regulation, immune system modulation, and chemo-preventive potential (Sasaki et al., 2015). Acid-based metabolites also represented a substantial content of SCREE extract, including γ-Linolenic acid, D-(+)-Malic acid, and Isocitrate. γ-Linolenic acid plays a pivotal role in modulating inflammatory responses. It is essential to produce anti-inflammatory eicosanoids and regulation of gene expression, affecting immune function and cell apoptosis (Kapoor and Huang, 2006). D-(+)-Malic acid exhibits notable biological activities, mainly known for its hepatoprotective effects (Madrigal-Santillán et al., 2014). Additionally, it plays a role in the regulation of acidity in the body and participates in the citric acid cycle, an essential metabolic pathway (Tuncel et al., 2015). Isocitrate demonstrates, as an antioxidant, the ability to combat oxidative stress by participating in the citric acid cycle, which is essential for energy production and the neutralization of harmful free radicals (White and Someya, 2022). The SCREE extract exhibited several carbohydrate-based metabolites, including sucrose and gluconate. Sucrose is known for its multifaceted activity, including its role as a readily available energy source in metabolism and its importance in various biological processes such as glycolysis and cell respiration (Huestis, 2007). Gluconate exhibits robust anti-inflammatory characteristics through its ability to efficiently decrease the production of inflammatory chemokines (Nii et al., 2019). Other metabolites have also been detected in the SCREE extract, including choline and S-lactoylglutathione. Choline is known for its liver protective and cholesterol-lowering effects (Mehedint and Zeisel, 2013). It also serves as a precursor to acetylcholine, affecting brain development, cognition, the gut microbiota, and metabolic health (Gallo and GГЎmiz, 2023). Although S-lactoylglutathione exhibits a significant role as a precursor to the important antioxidant glutathione. It contributes to cell protection against oxidative stress, detoxification processes, and overall maintenance of cell health (Armeni et al., 2014).

### 3.4 Evaluation of the effect of SCREE on organ weight changes and body weight gain

**FIGURE 3:**
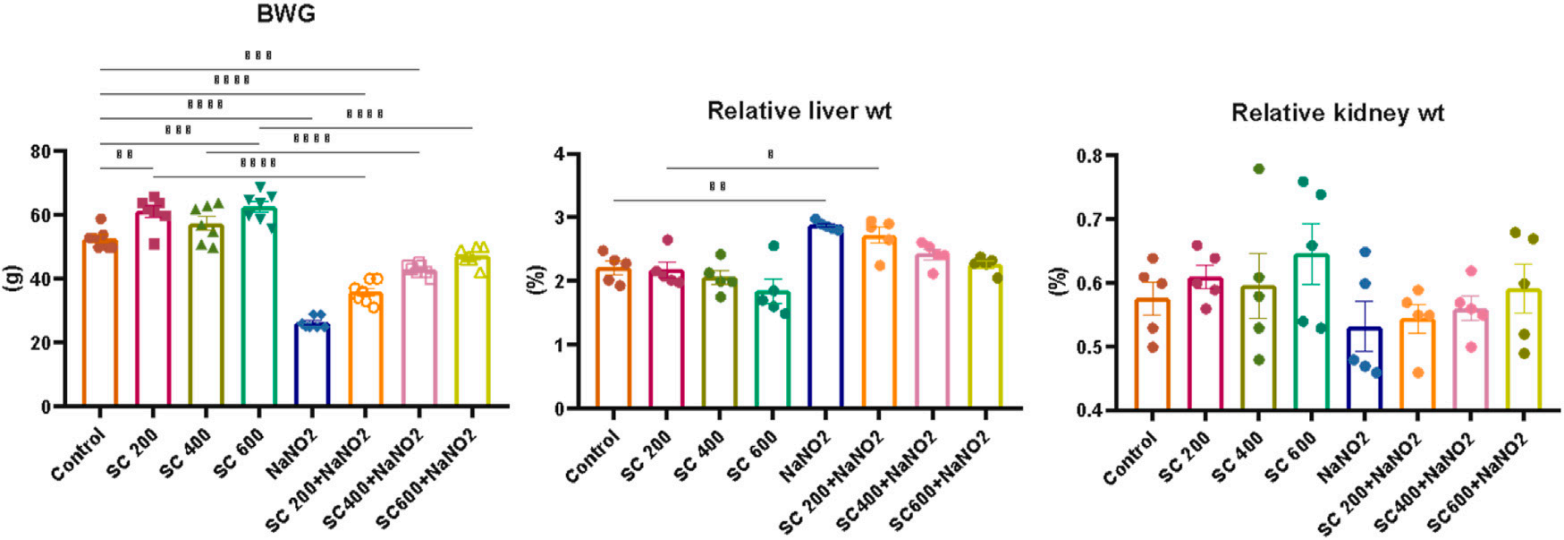
Effects of SCREE administration on organ weight, body weight, and body weight gain (BWG) of NaNO2-treated male rats. The results of the statistical tests were reported as mean ± SEM (n = 7). Multiple comparisons were analyzed by one-way ANOVA followed by Tukey’s multiple comparison test. Differences between groups were considered significant when *p* < 0.05 (*p < 0.05, **p < 0.01, ***p < 0.001 and ****p < 0.0001). Significant data included for each group compared to the control group and for SCREE groups (SCREE 200, SCREE 400, SCREE 600) each compared to its respective treated group (SCREE 200+ NaNO2, SCREE 400+ NaNO2, SCREE 600+ NaNO2).

Our initial examinations evaluated the impact of SCREE on body and organ weight, focusing on the liver and kidneys. As depicted in (Figure 3), rats treated with NaNO2 for 28 days showed a significant reduction in body weight, weight gain, and kidney weight, but also an elevation in liver weight compared to the control group. These reductions in body weight can be attributed to reduced food intake (Grant and Butler, 1989) or lack of vitamin C (Uchida et al., 1990), or it could be due to an increase in catabolic processes caused by an increase in NaNO2 levels in the body. These findings correspond to those of Poter et al. (Porter et al., 1993), who reported that weight loss was due to a reduction in food intake, a disturbance in hormonal equilibrium, and sodium nitrite therapy has a direct cytotoxic impact. In addition, body weight was found to increase in rats treated with monosodium glutamate, while it decreased in rats that consumed NaNO2 compared to normal rats (Helal et al., 2017). Furthermore, a significant increase in the hepatosomatic index was also caused by the toxic effects of NaNO2 on the nucleic acids, glycogen, lipids and proteins found in the liver, which are responsible for liver growth (Srinivasan and Radhakrishnamurty, 1988; Dikshith et al., 1991). On the other hand, our results revealed that SCREE supplementation with NaNO2 has significant and dose-dependent improvements in body weight, body weight gain, and liver weight, while it exhibits insignificant effects on kidney weight compared to the control group. The improvement observed by SCREE treatment is consistent with the studies by abdul-hussein (2019) and Ahmed (Ahmed, 2017) that showed that rats given ethanolic extract of S. costus exhibit significantly (*p* < 0.05) higher final BW and BWG values compared to the control group. Thyroid hormones play an essential role in growth, development, reproduction, and stress response (Peter, 2011). The decrease in growth rate among the groups exposed to nitrite could be linked to thyroid hormone levels (Ciji et al., 2013). The weight gain observed in our study can be attributed to the presence of tryptophan in SCREE, as this amino acid serves as a crucial factor in the regulation of nutrient metabolism and the promotion of increased body weight. Tryptophan is a vital precursor for neurotransmitters and metabolic regulators that regulate nutrient metabolism (Ruan et al., 2014).

### 3.5 Assessment of SCREE impact on hematological parameters

**FIGURE 4:**
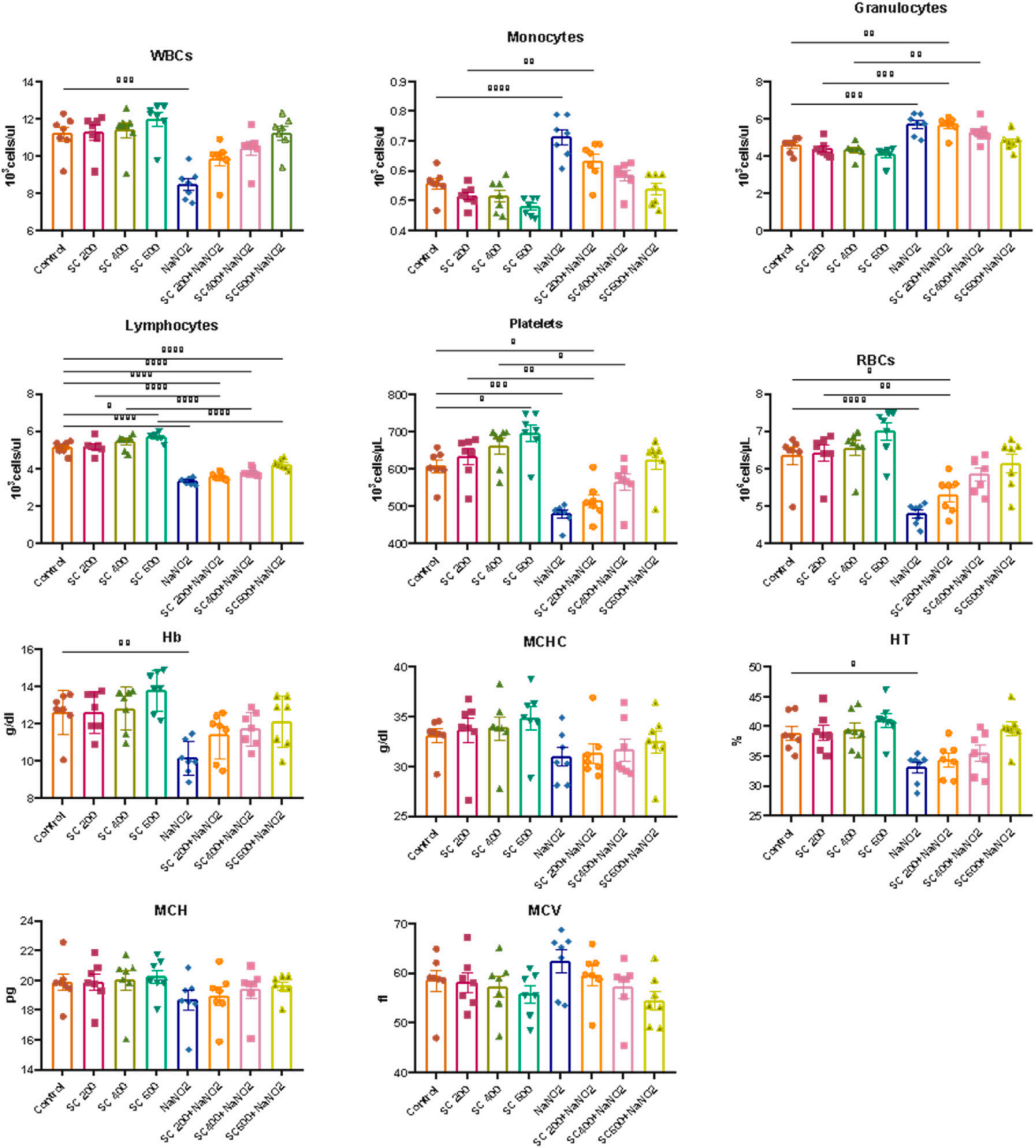
Effects of SCREE on the hematological parameters of male rats treated with NaNO2. The results of the statistical analysis were reported as mean ± SE (n = 7). Multiple comparisons were analyzed by one-way ANOVA followed by Tukey’s multiple comparison test. Differences between groups were considered significant when *p* < 0.05 (*p < 0.05, **p < 0.01, ***p < 0.001 and * ***p < 0.0001). Significant data included for each group compared to the control group and for SCREE groups (SCREE 200, SCREE 400, SCREE 600) each compared to its respective treated group (SCREE 200+ NaNO2, SCREE 400+ NaNO2, SCREE 600+ NaNO2).

Next, we explore the effect of SCREE on the hematological parameters of male rats treated with NaNO2. As shown in (Figure 4), rats subjected to NaNO2 showed a significant reduction in hemoglobin (Hb), RBC, packed cell volume (PCV), mean corpuscular volume (MCV), mean corpuscular hemoglobin (MCH), mean corpuscular hemoglobin concentration (MCHC), white blood cells (WBC), lymphocytes, platelets, and neutrophils, coupled with an increase in granulocytes and monocytes, compared to the control group. These alterations are indicative of altered hematopoiesis and immune function, likely due to adverse effects of NaNO2 on bone marrow, spleen, and liver, as well as oxidative damage and erythrocyte lysis induced by free radical production. On the contrary, the hematological analysis revealed that the administration of SCREE alone exhibits minimal effects on the examined hematological parameters, except for a notable increase in both red blood cell (RBC) and platelet counts at a high dose of 600 mg/kg BW, compared to the control group. Interestingly, the administration of SCREE to NaNO2-treated rats demonstrated, especially at a high dose of 600 mg/kg BW, a tendency toward normalized hematological parameters, approaching levels detected in the control group, which indicates a potential therapeutic effect of SCREE in mitigating NaNO-induced hematological disturbances (Figure 4). The observed changes in hematologic parameters reflect the general health and functioning of rats in response to SCREE administration. The alterations detected in parameters such as RBC count, Hb content, and WBC count indicate disturbances in oxygen transport, immune function, and overall physiological balance. The improvements noted by the SCREE administration suggest a potential protective and regulatory role.

These results are in agreement with previous reports that showed that 4 weeks of NaNO2 administration resulted in significant and dose-dependent reductions in RBC count, WBC count, and hemoglobin (Hb) content (Helal et al., 2008; Hammoud, 2014). These changes were associated with hypochromic microcytic anemia, likely due to the adverse effects of sodium nitrite on the bone marrow, spleen, and liver. The observed decrease in WBC count could also be attributed to insufficient white blood cell production in hematopoietic tissues (El-Sheikh and Khalil, 2011). Furthermore, sodium nitrite administration triggers free radical production, leading to the induction of oxidative damage and promoting the formation of methemoglobin and erythrocyte lysis through oxidation of ferrous ion oxyhemoglobin (Baky et al., 2010). The observed leukopenia was associated with lymphopenia, indicating the suppressive effect of sodium nitrite on the immune system (Gluhcheva et al., 2012). The noticeable decrease in WBCs in NaNO2-treated rats could be associated with the absence of fresh WBC generation from hematopoietic tissues (El-Sheikh and Khalil, 2011). On the other hand, the administration of SCREE extract to sodium nitrite treated rats improved the total leukocytic count. These findings could be attributed to the immunostimulatory activity of the SCREE extract. In this regard, Kadhem (abdul-hussein, 2019) showed that SCREE alleviates the toxic effect of paracetamol by improving hematological indicators (RBC count, WBC count, Hb, PCV, MCV, MCH and MCHC). The improved hematological markers observed could be attributed to L-proline, a significant metabolite within SCREE. L-proline exhibits various biological activities, including its potential role in boosting physiological functions such as improved collagen synthesis, wound healing, and neurotransmitter regulation (Ohtani et al., 2001; Albaugh et al., 2017). The substantial increase in red blood cell and platelet counts, particularly at the higher dose of SCREE (600 mg/kg BW), suggests that L-proline can certainly influence hematological parameters.

### 3.6 Assessment of the impact of SCREE on liver function

**FIGURE 5:**
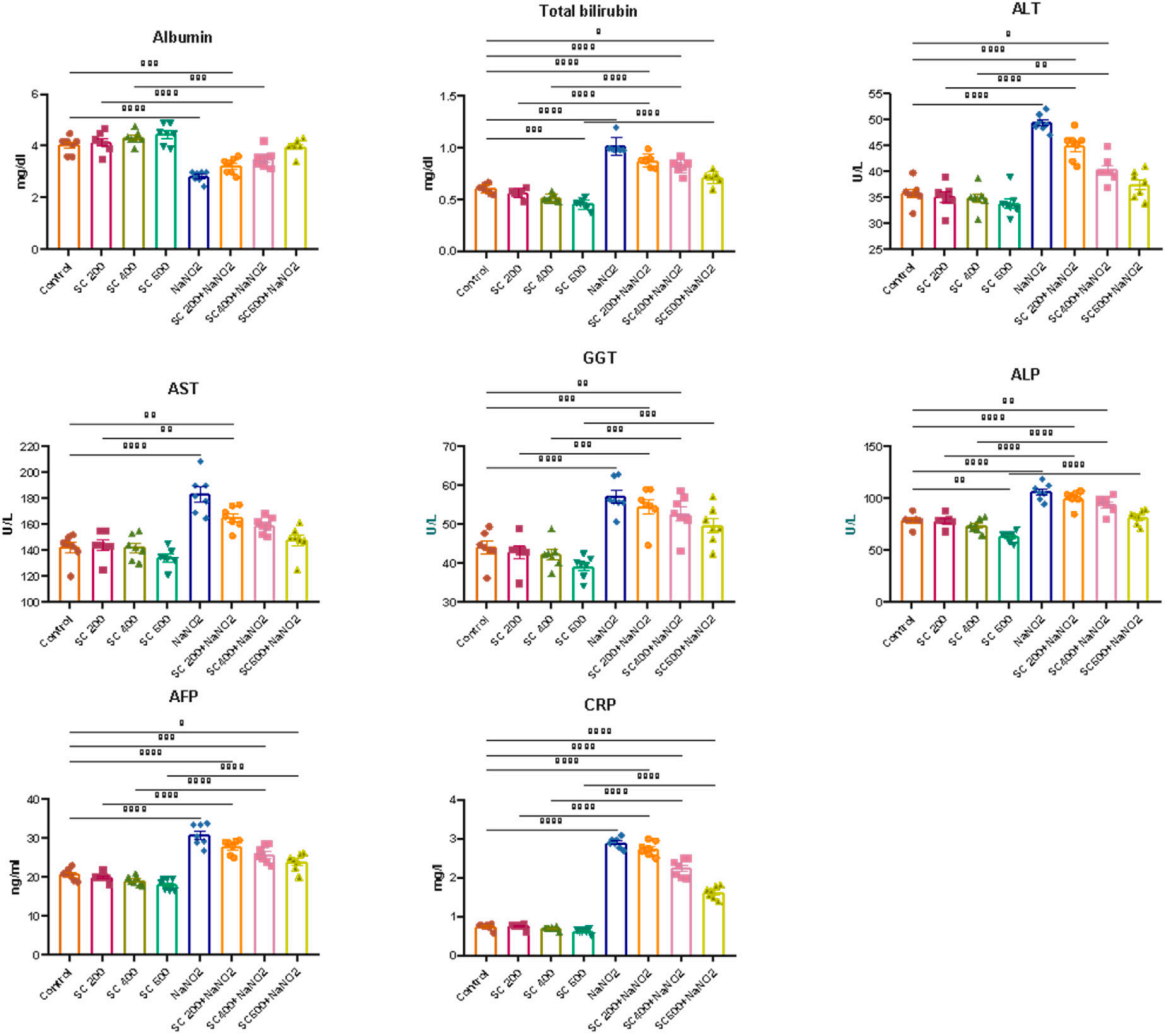
Effects of SCREE on liver function biomarkers (ALT, AST, ALP, GGT, total bilirubin, AFP, and CRP) of NaNO2-treated rats. Statistical analysis was reported as mean ± SE (n = 7). Multiple comparisons were analyzed by one-way ANOVA followed by Tukey’s multiple comparison test. Differences between groups were considered significant when *p* ≤ 0.05 (*p ≤ 0.05, **p ≤ 0.01, ***p ≤ 0.001, and * ***p < 0.0001). Significant data included for each group compared to the control group and for SCREE groups (SCREE 200, SCREE 400, SCREE 600) each compared to its respective treated group (SCREE 200+ NaNO2, SCREE 400+ NaNO2, SCREE 600+ NaNO2).

To evaluate the hepatoprotective effect of SCREE against NaNO2-induced liver damage, liver enzymes (ALT, AST, ALP and GGT), total bilirubin, AFP, and CRP activity were evaluated. As shown in (Figure 5), NaNO2-treated rats showed a substantial increase in serum ALT, AST, GGT, ALP, total bilirubin, AFP, and CRP activity, as well as a substantial drop in serum albumin, compared to the control group. Alterations in these markers can be attributed to hepatocellular inflammation and liver necrosis, which can lead to increased membrane permeability and subsequent release into the bloodstream (El-Demerdash et al., 2021a). Hepatocellular damage has also been associated with elevated total bilirubin levels caused by NaNO2 treatment. These results might reflect decreased liver conjugation, increased bilirubin generation by hemolysis, and decreased liver absorption (Le-Vinh et al., 2022). The notable toxic properties of nitroso derivatives, which form in the acidic environment of the stomach and cause severe liver necrosis, could be the cause of elevated liver enzyme activity (Chain et al., 2023). In addition, elevated AST and ALT activities in the NaNO2-treated group could be related to the nitric oxide-induced free radical (ONOO-) (Helal et al., 2020). Both oxygen radicals and NO possess the potential to react further to produce other oxidants and nitro substances, such as peroxynitrite, which can be harmful to the liver and contribute to liver cell death. The shift of the intracellular protein generation pathway may be responsible for the reduction of serum albumin levels, and the alteration of oxidative enzymes has a secondary impact on protein alterations. The observed elevation of AFP and CRP in NaNO2-treated rats was consistent with the findings of Tawfek et al. (Tawfek, 2015) and Elsherbiny et al. (Elsherbiny et al., 2017), who showed that certain food additives increase AFP and CRP levels in rats. In addition, Elsherbiny et al. (Elsherbiny et al., 2017) reported that NaNO2 administration increased inflammatory markers (CRP, TNF-α, IL-6, IL-1β) while reducing anti-inflammatory markers (IL-10 and IL-4). CRP is also an excellent sign of inflammation and immune dysfunction, as it has been linked to the development of arthritic disease in rats (Kadam and Bodhankar, 2013).

Next, we assessed the effect of SCREE supplementation alone on liver function. As depicted in (Figure 5), our results revealed that the administration of SCREE considerably improved the levels of certain biomarkers that are associated with hepatocellular injury. Interestingly, SCREE administration to the NaNO2-treated group demonstrated significant (*p* < 0.05) decreases (*p* < 0.05) in all parameters examined, except albumin, which increased dose-dependently. These findings suggest that SCREE may have a hepatoprotective effect by mitigating NaNO2-induced liver damage. The potential modulatory effect of SCREE is likely to be attributable to its antioxidant properties inherited by the presence of metabolites such as flavonoids, γ-Linolenic acid, D- (+) -Malic acid and chlorogenic acid, which are known to possess hepatoprotective properties via free radical-induced lipid peroxidation (Selvi et al., 2018). These findings are consistent with previous research that highlights the hepatoprotective potential of SCREE and its ability to modulate inflammatory and immunological responses (Tag et al., 2016b). Further, Elsayed et al. (Elsayed et al., 2015) showed that administration of SCREE root extract to rats treated with CCl4 improved plasma levels of ALT and AST.

### 3.7 Assessment of SCREE impact on kidney function and lipid profile

**FIGURE 6:**
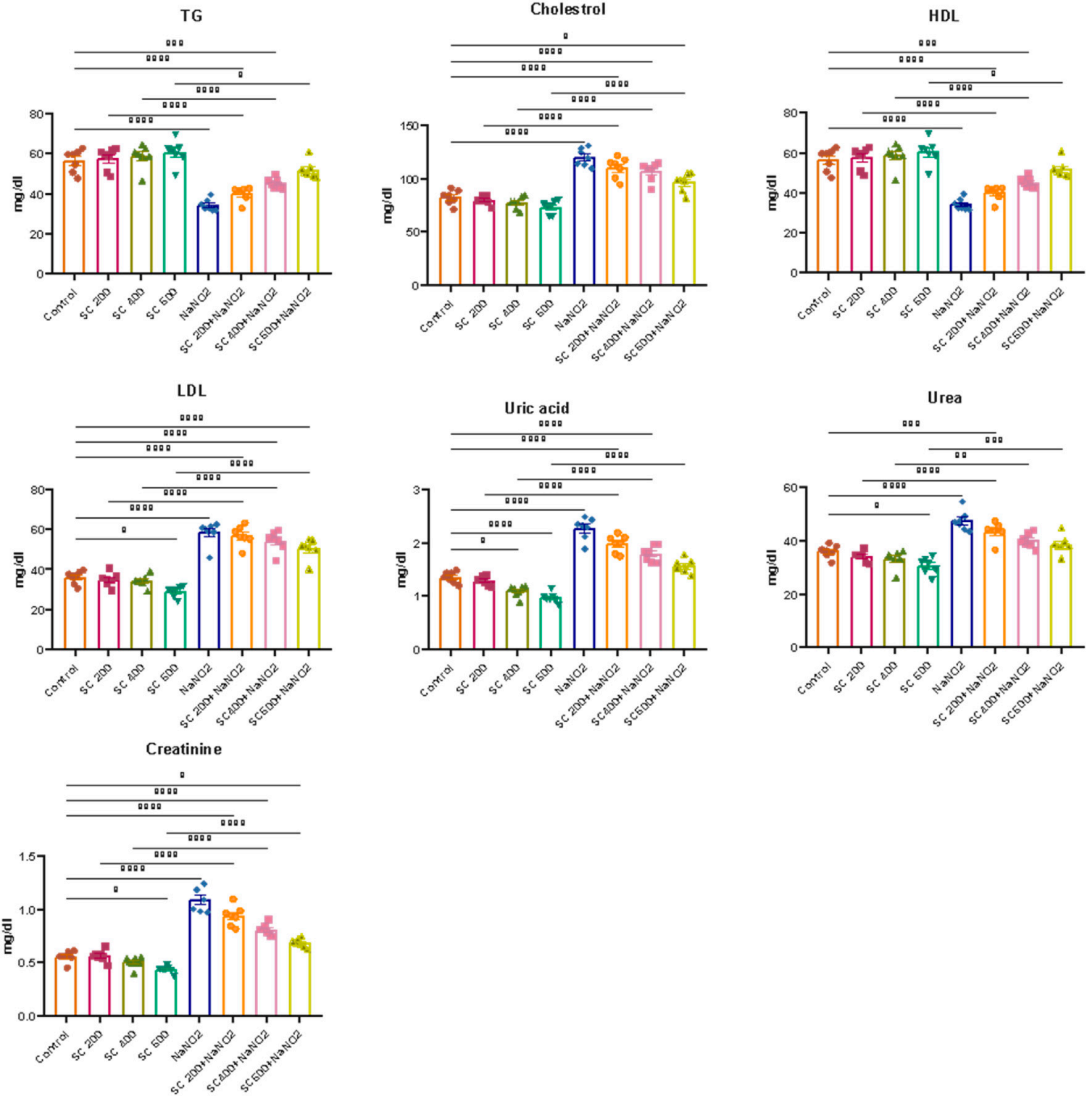
Effects of SCREE on kidney function biomarkers and lipid profile of NaNO2-treated rats. Statistical analysis was reported as mean ± SE (n = 7). Multiple comparisons were analyzed by one-way ANOVA followed by Tukey’s multiple comparison test. Differences between groups were considered significant when *p* ≤ 0.05 (*p ≤ 0.05, **p ≤ 0.01, ***p ≤ 0.001, and * ***p < 0.0001). Significant data included for each group compared to the control group and for SCREE groups (SCREE 200, SCREE 400, SCREE 600) each compared to its respective treated group (SCREE 200+ NaNO2, SCREE 400+ NaNO2, SCREE 600+ NaNO2).

We further explored the effect of SCREE on kidney function in NaNO2-treated rats by assessing the levels of certain kidney biomarkers and the lipid profile. Our results revealed that the administration of NaNO2 significantly increases urea, uric acid and creatinine levels, but also cholesterol, LDL-C, and VLDL-C levels. However, TG and HDL-C levels decreased significantly in NaNO2-treated rats, compared to the control group (Figure 6). These findings aligned with several reports that showed that NaNO2 administration increases urea and creatinine levels (Khalil, 2016; Helal et al., 2020). The observed elevation in markers of kidney function (urea, uric acid, and creatinine) suggests renal failure in NaNO2-treated rats (Johnson et al., 2013). On the contrary, high levels of creatinine are related to muscle creatinine catabolism and commonly signal acute kidney injury or chronic kidney disease (El-Demerdash et al., 2020). Most fatty acids in blood, tissues, and cellular membranes are unsaturated fatty acids and are particularly susceptible to ROS. Our results further highlight the nephrotoxic effect of sodium nitrite by triggering lipid peroxidation and oxidative stress that lead to kidney dysfunction (Aita and Mohammed, 2014). Elevated uric acid levels could potentially be attributed to increased uric acid use to counteract increased free radical production induced by NaNO2 treatment. Elevated urea levels observed in NaNO2-treated rats may indicate increased protein breakdown or impaired kidney function. Interestingly, the administration of SCREE to NaNO2-treated rats, particularly at a high dose of 600 mg/kg BW, resulted in decreased levels of uric acid and urea, suggesting that the administration of SCREE could regulate protein metabolism by inhibiting excessive protein breakdown or enhancing kidney function to facilitate efficient urea excretion. The observed changes in the lipid profile suggest an association with lipoprotein and lipid metabolism (El-Demerdash et al., 2021b). Our results revealed that the NaNO2-treated group exhibited elevated levels of cholesterol, TG, and LDL, along with decreased levels of HDL. However, significant improvements in blood lipid profiles were observed after SCREE treatment, characterized by reduced levels of cholesterol, TG and LDL, coupled with increased HDL levels. The elevated levels of lipid profiles observed after NaNO2 administration could potentially be attributed to the release of free fatty acids from adipose tissue into the circulation or the peroxidation of lipids from the cell membrane. These processes may lead to elevated levels of cholesterol production and acetyl CoA (Helal et al., 2000). However, SCREE alone administration demonstrated a beneficial effect on function and lipid profile, suggesting the potential of SCREE as a protective supplement. Consistent with these findings, SCREE administration to NaNO2-treated rats exhibited significant improvements in renal function and lipid profile in a dose-dependent manner (Figure 6). The observed nephroprotective effect of SCREE is consistent with the findings that reported the nephroprotective properties of S. costus extract against paracetamol-induced kidney damage, attributing this effect to the abundant presence of flavonoids and alkaloids (abdul-hussein, 2019). Our detailed metabolomic analysis of SCREE revealed a diverse array of metabolites, including flavonoids such as Apigenin and Luteolin, which possess considerable antioxidant and anti-inflammatory properties, suggesting their potential to mitigate kidney damage (Romanova et al., 2001; Wang et al., 2014a). Furthermore, γ-linolenic acid, a prominent metabolite detected in SCREE, exhibited recognized antioxidant properties and possible nephroprotective effects (Teng et al., 2017). The presence of D-(+)-malic acid, with its known hepatoprotective properties, further highlights the complexity of nephroprotection and the potential value of the SCREE extract (Koriem and Tharwat, 2023). Additionally, our findings indicate that SCREE treatment improved the lipid profile of the NaNO2-treated group. These results are in agreement with the findings of Alnahdi (2017), who demonstrated the beneficial effects of Costus extract in mitigating the adverse impacts of the pesticide deltamethrin on lipid profiles. The ability of SCREE to modify the lipid profile could be attributed to the presence of chlorogenic acid and Luteolin. Chlorogenic acid, a phenolic metabolite, has been extensively investigated for its potential to modulate lipid levels in the bloodstream by promoting fat metabolism leading to a reduction in total cholesterol and triglyceride levels (Murai and Matsuda, 2023). Luteolin, a flavonoid found in various plants, has also been explored for its lipid-modulating properties, potentially reducing total cholesterol and triglycerides while enhancing cholesterol levels that are beneficial for overall cardiovascular health (Muruganathan et al., 2022).

### 3.8 Evaluation of the anti-inflammatory and antiapoptotic potential of SCREE

**FIGURE 7:**
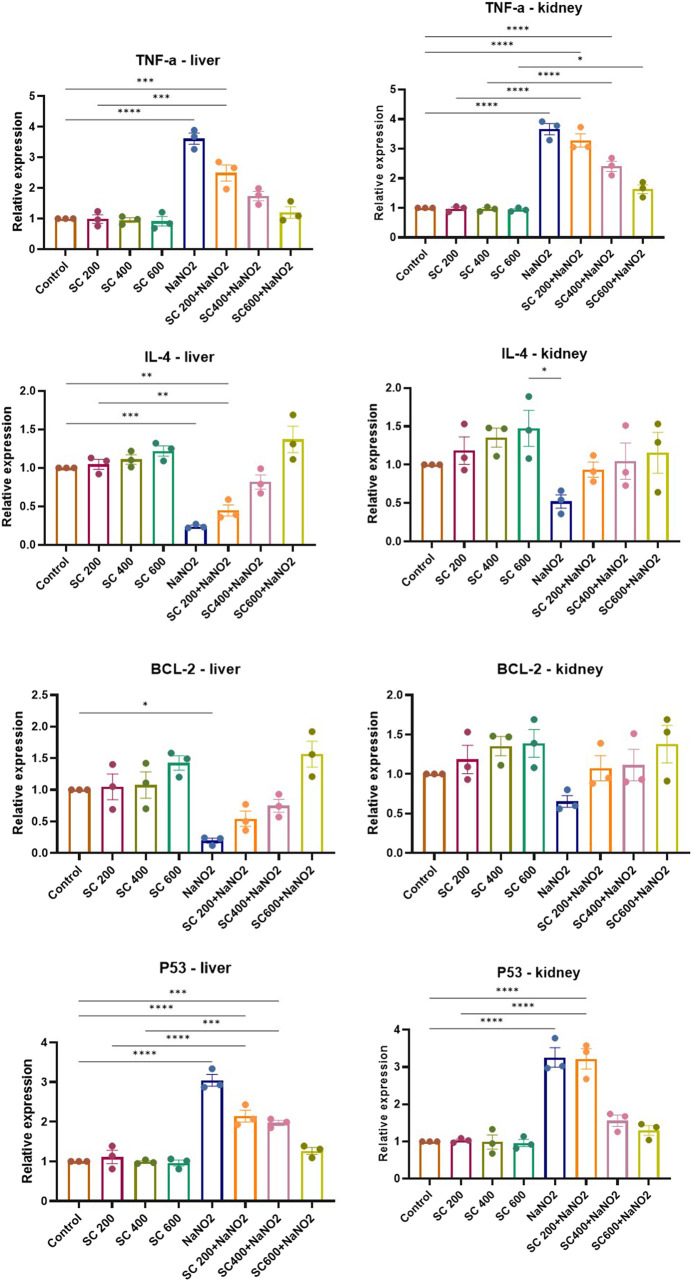
Effects of SCREE on the expression of inflammatory (TNF-α, IL-4) and apoptotic genes (P53, Bcl-2) in the liver and kidney of NaNO2-treated rats. Statistical analysis was reported as mean ± SE (n = 7). Multiple comparisons were analyzed by one-way ANOVA followed by Tukey’s multiple comparison test. Differences between groups were considered significant when *p* ≤ 0.05 (*p ≤ 0.05, **p ≤ 0.01, ***p ≤ 0.001, and ****p < 0.0001). Significant data included for each group compared to the control group and for SCREE groups (SCREE 200, SCREE 400, SCREE 600) each compared to its respective treated group (SCREE 200+ NaNO2, SCREE 400+ NaNO2, SCREE 600+ NaNO2).

To gain insight into the anti-inflammatory and anti-apoptotic impact of SCREE, we evaluated the gene expression of P53, Bcl2, IL-4, and TNF-α in the kidney and liver of control and NaNO2-treated rats. As shown in (Figure 7), our results revealed that administration of SCREE extract alone displays a nonsignificant effect on the expression of TNF-α and P53 genes in both the liver and the kidney, while increasing the expression of the IL-4 and Bcl2 genes, especially in the kidney. However, rats treated with sodium nitrite showed a considerable increase in the expression of TNF-α cytokine and tumor suppressor P53 gene in the kidney and liver, while a significant reduction was detected in the anti-inflammatory cytokine IL-4 and the apoptosis suppressor gene BCL-2, compared to the control group (Figure 7). These results are consistent with Soliman et al. (Soliman et al., 2022) and Elsherbiny et al. (Elsherbiny et al., 2017) who showed that administration increases the expression of TNF-α and other indicators related to inflammation (CRP, TNF-α, IL-6, IL-1β), but also reduces anti-inflammatory cytokine expression (IL-10 and IL-4). Moreover, Radwan et al. (Radwan et al., 2020) reported that the application of NaNO2 and benzoate mixture triggers alterations in immunohistopathology, biochemical markers, and p53 overexpression. Adu et al. (2020) and El-Nabarawy et al. (2020) showed that nitrite substantially influences the expression of levels of P53 and Bcl-2. Likewise, Soliman et al. (2022) showed that sodium nitrite-treated rats exhibit elevated levels of ROS, triggering numerous stress signaling pathways, including NF-B, which encourages increased expression of TNF-α, IL-1, and IL-6. Interestingly, the administration of SCREE demonstrated the ability to modulate the expression levels of inflammatory cytokines and apoptotic genes in the liver and kidney. In this regard, the administration of SCREE to NaNO2-treated rats exhibited a significant and dose-dependent ability to elevate the expression of the IL-4 and Bcl2 genes, but also downregulate the expression of TNF-α and tumor suppressor gene P53 in both kidney and liver (Figure 7). Our findings aligned with previous findings that revealed that the S. costus extract has the potential to regulate cell apoptosis by controlling the expression of the Bcl-2 and P53 genes (Alotaibi et al., 2021). Moreover, Zhao et al. (2008) found that sesquiterpenes from S. costus exhibit an anti-inflammatory ability to mitigate elevated levels of nitric oxide and TNF- release of TNF-α by LPS-activated macrophages. The observed anti-inflammatory and anti-apoptotic potential of SCREE could be associated with the detected set of metabolites, including apigenin, apigenin-7-O-glucoside, luteolin, daidzein, acacetin and formononetin. The improvement in apoptotic and inflammatory markers could be attributed to the presence of daidzein and apigenin in SCREE, which play a crucial role in the regulation of tumor cell invasion (Singh et al., 2023). Daidzein exerts its anti-inflammatory effects by downregulating TNF-α expression by inhibiting the nuclear factor-kappa B (NF-κB) signaling pathway. Daidzein also enhances IL-4 expression in experimental models by promoting Th2 cell differentiation (Wei et al., 2012). The anti-inflammatory properties of apigenin were observed in LPS-stimulated BV2 microglia, where activation of the GSK-3β/Nrf2 signaling pathway attenuated the expression of IL-6, IL-1β, and TNF-α (Chen et al., 2020). Together, our findings suggest that SCREE exhibits dual effects, comprising anti-inflammatory and anti-apoptotic activities.

## 4 Evaluation of differential display PCR (DD-PCR)

**FIGURE 8:**
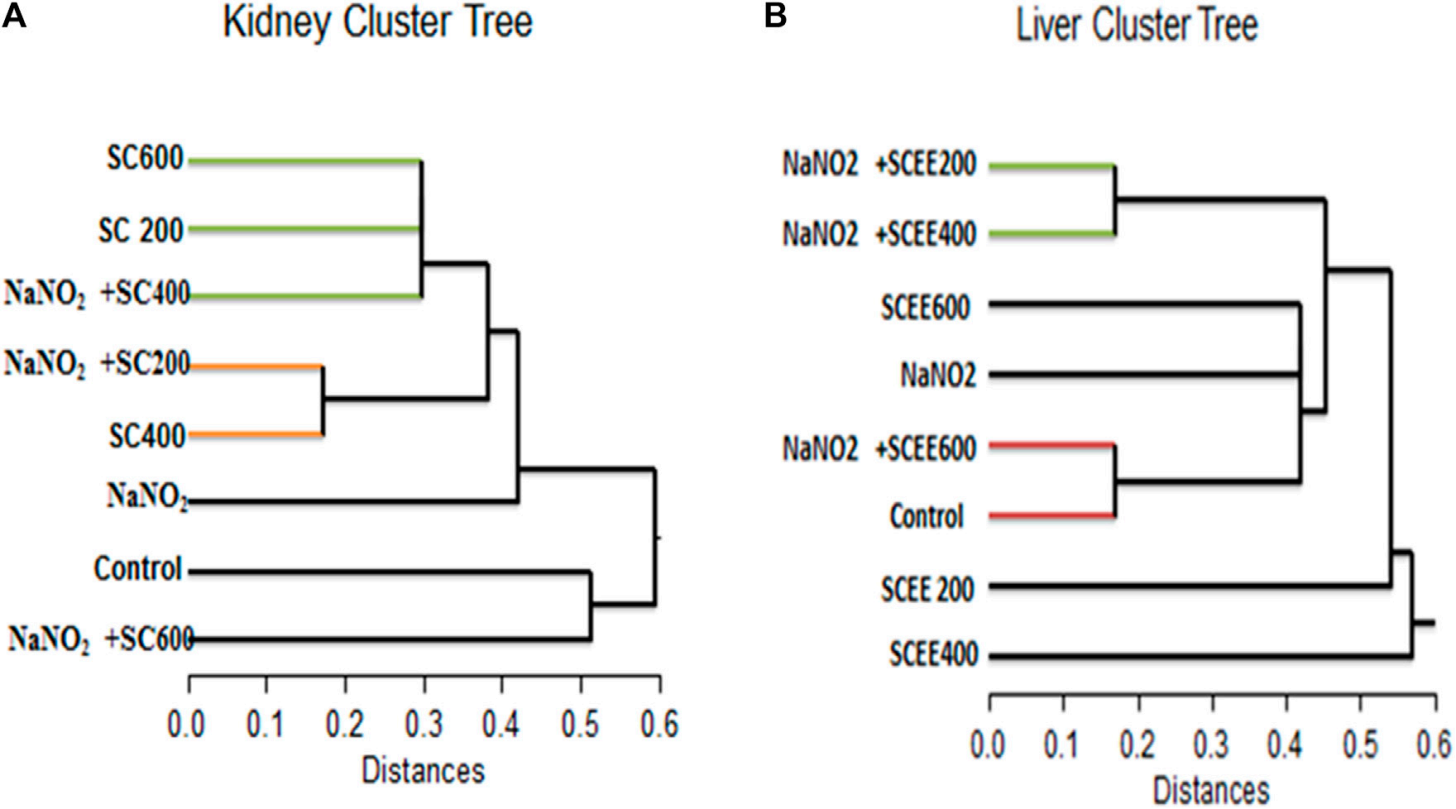
A dendrogram was created using the similarity index of the differentially expressed genes of the detected genes (regulated genes from the top) in the collected sera of the white albino rats examined in this study. **(A)** (on the left) for the kidney cluster tree, and **(B)** (on the right) for the liver cluster tree.

To gain more insight into the mode of action of SCREE, differential display PCR analysis was performed using the following primers (P5, P4, G23, G24, and NAR) for the kidney and liver. The total number of resolved bands for kidney samples in both control and treated samples was 4, 5, 9, 7, and nine bands for the P5, C24, G23, NAR and P4 primers, respectively. Furthermore, the total bands resolved for the liver for treatment and control samples were 4, 6, 9, 7, and nine bands for the P5, C24, G23, NAR and P4 primers, respectively. The typical number of bands per sample was 6.8 and seven for the kidney and liver, respectively. Of the 69 bands, 34 were for the kidney and 35 for the liver, six monomorphic (17.6%) and 28 (82.4%) polymorphic band recordings were recorded for the kidney, while 13 monomorphic (31.4%) and 24 (68.6%) polymorphic bands were recorded for the liver. Some common bands were seen in both treated samples and controls. There were few treatment-induced bands visible (the genes were turned on). On the contrary, some of the controls noticed bands that disappeared in the treated groups (genes were turned off). Numerous bands were highly lighted, indicating that these genes were overexpressed. Overall, these findings showed that numerous genes were found to be upregulated (overexpressed) and downregulated in various treatments when the chosen primers. Based on the differential display, a liver dendrogram was created that revealed that the eight treatment groups were divided by the tree into two main groups (Figure 8). Cluster one included the SCREE 400 (outer group), while Cluster two had the remaining seven treatments. There were two major subclusters in the second cluster; subcluster one contains SCREE 200, whereas the second subcluster was divided into two groups: the first group was divided into two subgroups, the first for NaNO2 and SCREE 200, the second subgroup for NaNO2 and SCREE 400. The second group was divided into three subgroups, the first subgroup for SCREE 600, the second for NaNO2, and the third subgroup included NaNO2 and SCREE 600 and the control group. In general, the tree topology shows that the NaNO2 + SCREE 600 group is more like the control group (Figure 8). However, the dendrogram constructed based on the differential kidney display indicated that the tree classified the eight treated groups into two main clusters. The first cluster was divided into two groups, the first for control and the second for NaNO2 + SCREE 600, and the remaining six treatments were part of the second cluster. Two subclusters were separated from the second cluster; the first was for the NaNO2 group NaNO2. In the second subgroup, there were two groups: the first group was divided into three subgroups, the first for SCREE 600, the second subgroup for SCREE 600, and the third subgroup for NaNO2 and SCREE 400. The second group was split into two subgroups, the first for NaNO2 and SCREE 200 and the second for SCREE 400. The phylogeny tree has revealed that NaNO2 + SCREE 600 is closer to the control group (Figure 8). Animals given NaNO2 + SCREE 600 were found to have the main genetic profile obtained with the control, which means that SCREE 600 removes any effects on NaNO2, which is observed from the behavior of the mRNA profile. These findings align with the results of Fadda et al. (2018b), who observed that a select few antioxidants were effective in modulating the expression of NF-jB, BcL-2, Bax and flt-1 mRNA. Furthermore, compared to rats exposed to NaNO2-induced toxicity, these antioxidants also exhibited the ability to regulate factors such as oxidative DNA damage, vascular endothelial growth factor (VEGF), and the apoptotic marker caspase 3. The observed enhancement associated with SCREE aligns with the research by Chen et al. (1995), which revealed that the active metabolites within SCREE, namely, costunolide and dihydrocostus lactone, effectively inhibit the expression of the hepatitis B virus surface antigen gene in human hepatic tissue. In addition, Bains et al. (2019) found that the presence of sesquiterpene lactones gives SCREE its enormous pharmacological potential and molecular efficiency. On the other hand, Fukuda et al. (2001) found that the main sesquiterpene lactone in SCREE, costunolide, has chemoprotective effects on the development of cancer. Further, Kang et al. (2004) found that the biological activity of sesquiterpene lactone extracted from the root of S. costus includes anticarcinogenic and antifungal effects.

### 4.1 Histopathological analysis of the liver

**FIGURE 9:**
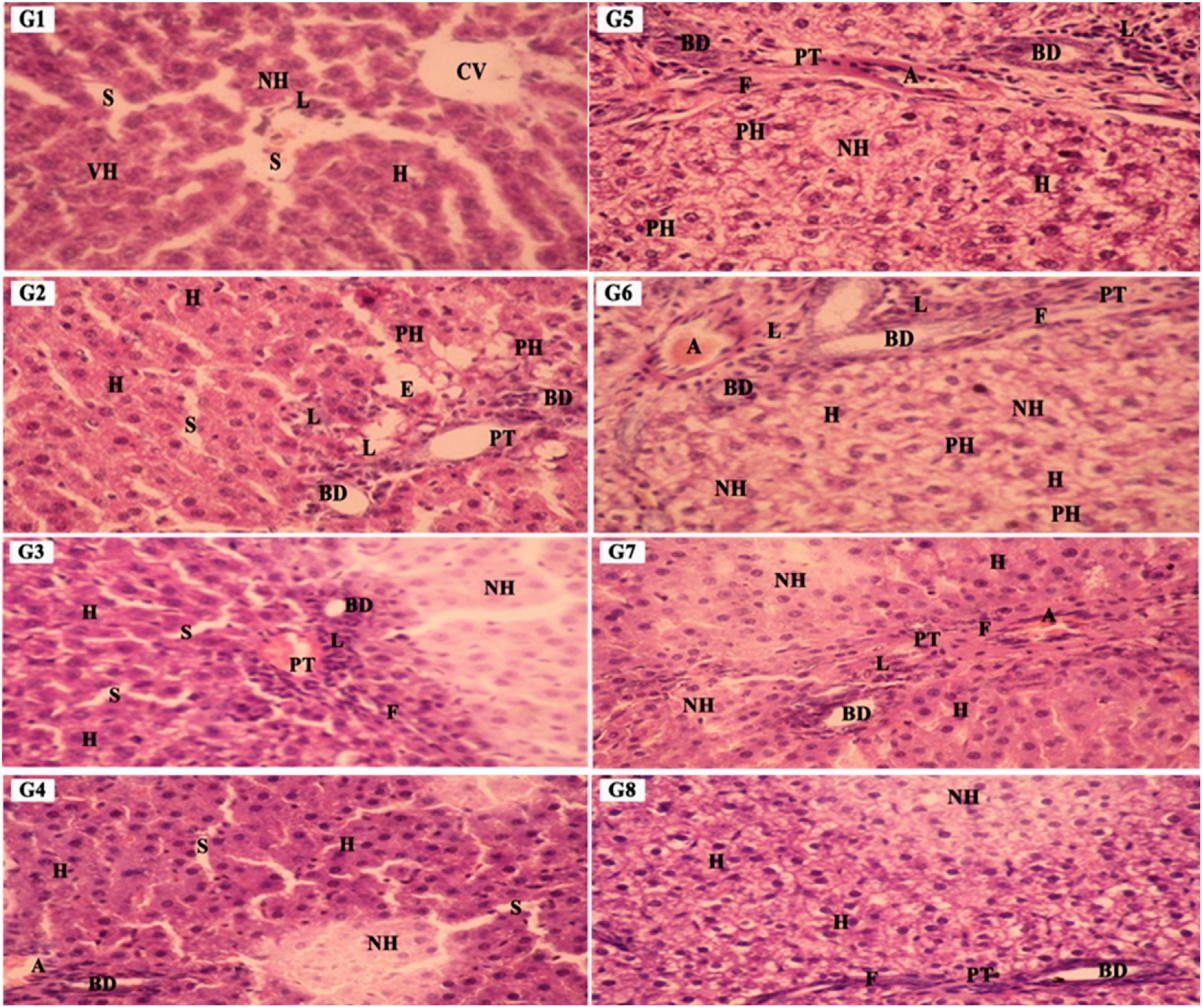
The liver sections’ photomicrographs in various groups, G1: Control G2: SCREE (200 mg/kg BW), G3: SCREE (400 mg/kg BW), G4: SCREE (600 mg/kg BW), G5: NaNO2 G6: SCREE (200 mg/kg BW) + NaNO2, G7: SCREE (400 mg/kg BW) + NaNO2 and G8: SCREE (600 mg/kg BW) + NaNO2, EH: eosinophilic hepatocytes, CV: central vein, PT: portal tract, H: Hepatocyte, AM: mitotic hepatocytes, BD: bile duct, L: lymphocyte, NH: necrotic hepatocyte, F: fibrotic cells, N: necrotic cells, S: sinusoid, VH: vesiculated nuclei, IF: infiltrating lymphocytes, PH: pyknotic hepatocytes. (H&E stain, ×400magn.)

We further expand our study to explore the effect of SCREE by performing a detailed histopathological analysis of the liver to validate the results and identify any pathological changes. As shown in (Figure 9), histological examination of liver segments stained with hematoxylin and eosin in the control (G1) and SCREE (G2, G3 and G4) rat groups revealed a normal liver architecture compared to the control (Table 2). However, liver sections of NaNO2-treated rats (G5) showed marked dilation of the portal tract and bile duct surrounded by marked fibrotic cells and necrotic cells. According to our biochemical findings, liver sections from rats coadministered with SCREE and NaNO2 (G6, G7, and G8) exhibited a significant modulation of the toxic action of NaNO2, and regenerating hepatocytes were seen especially with the high dose of SCREE compared to the NaNO2 group (Figure 9). In our study, liver sections from rats co-administrated with SCREE and NaNO2 showed a noticeable reduction in the harmful effects of NaNO2. Furthermore, considerable regenerating hepatocytes were observed in the group that received a high dose of SCREE, compared to the NaNO2 group. Several authors reported that in the NaNO2-treated group significant histological changes were observed, indicating hepatotoxicity (Aita and Mohammed, 2014). In this regard, Fouad et al. (Khalil, 2016) showed that NaNO2 toxicity caused degenerative changes, including vacuolar degeneration of hepatocytes, as well as congestion and swelling of the blood sinusoid and portal vein. The potential hepatoprotective and antifibrotic effects of S. costus root may be due to its ability to block the calcium channel that prevents hypoxia and new angiogenesis (Gilani et al., 2007; Ali et al., 2018). Furthermore, it was shown that liver histological structure was significantly improved by the ethanol extract of S. costus (Elshaer et al., 2022). Together, our findings further indicate that SCREE has the ability to decrease the effect of NaNO2 deterioration on liver tissue.

**TABLE 2:** Scores of liver and kidney histological changes in various groups.

Group	parameters	GP1	GP2	GP3	GP4	GP5	GP6	GP7	GP8
Liver									
CV dil	Score	1.01 ± 0.03	0 ± 0.0	0 ± 0.0	0 ± 0.0	0 ± .0	0 ± 0.0	0 ± 0.0	0 ± 0.0
	Change (%)	100	0	0	0	0	0	0	0
PT dil	Score	0 ± 0.0	2.21 ± 0.04	2.04 ± 0.02	0 ± 0.0	3.45 ± 0.06	2.32 ± 0.01	2.23 ± 0.0	2.03 ± 0.01
	Change (%)	0	221	204	0	0	232	223	203
B D dil	Score	0 ± 0.0	2.17 ± 0.03	2.12 ± 0.05	0 ± 0.0	3.48 ± 0.04	3.05 ± 0.03	2.23 ± 0.0	0 ± 0.0
	Change (%)	0	217	212	0	348	305	223	0
Fibrosis	Score	0 ± 0.0	0 ± 0.0	1.02 ± 0.01	1.00 ± 0.01	3.39 ± 0.06	3.23 ± 0.0	2.40 ± 0.09	1.20 ± 0.0
	Change (%)	0	100	102	10.0	33.9	32.3	24.0	12.0
Necrosis	Score	1.16 ± 0.02	0 ± 0.0	1.01 ± 0.01	0 ± 0.0	2.35 ± 0.02	2.23 ± 0.06	0 ± 0.0	0 ± 0.0
	Change (%)	110	0	87.1	0	232.7	220.8	0	0
Regenerative H	Score	0 ± 0.0	2.09 ± 0.02	2.01 ± 0.01	2.02 ± 0.01	0 ± 0.0	0 ± 0.0	0 ± 0.0	2.02 ± 0.01
	Change (%)	0	209	201	202	0	0	0	202
Kidney									
Glomeruliatrophy	Score	0 ± 0.0	2.03 ± 0.01	2.07 ± 0.01	0 ± 0.0	4.29 ± 0.06	2.27 ± 0.06	2.18 ± 0.02	1.18 ± 0.05
	Change (%)	0	203	207	0	429	227	218	118
RT dil	Score	2.06 ± 0.02	3.27 ± 0.02	3.12 ± 0.03	3.03 ± 0.02	4.41 ± 0.1	4.19 ± 0.02	4.05 ± 0.02	3.12 ± 0.03
	Change (%)	100	158.7	151.5	147.1	214.1	203.4	196.6	151.5
Regenerative R.T	Score	0 ± 0.0	0 ± 0.0	2.13 ± 0.05	2.05 ± 0.02	0 ± 0.0	0 ± 0.0	1.24 ± 0.02	2.29 ± 0.06
	Change (%)	0	0	213	205	0	0	124	229

The percentage change was assessed relative to the control group (G1). Data presented as mean ± SE, values.

### 4.2 Kidney histopathological analysis

**FIGURE 10:**
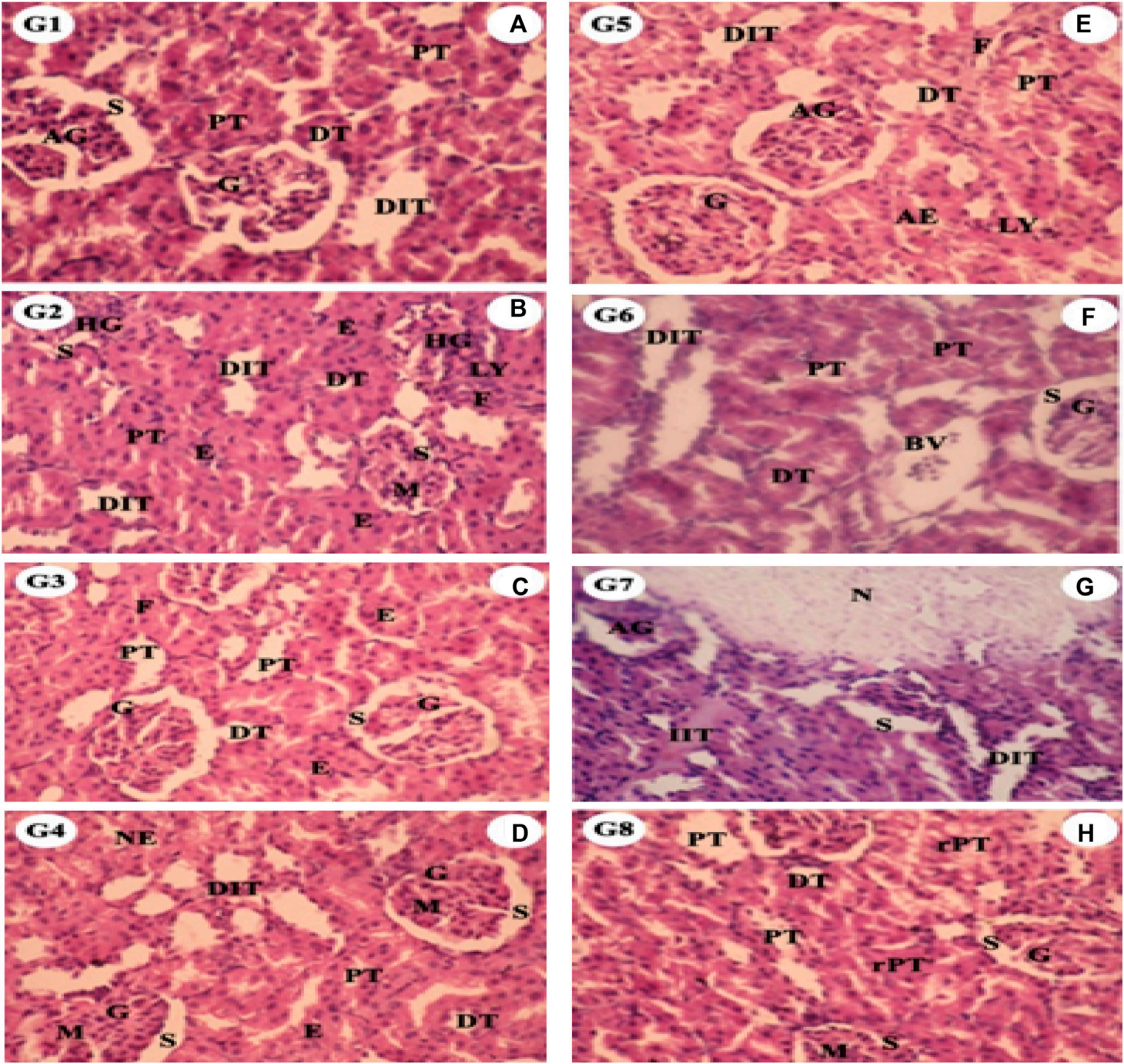
The kidney sections’ photomicrographs in various groups G1: Control G2: SCREE (200 mg/kg BW), G3: SCREE (400 mg/kg BW), G4: SCREE (high dose), G5: NaNO2 G6: SCREE (200 mg/kg BW) + NaNO2, G7: SCREE (400 mg/kg BW) + NaNO2 and G8: SCREE (600 mg/kg BW)+ NaNO2, G: normal glomeruli, M: mesangial cells, PT: proximal tubule, S: minimal urinary space, IT: interstitial tissue, HG: hyperemic, DT: distal tubule, AG: atrophied glomeruli, (H&E stain, ×400magn.).

Next, we evaluated kidney tissues stained with hematoxylin and eosin to examine the effect of SCREE on mitigating the effect of NaNO2 treatment. As shown in Figure 10, the renal cortex of the kidney tissue of control (G1) and SCREE (G2, G3 and G4) showed a normal histological architecture of the glomeruli and renal tubules under light microscopy. Unlike the control group, microscopic examination of renal cortex sections of the NaNO2 group (G5) revealed marked atrophied of many glomeruli (AG) surrounded by marked dilation renal tubules (DIT), marked dilation of proximal and distal tubules with many atrophied epithelial cells (AE), and differentiated fibrotic cell (F) (Figure 10). Compared to the group that received sodium nitrite, co-administration of SCREE with NaNO2 (G6, G7, and G8) altered the toxic action of NaNO2, and regeneration of renal tubules was observed at medium and high doses. Similarly, microscopic examination of the renal tubules revealed a wide variety of necrobiotic alterations, including vacuolization, swelling, and necrosis of the epithelium that encloses the convoluted proximal tubules. These findings are consistent with previous research that demonstrated the nephrotoxic potential of NaNO2 administration by inducing notable kidney effects, particularly tubular degeneration, in conjunction with concurrent hydropic cellular degeneration in the liver. These findings emphasize the significant nephrotoxic potential of sodium nitrite, suggesting a direct detrimental impact on renal tissue integrity and function in rodent models (Özen et al., 2014).

In the current study, co-administration of SCREE altered the toxic effects of NaNO2 and improved the regeneration of renal tubules at medium and high doses, compared to the NaNO2-treated group. These changes are ascribed to NaNO2-inducing hypoxia, leading to the creation of free radicals that cause tissue damage (Aita and Mohammed, 2014). These findings aligned with Ayaz who found that early oral administration of S. costus extract (300 mg/kg BW) for 28 days may protect renal tissue from oxidative stress caused by deltamethrin’s harmful effects (Ayaz, 2017). The enhanced architecture of the kidney tissue observed with SCREE administration could be attributed to the presence of metabolites such as apigenin and its derivative, apigenin-7-O-glucoside, which exhibit potent antioxidant properties through multiple mechanisms. They interact directly with radicals and metals, leading to a stop in the chain reaction of oxidative stress. Furthermore, apigenin and its glucoside derivative mitigate lipid peroxidation, thus preserving cell membrane integrity (Kashyap et al., 2022). Collectively, these findings further support the therapeutic potential of SCREE to diminish the deterioration effect of NaNO2 treatment on the histological architecture and kidney function.

### 4.3 Liver immunohistochemical analysis

**FIGURE 11:**
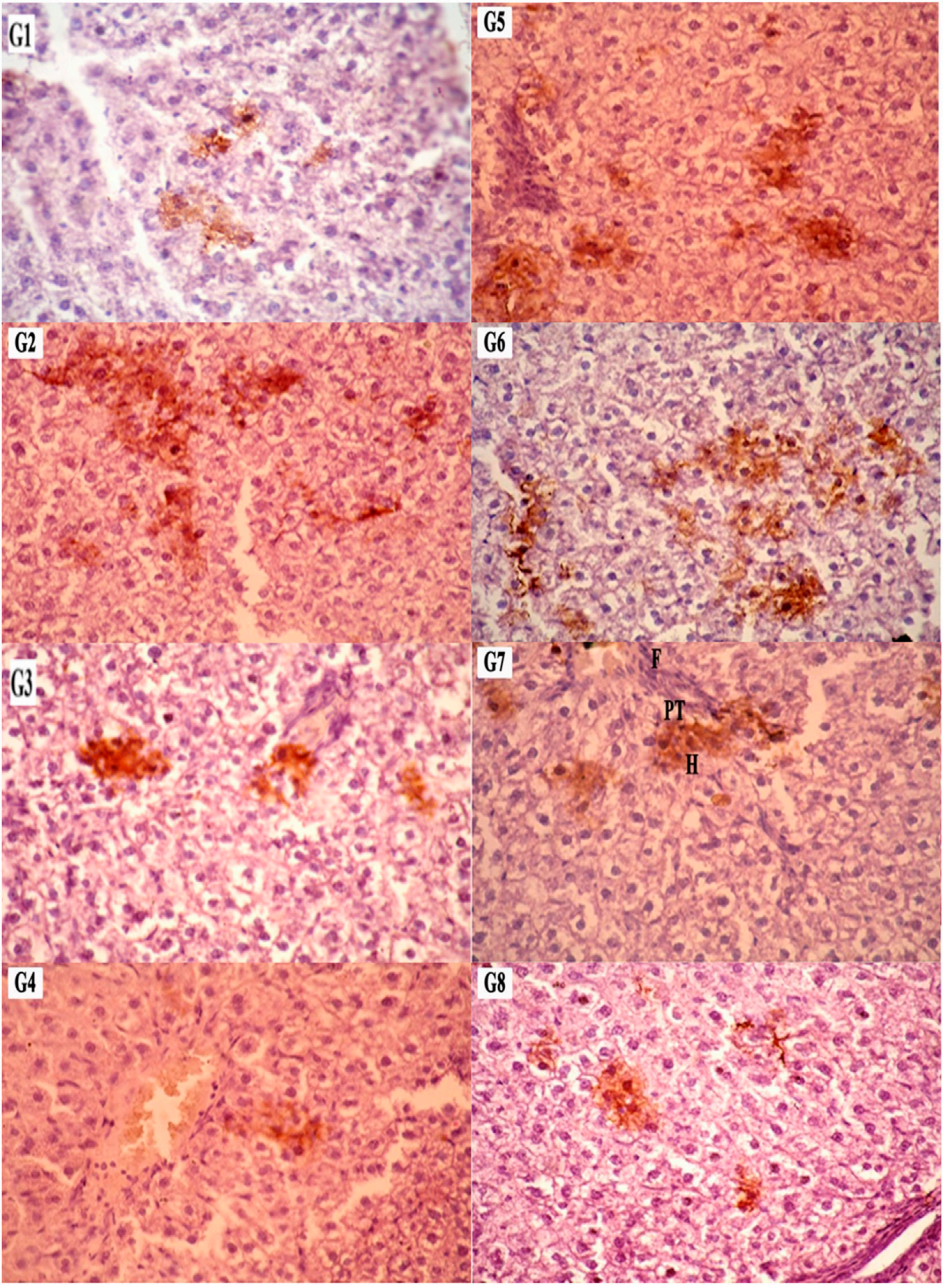
Photomicrographs of TGF-β immunoreactivity expression as brownish color in rat liver sections in different groups. G1: Control G2: SCREE (200 mg/kg BW), G3: SCREE (400 mg/kg BW), G4: SCREE (high dose), G5: NaNO2, G6: SCREE (200 mg/kg BW) + NaNO2, G7: SCREE (400 mg/kg BW) + NaNO2, and G8: SCREE (600 mg/kg BW) + NaNO2, PT: portal tract, H: hepatocyte, F: fibrotic cells (DAB 400x stain).

Finally, we performed immunohistochemical analysis to assess the distribution of immunostaining expression of the transforming growth factor β protein (TGF- β) in liver tissues. As shown in Figure 11, TGF-ß was shown as a brown color diffuse in the cell membrane and cytoplasm of hepatocytes. The liver sections of the control group (G1) showed weak expression for TGF-β. The control SCREE group (G2, 200 mg/kg BW) showed moderately positive expression (+2) of TGF-β protein in the cytoplasm of the centrilobular area of the hepatocytes (CA), while the SCREE group (G3, 400 mg/kg BW) also showed moderately positive expression (+2) in the cytoplasm of the hepatocytes and the cell membrane in the portal area (PT) surrounded by few fibrotic cells (Table 3). Interestingly, the SCREE group (G4, 600 mg/kg BW) exhibited a mild positive expression (+1) in the cytoplasm of hepatocytes and the cell membrane in the portal area (PT) lack fibrotic cells and the area of regenerative hepatocytes. On the contrary, the liver segments of the NaNO2-treated group (G5) exhibited a strongly positive reaction (+4) of TGF-β protein in the cytoplasm of hepatocytes in the centrilobular area (CA), moderate positive expression (+2) in the cytoplasm of the hepatocytes cytoplasm and cell membrane in the portal area (PT) and the cell membrane mostly adjacent to the necrotic area of hepatocytes. Furthermore, the liver segments of group (G6) showed strongly positive expression (+4) of TGF-β protein in the cytoplasm of the hepatocytes and the spread of the cell membrane in the centrilobular area (CA), while group (G7) showed moderate positive expression (+2) in the cytoplasm of the hepatocytes and the cell membrane in the portal area (PT) with few fibrotic cells (F). Finally, group (G8) showed moderate positive expression (+2) in the cytoplasm and cell membranes of the few hepatocytes in the fibrotic area of the portal tract (PT) (Table 3). The high expression of TGF-β in liver tissue after NaNO2 treatment could also be attributed to hepatic tissue damage caused by changes in liver function markers. Similarly, AlRasheed et al. found that NaNO2 administration resulted in a highly significant increase in small mothers against expressions of decapentaplegic homlog 2(Smad-2), serine/threonine protein kinase (AKT), and hypoxia-inducible factor 1 alpha (HIF1-) with a simultaneous reduction in BcL-2 expression when compared to control in the hepatic, pulmonary, renal, and cardiac tissues (Al-Rasheed et al., 2017). Furthermore, Sherif et al. reported a significant increase in monocyte chemoattractant protein-1 (MCP-1) and TGF-1 levels in the liver of sodium nitrite treated rats (Imam and Mohammed, 2013). Soliman et al. found that NaNO2 treatment elevated ROS production and activated numerous stress signaling pathways, including TNF-α, and TGF-β (Soliman et al., 2022). The observed improvement in SCREE treated groups is consistent with Jia et al. (2013) who found that sesquiterpene lactones prevent MCP-1/TGF-β pathway and the activation of the nuclear factor kappa B (NF-kB) induced by high glucose in rat mesangial cells. Our results indicate that SCREE has the potential to modulate the expression of TGF-β protein in liver tissue and the ability to mitigate the deterioration effect of NaNO2 treatment.

**TABLE 3:** Scores of immunohistochemical observations of TGF- β in the liver in various groups.

Groups	Expression of TGF- β				Notes
	CA		PT		
	Score	Change (%)	Score	Change (%)	
Gp1	0 ± 0.0	0	1.05 ± 0.02	100	-
Gp2	4.25 ± 0.03	425	0 ± 0.0	0	-
Gp 3	0 ± 0.0	0	2.12 ± 0.03	201.9	few fibrotic cells
Gp 4	0 ± 0.0	0	1.03 ± 0.01	98.1	regenerative hepatocytes
Gp 5	4.29 ± 0.06	429	2.43 ± 0.04	231.4	-
Gp 6	4.04 ± 0.01	404	0 ± 0.0	0	-
Gp 7	0 ± 0.0	0	2.11 ± 0.01	201.0	Few fibrotic cells
Gp 8	0 ± 0.0	0	2.02 ± 0.01	192.4	-

The percentage change was assessed relative to the control group (G1),. Data presented as mean ± SE, values.

## 5 Conclusion

This study sheds light on the critical health implications associated with the use of sodium nitrite as a food additive, particularly on vital organs such as the liver and kidneys. Our presented study explored the potential of SCREE supplementation to mitigate NaNO2-induced toxicity. Administration of SCREE demonstrated a remarkable ability to counteract the toxic effects of NaNO2 in a dose-dependent manner. This improved effect was evident in improvements in hematological parameters, lipid profile, and modulation of histopathological architecture in the liver and kidneys. Furthermore, SCREE exhibited a regulatory effect on TNF-α, P53, IL-4, and BCL-2 markers, suggesting its potential to modulate inflammatory and apoptotic pathways. The comprehensive phytochemical analysis of SCREE identified a diverse array of primary and secondary metabolites, including phenolics, flavonoids, vitamins, alkaloids, saponins, and tannins. This unique phytochemical profile, coupled with the observed therapeutic effects, positions SCREE as a promising natural food detoxifying additive. Taken together, this study highlights the potential of the ethanolic extract of the Saussurea costus root as a valuable natural intervention to mitigate the detrimental effects of sodium nitrite, offering a basis for further exploration and development of SCREE as a safe and effective food detoxification strategy in the realm of human health nutrition.

## Data Availability

The raw data supporting the conclusion of this article will be made available by the authors, without undue reservation.

## References

[B1] Abdallah E. M. Qureshi K. A. Ali A. M. Elhassan G. (2017). Evaluation of some biological properties of Saussurea costus crude root extract. Biosci. Biotech. Res. Comm. 10 (4), 601–611. 10.21786/bbrc/10.4/2

[B2] Abd El-Rahman G. I. Behairy A. Elseddawy N. M. Batiha G. E.-S. Hozzein W. N. Khodeer D. M. (2020). Saussurea lappa ethanolic extract attenuates triamcinolone acetonide-induced pulmonary and splenic tissue damage in rats via modulation of oxidative stress, inflammation, and apoptosis. Inflamm. apoptosis 9 (5), 396. 10.3390/antiox9050396 32397156 PMC7278611

[B3] abdul-hussein M. (2019). Protective of ethanolic extract of Saussurea lappa against paracetamol-induced hepatic and renal damage in male rabbits. Asian J. Pharm. Clin. Res. 12, 68–73. 10.22159/ajpcr.2019.v12i18.34218

[B4] Abo-El-Sooud K. Hashem M. M. Abd ElHakim Y. M. Kamel G. M. Eleiwa M. Gab-Alllah A. (2019). Effect of sodium nitrite exposure on the immune responses against of rift valley fever vaccine in mice. Int. J. Pharm. Pharm. Sci., 28–31. 10.22159/ijpps.2019v11i7.33443

[B5] Adu A. Sudiana I. Martini S. (2020). The effect of nitrite food preservatives added to se’i meat on the expression of wild-type p53 protein. Open Chem. 18, 559–564. 10.1515/chem-2020-0094

[B6] Ahmed A. H. A. (2017). The effect of water extracts of Phyllanthus emblica and Costus speciousus on reducing obesity in albino rats. Alexandria Sci. Exch. J. 38, 463–472. 10.21608/asejaiqjsae.2017.3750

[B7] Aita N. Mohammed F. (2014). Effect of marjoram oil on the clinicopathological, cytogenetic and histopathological alterations induced by sodium nitrite toxicity in rats. Glob. Veterinaria 12, 606–616. 10.5829/idosi.gv.2014.12.05.83186

[B8] Albaugh V. L. Mukherjee K. Barbul A. (2017). Proline precursors and collagen synthesis: biochemical challenges of nutrient supplementation and wound healing. J. Nutr. 147 (11), 2011–2017. 10.3945/jn.117.256404 28978679 PMC5657141

[B9] Al-Gayyar M. M. Alyoussef A. Hamdan A. M. Abbas A. Darweish M. M. El-Hawwary A. A. (2015). Cod liver oil ameliorates sodium nitrite-induced insulin resistance and degradation of rat hepatic glycogen through inhibition of cAMP/PKA pathway. Life Sci. 120, 13–21. 10.1016/j.lfs.2014.11.002 25447450

[B10] Ali M. Khan T. Fatima K. Ali Q. u. A. Ovais M. Khalil A. T. (2018). Selected hepatoprotective herbal medicines: evidence from ethnomedicinal applications, animal models, and possible mechanism of actions. Phytotherapy Res. 32 (2), 199–215. 10.1002/ptr.5957 29047177 PMC7167792

[B11] Alnahdi H. S. (2017). Injury in metabolic gland induced by pyrethroid insecticide could Be reduced by aqueous extract of sassura lappa. Int. J. Pharm. Res. Allied Sci. 6 (2), 86–97.

[B12] Alotaibi A. A. Bepari A. Assiri R. A. Niazi S. K. Nayaka S. Rudrappa M. (2021). Saussurea lappa exhibits anti-oncogenic effect in hepatocellular carcinoma, HepG2 cancer cell line by bcl-2 mediated apoptotic pathway and mitochondrial cytochrome C release. Curr. Issues Mol. Biol. 43, 1114–1132. 10.3390/cimb43020079 34563048 PMC8929068

[B13] Al-Rasheed N. M. Fadda L. M. Attia H. A. Ali H. M. Al-Rasheed N. M. (2017). Quercetin inhibits sodium nitrite-induced inflammation and apoptosis in different rats organs by suppressing Bax, HIF1-α, TGF-β, Smad-2, and AKT pathways. J. Biochem. Mol. Toxicol. 31 (5), e21883. 10.1002/jbt.21883 28000380

[B14] Al-Zayadi Z. A. Shanan H. K. Al Salihi K. A. (2023). “Extraction and evaluation of active ingredients of Saussurea costus roots and determination of its antibacterial activity,” in IOP conference series: earth and environmental science (Bristol, United Kingdom: IOP Publishing).012058

[B15] Armeni T. Cianfruglia L. Piva F. Urbanelli L. Caniglia M. L. Pugnaloni A. (2014). S-D-Lactoylglutathione can be an alternative supply of mitochondrial glutathione. Free Radic. Biol. Med. 67, 451–459. 10.1016/j.freeradbiomed.2013.12.005 24333633

[B16] Arrivault S. Guenther M. Ivakov A. Feil R. Vosloh D. Van Dongen J. T. (2009). Use of reverse-phase liquid chromatography, linked to tandem mass spectrometry, to profile the Calvin cycle and other metabolic intermediates in Arabidopsis rosettes at different carbon dioxide concentrations. Plant J. 59 (5), 826–839. 10.1111/j.1365-313X.2009.03902.x 19453453

[B17] Asif M. J. C. (2015). Chemistry and antioxidant activity of plants containing some phenolic compounds. Biomolecules 1 (1), 35–52. 10.31221/osf.io/rygwm

[B18] Attallah N. G. M. Kabbash A. Negm W. A. Elekhnawy E. Binsuwaidan R. Al-Fakhrany O. M. (2023). Protective potential of Saussurea costus (falc.) Lipsch. Roots against cyclophosphamide-induced pulmonary injury in rats and its *in vitro* antiviral effect. Pharmaceuticals 16 (2), 318. 10.3390/ph16020318 37259460 PMC9959296

[B19] Ayaz N. O. (2017). Modulating impacts of coustus Sassura lappa extract against oxidative stress and genotoxicity induced by deltamethrin toxicity in rat kidneys. Int. J. Pharm. Res. allied Sci. 6 (2), 49–60.

[B20] Bains S. Thakur V. Kaur J. Singh K. Kaur R. (2019). Elucidating genes involved in sesquiterpenoid and flavonoid biosynthetic pathways in Saussurea lappa by *de novo* leaf transcriptome analysis. Genomics 111 (6), 1474–1482. 10.1016/j.ygeno.2018.09.022 30343181

[B21] Baky N. A. A. Zaidi Z. F. Fatani A. J. Sayed-Ahmed M. M. Yaqub H. (2010). Nitric oxide pros and cons: the role of l-arginine, a nitric oxide precursor, and idebenone, a coenzyme-Q analogue in ameliorating cerebral hypoxia in rat. Brain Res. Bull. 83 (1), 49–56. 10.1016/j.brainresbull.2010.07.004 20637840

[B22] Bancroft J. D. Stevens A. Bancroft J. D. Stevens A. (1990) Theory and practice of histological techniques.

[B23] Bari M. L. Yeasmin S. (2018). “Foodborne diseases and responsible agents,” in Food safety and preservation (Amsterdam, Netherlands: Elsevier), 195–229.

[B24] Barrero A. F. Oltra J. E. Álvarez M. r. Raslan D. S. Akssira M. (2000). New sources and antifungal activity of sesquiterpene lactones. Fitoterapia 71 (1), 60–64. 10.1016/s0367-326x(99)00122-7 11449472

[B25] Beniddir M. A. Kang K. B. Genta-Jouve G. Huber F. Rogers S. Van Der Hooft J. J. J. N. (2021). Advances in decomposing complex metabolite mixtures using substructure-and network-based computational metabolomics approaches. Nat. Prod. Rep. 38 (11), 1967–1993. 10.1039/d1np00023c 34821250 PMC8597898

[B26] Boham B. Kocipai-Abyazan R. J. P. (1974). Flavonoids and condensed tannins from leaves of Hawaiian vaccinium vaticulatum and V. calycinium. Pac Sci. 48 (4), 458–463.

[B27] Boro H. Das S. Middha S. K. J. A. i. T. M. (2021). The therapeutic potential and the health benefits of Morus indica Linn.: a mini review. a mini Rev. 21, 241–252. 10.1007/s13596-020-00544-5

[B28] Bromke M. A. Hochmuth A. Tohge T. Fernie A. R. Giavalisco P. Burgos A. (2015). Liquid chromatography high-resolution mass spectrometry for fatty acid profiling. Plant J. 81 (3), 529–536. 10.1111/tpj.12739 25440443

[B29] Buchwalow I. B. Böcker W. (2010). Immunohistochemistry. Basics Methods 1, 1–149. 10.4016/16455.01

[B30] Chain E. P. Schrenk D. Bignami M. Bodin L. Chipman J. K. del Mazo J. (2023). Risk assessment of N-nitrosamines in food. EFSA J. 21 (3), e07884. 10.2903/j.efsa.2023.7884 36999063 PMC10043641

[B31] Chen H.-C. Chou C.-K. Lee S.-D. Wang J.-C. Yeh S.-F. (1995). Active compounds from Saussurea lappa Clarks that suppress hepatitis B virus surface antigen gene expression in human hepatoma cells. Antivir. Res. 27 (1), 99–109. 10.1016/0166-3542(94)00083-k 7486962

[B32] Chen P. Huo X. Liu W. Li K. Sun Z. Tian J. (2020). Apigenin exhibits anti-inflammatory effects in LPS-stimulated BV2 microglia through activating GSK3β/Nrf2 signaling pathway. Immunopharmacol. Immunotoxicol. 42 (1), 9–16. 10.1080/08923973.2019.1688345 31760890

[B33] Ciji A. Sahu N. P. Pal A. K. Akhtar M. S. (2013). Nitrite-induced alterations in sex steroids and thyroid hormones of Labeo rohita juveniles: effects of dietary vitamin E and l-tryptophan. Fish Physiology Biochem. 39 (5), 1297–1307. 10.1007/s10695-013-9784-8 23504103

[B34] Cvetković D. Živković V. Lukić V. Nikolić S. (2019). Sodium nitrite food poisoning in one family. Forensic Sci. Med. Pathology 15 (1), 102–105. 10.1007/s12024-018-0036-1 30293223

[B35] Deabes M. M. Fatah A.-E. Sally I. Salem S. H. E. Naguib K. M. J. E. J. (2021). Antimicrobial activity of bioactive compounds extract from Saussurea costus against food spoilage microorganisms. Egypt. J. Chem. 64 (6), 2833–2843. 10.21608/EJCHEM.202169572.3528

[B36] Dikshith T. S. S. Raizada R. B. Srivastava M. K. (1991). Long-term dietary study and development of no-observed-effect level (NOEL) of technical HCH to rats. J. Toxicol. Environ. Health 34 (4), 495–507. 10.1080/15287399109531585 1720467

[B37] DuBois M. Gilles K. A. Hamilton J. K. Rebers P. t. Smith F. (1956). Colorimetric method for determination of sugars and related substances. Anal. Chem. 28 (3), 350–356. 10.1021/ac60111a017

[B38] Edeoga H. O. Okwu D. Mbaebie B. (2005). Phytochemical constituents of some Nigerian medicinal plants. Afr. J. Biotechnol. 4 (7), 685–688. 10.5897/ajb2005.000-3127

[B39] Eissa M. A. Hashim Y. Z. H. El-Kersh D. M. Abd-Azziz S. S. Salleh H. M. Isa M. L. M. (2020). Metabolite Profiling of Aquilaria malaccensis leaf extract using Liquid Chromatography-Q-TOF-Mass spectrometry and investigation of its potential antilipoxygenase activity *in-vitro* . Processes 8 (2), 202. 10.3390/pr8020202

[B40] El-Demerdash F. M. Baghdadi H. H. Ghanem N. F. Mhanna A. B. A. (2020). Nephroprotective role of bromelain against oxidative injury induced by aluminium in rats. Environ. Toxicol. Pharmacol. 80, 103509. 10.1016/j.etap.2020.103509 33010469

[B41] El-Demerdash F. M. El-Sayed R. A. Abdel-Daim M. M. (2021a). Rosmarinus officinalis essential oil modulates renal toxicity and oxidative stress induced by potassium dichromate in rats. J. Trace Elem. Med. Biol. 67, 126791. 10.1016/j.jtemb.2021.126791 34022565

[B42] El-Demerdash F. M. El-Sayed R. A. Abdel-Daim M. M. (2021b). Hepatoprotective potential of Rosmarinus officinalis essential oil against hexavalent chromium-induced hematotoxicity, biochemical, histological, and immunohistochemical changes in male rats. Environ. Sci. Pollut. Res. 28 (14), 17445–17456. 10.1007/s11356-020-12126-8 33394444

[B43] El Gizawy H. A. El-Haddad A. E. Saadeldeen A. M. Boshra S. A. J. M. (2022). Tentatively identified (UPLC/T-TOF-MS/MS) compounds in the extract of <i>Saussurea costus</i> roots exhibit *in vivo* hepatoprotection via modulation of HNF-1α, sirtuin-1, C/ebpα, miRNA-34a and miRNA-223. Sirtuin-1, C/Ebpα, MiRNA-34a MiRNA-223 27 (9), 2802. 10.3390/molecules27092802 PMC9104236 35566153

[B44] El-Nabarawy N. A. Gouda A. S. Khattab M. A. Rashed L. A. (2020). Effects of nitrite graded doses on hepatotoxicity and nephrotoxicity, histopathological alterations, and activation of apoptosis in adult rats. Environ. Sci. Pollut. Res. 27 (12), 14019–14032. 10.1007/s11356-020-07901-6 32036525

[B45] Elsayed H. Mohamad M. Gabr O. Badria F. (2015). Saussurea lappa root extract accelerates the reversion of liver fibrosis induced by carbon tetrachloride in rats. Benha Med. J. 32, 116–125. 10.4103/1110-208x.180324

[B46] Elshaer S. E. Hamad G. M. Hafez E. E. Baghdadi H. H. El-Demerdash F. M. Simal-Gandara J. (2022). Root extracts of Saussurea costus as prospective detoxifying food additive against sodium nitrite toxicity in male rats. Food Chem. Toxicol. 166, 113225. 10.1016/j.fct.2022.113225 35691462

[B47] El-Sheikh N. M. Khalil F. A. (2011). l-Arginine and l-glutamine as immunonutrients and modulating agents for oxidative stress and toxicity induced by sodium nitrite in rats. Food Chem. Toxicol. 49 (4), 758–762. 10.1016/j.fct.2010.11.039 21130833

[B48] Elsherbiny N. M. Maysarah N. M. El-Sherbiny M. Al-Gayyar M. M. (2017). Renal protective effects of thymoquinone against sodium nitrite-induced chronic toxicity in rats: impact on inflammation and apoptosis. Life Sci. 180, 1–8. 10.1016/j.lfs.2017.05.005 28495515

[B49] Fadda L. M. Attia H. A. Al-Rasheed N. M. Ali H. M. Al-Rasheed N. M. (2018a). Roles of some antioxidants in modulation of cardiac myopathy induced by sodium nitrite via down-regulation of mRNA expression of NF-κB, Bax, and flt-1 and suppressing DNA damage. Saudi Pharm. J. 26 (2), 217–223. 10.1016/j.jsps.2017.12.008 30166919 PMC6111199

[B50] Fadda L. M. Attia H. A. Al-Rasheed N. M. Ali H. M. Al-Rasheed N. M. (2018b). Downregulation of flt-1 and HIF-1α gene expression by some antioxidants in rats under sodium nitrite-induced hypoxic stress. Dose-Response 16 (2), 1559325818776204. 10.1177/1559325818776204 29872369 PMC5974571

[B51] Fan H. Robetorye R. S. (2010). “Real-time quantitative reverse transcriptase polymerase chain reaction,” in RT-PCR protocols (Berlin, Germany: Springer), 199–213. 10.1007/978-1-60761-629-0_13 20300999

[B52] Fouad S. S. Mohi-Eldin M. M. Haridy M. A. Khalil A. M. (2017). Ameliorative effects of ascorbic acid (vit. C) against sodium nitrite toxicity in albino rats: hematological, biochemical and histopathological studies. American-Eurasian J. Toxicol. Sci. 9 (1), 01–06.

[B53] Fromm O. DeGolier T. (2021). The contractile capabilities of various herbal constituents on uterine smooth muscle and their shared constituent presence involved with anti-inflammatory/antioxidant mechanisms. J. Pharmacogn. Phytochemistry 10 (4), 28–37.

[B54] Fukuda K. Akao S. Ohno Y. Yamashita K. Fujiwara H. (2001). Inhibition by costunolide of phorbol ester-induced transcriptional activation of inducible nitric oxide synthase gene in a human monocyte cell line THP-1. Cancer Lett. 164 (1), 7–13. 10.1016/s0304-3835(00)00704-7 11166910

[B55] Gallo M. Gгўmiz F. (2023). Choline: an essential nutrient for human health. Nutrients 15, 2900. 10.3390/nu15132900 37447226 PMC10343572

[B56] Gilani A. H. Shah A. J. Yaeesh S. (2007). Presence of cholinergic and calcium antagonist constituents in Saussurea lappa explains its use in constipation and spasm. Phytotherapy Res. 21 (6), 541–544. 10.1002/ptr.2098 17295386

[B57] Ginwala R. Bhavsar R. Chigbu D. G. I. Jain P. Khan Z. K. (2019). Potential role of flavonoids in treating chronic inflammatory diseases with a special focus on the anti-inflammatory activity of apigenin. Antioxidants 8, 35. 10.3390/antiox8020035 30764536 PMC6407021

[B58] Gluhcheva Y. Ivanov I. Petrova E. Pavlova E. Vladov I. (2012). Sodium nitrite-induced hematological and hemorheological changes in rats. Ser. Biomechanics 27, 4–53.

[B59] Grant D. Butler W. H. (1989). Chronic toxicity of sodium nitrite in the male F344 rat. Food Chem. Toxicol. 27 (9), 565–571. 10.1016/0278-6915(89)90015-x 2807101

[B60] Hafez E. Hashem M. Balbaa M. El-Saadani M. Ahmed S. (2013). Induction of new defensin genes in tomato plants via pathogens-biocontrol agent interaction. J. Plant Pathol. Microb. 4 (167), 2. 10.4172/2157-7471.1000167

[B61] Hamad G. Hafez E. Khaled A. A. Amara A. (2018). Amino acids diets as model for investigating cancer induced by acrylamide produced during wrong food cooking. SOJ Biochem. 4 (1), 1–14. 10.15226/2376-4589/4/1/00126

[B62] Hammoud G. (2014). Protective effect of grape seeds extract against sodium nitrite-induced toxicity and oxidative stress in albino rats. Al-Azhar J. Pharm. Sci. 49, 1–34. 10.21608/ajps.2014.6956

[B63] Hanh T. T. H. Cham P. T. My N. T. T. Cuong N. T. Dang N. H. Quang T. H. (2021). Sesquiterpenoids from Saussurea costus. Nat. Prod. Res. 35 (9), 1399–1405. 10.1080/14786419.2019.1650357 31402701

[B64] Hassan H. A. El-Agmy S. M. Gaur R. L. Fernando A. Raj M. H. Ouhtit A. (2009). *In vivo* evidence of hepato-and reno-protective effect of garlic oil against sodium nitrite-induced oxidative stress. Int. J. Biol. Sci. 5 (3), 249–255. 10.7150/ijbs.5.249 19305642 PMC2659008

[B65] Hassan H. A. Hafez H. S. Zeghebar F. E. (2010). Garlic oil as a modulating agent for oxidative stress and neurotoxicity induced by sodium nitrite in male albino rats. Food Chem. Toxicol. 48 (7), 1980–1985. 10.1016/j.fct.2010.05.001 20457208

[B66] Hassan H. A. Yousef M. I. (2010). Ameliorating effect of chicory (Cichorium intybus L.)-supplemented diet against nitrosamine precursors-induced liver injury and oxidative stress in male rats. Food Chem. Toxicol. 48 (8-9), 2163–2169. 10.1016/j.fct.2010.05.023 20478349

[B67] Hassan R. O. Ali D. S. (2010). Determination of content levels of nitrogen species (Nitrite, Nitrate, and N-Nitrosamines) in processed meat consumed in Erbil City. Der Pharma Chem. 2 (6), 31–37.

[B68] Heinrich M. Mah J. Amirkia V. (2021). Alkaloids used as medicines: structural phytochemistry meets biodiversity-an update and forward look. Molecules 26 (7), 1836. 10.3390/molecules26071836 33805869 PMC8036335

[B69] Helal E. Zaahkouk S. A. Mekkawy H. A. (2000). Effect of some food colorants (synthetic andNatural products) of young albino rats. Egypt. J. Hosp. Med. 1, 103–113. 10.21608/ejhm.2000.11021

[B70] Helal E. G. E. Almutairi A. Abdelaziz M. A. Mohamed A. A. H. (2020). Adverse effects of fast green, sodium nitrate and Glycine on some physiological parameters. Egypt. J. Hosp. Med. 80 (3), 964–970. 10.21608/ejhm.2020.104304

[B71] Helal E. G. E. El-Sayed R. A. A. Hedeab G. El-Gamal M. S. (2017). Effects of some food additives on some biochemical parameters in young male albino rats and the ameliorative role of royal jelly. Egypt. J. Hosp. Med. 67 (2), 605–613. 10.12816/0037812

[B72] Helal E. M A.-K. H A. W. S Z. Soliman G. Z. A. (2008). Biochemical studies on the effect of sodium nitrite and/or glutathione treatment on male rats. Egypt. J. Hosp. Med. 30 (1), 25–38. 10.21608/ejhm.2008.17650

[B73] Huestis M. A. (2007). Human cannabinoid pharmacokinetics. Chem. Biodivers. 4 (8), 1770–1804. 10.1002/cbdv.200790152 17712819 PMC2689518

[B74] Imam O. S. Mohammed M. H. A.-G. (2013). Antioxidant, anti-inflammatory and hepatoprotective effects of silymarin on hepatic dysfunction induced by sodium nitrite. Eur. Cytokine Netw. 24 (3), 114–121. 10.1684/ecn.2013.0341 24225033

[B75] Jensen F. B. (2007). Nitric oxide formation from nitrite in zebrafish. J. Exp. Biol. 210 (19), 3387–3394. 10.1242/jeb.008748 17872992

[B76] Jia Q.-Q. Wang J.-C. Long J. Zhao Y. Chen S.-J. Zhai J.-D. (2013). Sesquiterpene lactones and their derivatives inhibit high glucose-induced NF-κB activation and MCP-1 and TGF-β1 expression in rat mesangial cells. Molecules 18, 13061–13077. 10.3390/molecules181013061 24152676 PMC6269856

[B77] Johnson R. J. Nakagawa T. Jalal D. Sгўnchez-Lozada L. G. Kang D.-H. Ritz E. (2013). Uric acid and chronic kidney disease: which is chasing which? Nephrol. Dial. Transplant. 28 (9), 2221–2228. 10.1093/ndt/gft029 23543594 PMC4318947

[B78] Julianti T. (2014) Discovery of natural antiprotozoals from medicinal plants Saussurea costus and Carica papaya. Basel, Switzerland: University_of_Basel.

[B79] Kadam P. Bodhankar S. (2013). Analgesic and anti-inflammatory activity of seed extracts of diplocyclos palmatus (L) C. Jeffrey. Int. J. Pharma Bio Sci. 4, P970–P978.

[B80] Kang J. S. Yoon Y. D. Lee K. H. Park S.-K. Kim H. M. (2004). Costunolide inhibits interleukin-1beta expression by down-regulation of AP-1 and MAPK activity in LPS-stimulated RAW 264.7 cells. Biochem. Biophysical Res. Commun. 313 (1), 171–177. 10.1016/j.bbrc.2003.11.109 14672714

[B81] Kapoor R. Huang Y.-S. (2006). Gamma linolenic acid: an antiinflammatory omega-6 fatty acid. Curr. Pharm. Biotechnol. 7 (6), 531–534. 10.2174/138920106779116874 17168669

[B82] Kashyap P. Shikha D. Thakur M. Aneja A. (2022). Functionality of apigenin as a potent antioxidant with emphasis on bioavailability, metabolism, action mechanism and *in vitro* and *in vivo* studies: a review. J. Food Biochem. 46 (4), e13950. 10.1111/jfbc.13950 34569073

[B83] Khalid A. Rehman U. Sethi A. Khilji S. Fatima U. Khan M. I. (2011). Antimicrobial activity analysis of extracts of Acacia modesta, Artimisia absinthium, Nigella sativa and Saussurea lappa against Gram positive and Gram negative microorganisms. Afr. J. Biotechnol. 10 (22), 4574–4580. 10.5897/AJB11.109

[B84] Khalil A. (2016). Ameliorative effects of ascorbic acid (vit. C) against sodium nitrite toxicity in albino rats: hematological. Biochem. Histopathol. Stud.

[bib159] Khirallah S. M. Ramadan H. M. M. Shawky A. Qahl S. H. Baty R. S. Alqadri N. (2022). Development of novel 1,3-disubstituted-2-thiohydantoin analogues with potent anti-inflammatory activity; *in vitro* and *in silico* assessments. Molecules 27. 10.3390/molecules27196271 PMC9573447 36234810

[B85] Ko S. G. Kim H.-P. Jin D.-H. Bae H.-S. Kim S. H. Park C.-H. (2005). Saussurea lappa induces G2-growth arrest and apoptosis in AGS gastric cancer cells. Cancer Lett. 220 (1), 11–19. 10.1016/j.canlet.2004.06.026 15737683

[B86] Kolaylı S. Sahin H. Ulusoy E. Tarhan Ö. (2010). Phenolic composition and antioxidant capacities of Helichrysum plicatum. Hacet. J. Biol. Chem. 38, 269–276.

[B87] Koriem K. M. M. Tharwat H. A. K. (2023). Malic acid improves behavioral, biochemical, and molecular disturbances in the hypothalamus of stressed rats. JIN 22 (4), 98. 10.31083/j.jin2204098 37519180

[B88] Kulkarni S. (2001). Immunostimulant activity of inulin isolated from Saussurea lappa roots. Indian J. Pharm. Sci. 63 (4), 292.

[B89] Kumar J. Pundir M. (2022). Phytochemistry and pharmacology of Saussurea genus (Saussurea lappa, Saussurea costus, Saussurea obvallata, Saussurea involucrata). Mater. Proc. 56, 1173–1181. 10.1016/j.matpr.2021.11.145

[B90] Le-Vinh B. AkkuЕџ-DaДџdeviren Z. B. Le N. M. N. Nazir I. Bernkop-SchnГјrch A. (2022). Alkaline phosphatase: a reliable endogenous partner for drug delivery and Diagnostics. Adv. Ther. 5 (2), 2100219. 10.1002/adtp.202100219

[B91] Li H. Li S. Yang H. Wang Y. Wang J. Zheng N. J. T. (2019). l-Proline alleviates kidney injury caused by AFB1 and AFM1 through regulating excessive apoptosis of kidney cells. Toxins (Basel) 11 (4), 226. 10.3390/toxins11040226 30995739 PMC6521284

[B92] Liang P. Pardee A. B. (1992). Differential display of eukaryotic messenger RNA by means of the polymerase chain reaction. Science 257 (5072), 967–971. 10.1126/science.1354393 1354393

[B93] Lin Y. Shi R. Wang X. Shen H.-M. (2008). Luteolin, a flavonoid with potential for cancer prevention and therapy. Curr. Cancer Drug Targets 8 (7), 634–646. 10.2174/156800908786241050 18991571 PMC2615542

[B94] Livak K. J. Schmittgen T. D. (2001). Analysis of relative gene expression data using real-time quantitative PCR and the 2(-Delta Delta C(T)) Method. methods 25 (4), 402–408. 10.1006/meth.2001.1262 11846609

[B95] Lotfi M.-S. Rassouli F. B. (2024). Natural flavonoid apigenin, an effective agent against nervous system cancers. Mol. Neurobiol. 10.1007/s12035-024-03917-y 38206472

[B96] Lu X. Zhao X. Bai C. Zhao C. Lu G. Xu G. J. J. C. B. (2008). LC–MS-based metabonomics analysis. J. Chromatogr. B Anal. Technol. Biomed. Life Sci. 866 (1-2), 64–76. 10.1016/j.jchromb.2007.10.022 17983864

[B97] Madrigal-Santillán E. Madrigal-Bujaidar E. Álvarez-González I. Sumaya-Martínez M. T. Gutiérrez-Salinas J. Bautista M. (2014). Review of natural products with hepatoprotective effects. World J. gastroenterology 20 (40), 14787–14804. 10.3748/wjg.v20.i40.14787 25356040 PMC4209543

[B98] Mehedint M. G. Zeisel S. H. (2013). Choline's role in maintaining liver function: new evidence for epigenetic mechanisms. Curr. Opin. Clin. Nutr. metabolic care 16 (3), 339–345. 10.1097/MCO.0b013e3283600d46 23493015 PMC3729018

[B99] Milkowski A. Garg H. K. Coughlin J. R. Bryan N. S. (2010). Nutritional epidemiology in the context of nitric oxide biology: a risk–benefit evaluation for dietary nitrite and nitrate. Nitric oxide 22 (2), 110–119. 10.1016/j.niox.2009.08.004 19748594

[B100] Mir M. A. Parihar K. Tabasum U. Kumari E. J. J. M. P. S. (2016). Estimation of alkaloid, saponin and flavonoid, content in various extracts of Crocus sativa. J. Med. Plants Stud. 4 (5), 171–174.

[bib160] Mohamed D. I. El-Waseef D. A. E.-D. A. Nabih E. S. El-Kharashi O. A. El-Kareem H. F. A. Nahas H. H. A. (2022a). Acetylsalicylic acid suppresses alcoholism-induced cognitive impairment associated with atorvastatin intake by targeting cerebral mirna155 and nlrp3: *in vivo*, and *in silico* study. Pharmaceutics 14. 10.3390/pharmaceutics14030529 PMC8948796 35335908

[bib161] Mohamed D. I. Ezzat S. F. Elayat W. M. El-Kharashi O. A. El-Kareem H. F. A. Nahas H. H. A. (2022b). Hepatoprotective role of carvedilol against ischemic hepatitis associated with acute heart failure via targeting mirna-17 and mitochondrial dynamics-related proteins: an *in vivo* and *in silico* study. Pharmaceuticals 15, 832. 10.3390/ph15070832 35890131 PMC9319470

[B101] Moujir L. Callies O. Sousa P. M. Sharopov F. Seca A. M. J. A. S. (2020). Applications of sesquiterpene lactones: a review of some potential success cases. Appl. Sci. 10 (9), 3001. 10.3390/app10093001

[B102] Murai T. Matsuda S. (2023). The chemopreventive effects of chlorogenic acids, phenolic compounds in coffee, against inflammation, cancer, and neurological diseases. Molecules 28 (5), 2381. 10.3390/molecules28052381 36903626 PMC10005755

[B103] Muruganathan N. Dhanapal A. R. Baskar V. Muthuramalingam P. Selvaraj D. Aara H. (2022). Recent updates on source, biosynthesis, and therapeutic potential of natural flavonoid luteolin: a review. Metabolites 12 (11), 1145. 10.3390/metabo12111145 36422285 PMC9696498

[B104] Nii T. Yumoto H. Hirota K. Miyake Y. J. B. O. H. (2019). Anti-inflammatory effects of olanexidine gluconate on oral epithelial cells. BMC Oral Health. 19 (1), 239–247. 10.1186/s12903-019-0932-0 31703580 PMC6839112

[B105] Obadoni B. Ochuko P. J. G. J. (2002). Phytochemical studies and comparative efficacy of the crude extracts of some haemostatic plants in Edo and Delta States of Nigeria. Glob. J. Pure Appl. Sci. 8 (2), 203–208. 10.4314/gjpas.v8i2.16033

[B106] Ohtani M. Maruyama K. Sugita M. Kobayashi K. (2001). Amino acid supplementation affects hematological and biochemical parameters in elite rugby players. Biosci. Biotechnol. Biochem. 65 (9), 1970–1976. 10.1271/bbb.65.1970 11676007

[B107] Okazaki Y. Otsuki H. Narisawa T. Kobayashi M. Sawai S. Kamide Y. (2013). A new class of plant lipid is essential for protection against phosphorus depletion. Nat. Commun. 4 (1), 1510. 10.1038/ncomms2512 23443538 PMC3586718

[B108] Osman A. Imbabi T. A. El-Hadary A. Sabeq I. I. Edris S. N. Merwad A.-R. (2021). Health aspects, growth performance, and meat quality of rabbits receiving diets supplemented with lettuce fertilized with whey protein hydrolysate substituting nitrate. Biomolecules 11 (6), 835. 10.3390/biom11060835 34205142 PMC8227087

[B109] Ouyang Y. Rong Y. Wang Y. Guo Y. Shan L. Yu X. (2021). A systematic study of the mechanism of acacetin against sepsis based on network pharmacology and experimental validation. Front. Pharmacol. 12, 683645. 10.3389/fphar.2021.683645 34483900 PMC8415621

[B110] Özen H. Kamber U. Karaman M. Gül S. Atakişi E. Özcan K. (2014). Histopathologic, biochemical and genotoxic investigations on chronic sodium nitrite toxicity in mice. Exp. Toxicol. pathology 66 (8), 367–375. 10.1016/j.etp.2014.05.003 24947405

[B111] Parama D. Boruah M. Yachna K. Rana V. Banik K. Harsha C. (2020). Diosgenin, a steroidal saponin, and its analogs: effective therapies against different chronic diseases. diseases 260, 118182. 10.1016/j.lfs.2020.118182 32781063

[B112] Peter M. C. S. (2011). The role of thyroid hormones in stress response of fish. General Comp. Endocrinol. 172 (2), 198–210. 10.1016/j.ygcen.2011.02.023 21362420

[B113] Porter W. P. Green S. M. Debbink N. L. Carlson I. (1993). Groundwater pesticides: interactive effects of low concentrations of carbamates aldicarb and methomyl and the triazine metribuzin on thyroxine and somatotropin levels in white rats. J. Toxicol. Environ. Health 40 (1), 15–34. 10.1080/15287399309531773 8360940

[B114] Poschner S. Maier-Salamon A. Zehl M. Wackerlig J. Dobusch D. Pachmann B. (2017). The impacts of genistein and daidzein on estrogen conjugations in human breast cancer cells: a targeted metabolomics approach. Front. Pharmacol. 8, 699. 10.3389/fphar.2017.00699 29051735 PMC5633874

[B115] Radwan E. Hussein H. Kk A. Barakat I. (2020). The possible effects of sodium nitrite and sodium benzoate as food additives on the liver in male rats. J. Adv. Biol. 13, 2347–6893. 10.24297/jab.v13i.8717

[B116] Rahman M. M. Rahaman M. S. Islam M. R. Rahman F. Mithi F. M. Alqahtani T. (2022). Role of phenolic compounds in human disease: current knowledge and future prospects. Molecules 27 (1), 233. 10.3390/molecules27010233 35011465 PMC8746501

[B117] Roager H. M. Licht T. R. (2018). Microbial tryptophan catabolites in health and disease. Nat. Commun. 9 (1), 3294. 10.1038/s41467-018-05470-4 30120222 PMC6098093

[B118] Robinson T. Van Burden W. J. J. A. F. C. (1981). Formation of complexes between protein and tannins acid. J. Agric. Food Chem. 1, 77. 10.1021/jf60164a003

[B119] Romanova D. Vachalkova A. Cipak L. Ovesna Z. Rauko P. J. N. (2001). Study of antioxidant effect of apigenin, luteolin and quercetin by DNA protective method. Neoplasma 48 (2), 104–107. 11478688

[B121] Ruan Z. Yang Y. Wen Y. Zhou Y. Fu X. Ding S. (2014). Metabolomic analysis of amino acid and fat metabolism in rats with l-tryptophan supplementation. Amino Acids 46 (12), 2681–2691. 10.1007/s00726-014-1823-y 25139634

[B122] Saif-Al-Islam M. J. S. M. J. (2020). Saussurea costus may help Treat. COVID-19 24 (3), 6–17. 10.21608/SMJ.2020.31144.1163

[B123] Salama M. F. Abbas A. Darweish M. M. El-Hawwary A. A. Al-Gayyar M. M. (2013). Hepatoprotective effects of cod liver oil against sodium nitrite toxicity in rats. Pharm. Biol. 51 (11), 1435–1443. 10.3109/13880209.2013.796564 23862714

[B124] Saleh-e-In M. M. Sultana N. Hossain M. N. Hasan S. Islam M. (2016). Pharmacological effects of the phytochemicals of Anethum sowa L. root extracts. BMC Complementary Altern. Med. 16 (1), 464–514. 10.1186/s12906-016-1438-9 27842527 PMC5396551

[bib162] Salem M. G. El-Maaty D. M. A. El-Deen Y. I. M. Elesawy B. H. Askary A. E. Saleh A. (2022). Novel 1,3-thiazole analogues with potent activity against breast cancer: a design, synthesis, *in vitro*, and *in silico* stu. Molecules 27. 10.3390/molecules27154898 PMC9370021 35956848

[bib163] Samaha D. Hamdo H. H. Cong X. Schumacher F. Banhart S. Aglar Ö. (2020). Liposomal fret assay identifies potent drug-like inhibitors of the ceramide transport protein (CERT). Chem.–Eur. J. 26, 16616–16621. 10.1002/chem.202003283 33047409 PMC7756341

[B125] Sasaki S. Futagi Y. Kobayashi M. Ogura J. Iseki K. (2015). Functional characterization of 5-oxoproline transport via SLC16A1/MCT1*. J. Biol. Chem. 290 (4), 2303–2311. 10.1074/jbc.M114.581892 25371203 PMC4303682

[B126] Sawada Y. Kuwahara A. Nagano M. Narisawa T. Sakata A. Saito K. (2009). Omics-based approaches to methionine side chain elongation in Arabidopsis: characterization of the genes encoding methylthioalkylmalate isomerase and methylthioalkylmalate dehydrogenase. Plant Cell Physiol. 50 (7), 1181–1190. 10.1093/pcp/pcp079 19493961 PMC2709551

[B127] Selvi E. K. Turumtay H. Demir A. Turumtay E. A. (2018). Phytochemical profiling and evaluation of the hepatoprotective effect of cuscuta campestris by high-performance liquid chromatography with diode array detection. Anal. Lett. 51 (10), 1464–1478. 10.1080/00032719.2017.1382502

[B128] Seo M. Jikumaru Y. Kamiya Y. (2011). Profiling of hormones and related metabolites in seed dormancy and germination studies. Methods Mol. Biol. 773, 99–111. 10.1007/978-1-61779-231-1_7 21898252

[B129] Shahaf N. Rogachev I. Heinig U. Meir S. Malitsky S. Battat M. (2016). The WEIZMASS spectral library for high-confidence metabolite identification. Nat. Commun. 7 (1), 12423. 10.1038/ncomms12423 27571918 PMC5013563

[B130] Sindelar J. J. Milkowski A. L. (2012). Human safety controversies surrounding nitrate and nitrite in the diet. Nitric oxide 26 (4), 259–266. 10.1016/j.niox.2012.03.011 22487433

[B131] Singh R. Chahal K. Singla N. J. J. o. P. (2017). Chemical composition and pharmacological activities of Saussurea lappa. A Rev. 6 (4), 1298–1308.

[B132] Singh S. Grewal S. Sharma N. Behl T. Gupta S. Anwer M. K. (2023). Unveiling the pharmacological and nanotechnological facets of daidzein: present state-of-the-art and future perspectives. Molecules 28 (4), 1765. 10.3390/molecules28041765 36838751 PMC9958968

[B133] Singh S. Gupta P. Meena A. Luqman S. (2020). Acacetin, a flavone with diverse therapeutic potential in cancer, inflammation, infections and other metabolic disorders. Food Chem. Toxicol. 145, 111708. 10.1016/j.fct.2020.111708 32866514

[B134] Soliman M. M. Aldhahrani A. Elshazly S. A. Shukry M. Abouzed T. K. (2022). Borate ameliorates sodium nitrite-induced oxidative stress through regulation of oxidant/antioxidant status: involvement of the Nrf2/HO-1 and NF-κB pathways. Biol. Trace Elem. Res. 200 (1), 197–205. 10.1007/s12011-021-02613-5 33559025

[B135] Srinivasan K. Radhakrishnamurty R. (1988). Biochemical changes produced by beta- and gamma-hexachlorocyclohexane isomers in albino rats. J. Environ. Sci. Health, Part B 23 (4), 367–386. 10.1080/03601238809372612 2461408

[B136] Srivastava S. Singh P. Jha K. K. Mishra G. Srivastava S. Khosa R. L. (2012). Evaluation of anti-arthritic potential of the methanolic extract of the aerial parts of Costus speciosus. J. Ayurveda Integr. Med. 3 (4), 204–208. 10.4103/0975-9476.104443 23326092 PMC3545241

[B137] Sullivan G. A. Jackson-Davis A. L. Niebuhr S. E. Xi Y. Schrader K. D. Sebranek J. G. (2012). Inhibition of Listeria monocytogenes using natural antimicrobials in no-nitrate-or-nitrite-added ham. J. food Prot. 75 (6), 1071–1076. 10.4315/0362-028X.JFP-11-511 22691474

[B138] Sunkara Y. Robinson A. Babu K. Naidu V. Vishnuvardhan M. Ramakrishna S. (2010). Anti-inflammatory and cytotoxic activity of chloroform extract of roots of Saussurea lappa Clarke. J. Pharm. Res. 3 (8), 1775–1778.

[B139] Sutar N. Garai R. Sharma U. S. Singh N. Roy S. D. (2011). Antiulcerogenic activity of Saussurea lappa root. Int. J. Pharm. Life Sci. 2 (1), 516–520.

[B140] Tag H. M. Khaled H. E. Ismail H. A. El-Shenawy N. S. (2016a). Evaluation of anti-inflammatory potential of the ethanolic extract of the Saussurea lappa root (costus) on adjuvant-induced monoarthritis in rats. J. basic Clin. physiology Pharmacol. 27 (1), 71–78. 10.1515/jbcpp-2015-0044 26479340

[B141] Tag H. M. Khaled H. E. Ismail H. A. A. El-Shenawy N. S. (2016b). Evaluation of anti-inflammatory potential of the ethanolic extract of the Saussurea lappa root (costus) on adjuvant-induced monoarthritis in rats. J. Basic Clin. Physiol. Pharmacol. 27 (1), 71–78. 10.1515/jbcpp-2015-0044 26479340

[B142] Tawfek N. A. (2015). H; abdalla, AA; fargali, S adverse effects of some food additives in adult male albino rats. Curr. Sci. Int. 4 (4), 525–537.

[B143] Teng H. Lin Q. Li K. Yuan B. Song H. Peng H. (2017). Hepatoprotective effects of raspberry (Rubus coreanus Miq.) seed oil and its major constituents. Food Chem. Toxicol. 110, 418–424. 10.1016/j.fct.2017.09.010 28899773

[B144] Tohge T. Fernie A. R. J. N. (2010). Combining genetic diversity, informatics and metabolomics to facilitate annotation of plant gene function. Nat. Protoc. 5 (6), 1210–1227. 10.1038/nprot.2010.82 20539294

[B145] Tsugawa H. Cajka T. Kind T. Ma Y. Higgins B. Ikeda K. (2015). MS-DIAL: data-independent MS/MS deconvolution for comprehensive metabolome analysis. Nat. Methods. 12 (6), 523–526. 10.1038/nmeth.3393 25938372 PMC4449330

[B146] Tuncel A. T. Ruppert T. Wang B.-T. Okun J. G. Kг¶lker S. Morath M. A. (2015). Maleic acid--but not structurally related methylmalonic acid--interrupts energy metabolism by impaired calcium homeostasis. PLOS ONE 10 (6), e0128770. 10.1371/journal.pone.0128770 26086473 PMC4473014

[B147] Tungmunnithum D. Thongboonyou A. Pholboon A. Yangsabai A. (2018). Flavonoids and other phenolic compounds from medicinal plants for pharmaceutical and medical aspects: an overview. Med. (Basel, Switz.) 5 (3), 93. 10.3390/medicines5030093 30149600 PMC6165118

[B148] Uchida K. Nomura Y. Takase H. Tasaki T. Seo S. Hayashi Y. (1990). Effect of vitamin C depletion on serum cholesterol and lipoprotein levels in ODS (od/od) rats unable to synthesize ascorbic acid. J. Nutr. 120 (10), 1140–1147. 10.1093/jn/120.10.1140 2213244

[B149] Vignesh A. Pradeepa Veerakumari K. Selvakumar S. Rakkiyappan R. Vasanth K. J. T. (2021). Nutritional assessment, antioxidant, anti-inflammatory and antidiabetic potential of traditionally used wild plant. Berberis tinctoria Lesch 5 (2), 71–92. 10.30495/tpr.2021.1914719.1186

[B150] Wang J. Liu Y.-T. Xiao L. Zhu L. Wang Q. Yan T. (2014a). Anti-inflammatory effects of apigenin in lipopolysaccharide-induced inflammatory in acute lung injury by suppressing COX-2 and NF-kB pathway. Inflammation 37 (6), 2085–2090. 10.1007/s10753-014-9942-x 24958013

[B151] Wang J. Liu Y.-T. Xiao L. Zhu L. Wang Q. Yan T. J. I. (2014b). Anti-inflammatory effects of apigenin in lipopolysaccharide-induced inflammatory in acute lung injury by suppressing COX-2 and NF-kB pathway. Inflammation. 37, 2085–2090. 10.1007/s10753-014-9942-x 24958013

[B152] Wang Y. Hayatsu M. Fujii T. (2009). Extraction of bacterial RNA from soil: challenges and solutions. Microbes Environ. 1202170350. 10.1264/jsme2.me11304 22791042 PMC4036013

[B153] Wei J. Bhatt S. Chang L. M. Sampson H. A. Masilamani M. (2012). Isoflavones, genistein and daidzein, regulate mucosal immune response by suppressing dendritic cell function. PLOS ONE 7 (10), e47979. 10.1371/journal.pone.0047979 23110148 PMC3478285

[B154] White K. Someya S. (2022). The roles of NADPH and isocitrate dehydrogenase in cochlear mitochondrial antioxidant defense and aging. Hear. Res. 427, 108659. 10.1016/j.heares.2022.108659 36493529 PMC11446251

[B155] Wu L. Zhang C. Long Y. Chen Q. Zhang W. Liu G. (2022). Food additives: from functions to analytical methods. Crit. Rev. Food Sci. Nutr. 62 (30), 8497–8517. 10.1080/10408398.2021.1929823 34058921

[B156] Yaeesh S. Jamal Q. Shah A. J. Gilani A. H. (2010). Antihepatotoxic activity of Saussurea lappa extract on D-galactosamine and lipopolysaccharide-induced hepatitis in mice. Phytotherapy Res. 24 (S2), S229–S232. 10.1002/ptr.3089 20041433

[B157] Yılmaz S. Ünal F. Yüzbaşıoğlu D. (2009). The *in vitro* genotoxicity of benzoic acid in human peripheral blood lymphocytes. Cytotechnology 60 (1-3), 55. 10.1007/s10616-009-9214-z 19642007 PMC2780543

[B158] Zhao F. Xu H. He E.-Q. Jiang Y.-T. Liu K. (2008). Inhibitory effects of sesquiterpenes from Saussurea lappa on the overproduction of nitric oxide and TNF-alpha release in LPS-activated macrophages. J. Asian Nat. Prod. Res. 10 (11), 1045–1053. 10.1080/10286020802274037 19031245

