# Correction: Supplementation of *Saussurea costus* root alleviates sodium nitrite-induced hepatorenal toxicity by modulating metabolic profile, inflammation, and apoptosis

**DOI:** 10.3389/fphar.2026.1892976

**Published:** 2026-08-03

**Authors:** Samy E. Elshaer, Gamal M. Hamad, Sherien E. Sobhy, Amira M. Galal Darwish, Hoda H. Baghdadi, Hebatallah H. Abo Nahas, Fatma M. El-Demerdash, Sanaa S. A. Kabeil, Abdulmalik S. Altamimi, Ebtesam Al-Olayan, Maha Alsunbul, Omaima Kamel Docmac, Mariusz Jaremko, Elsayed E. Hafez, Essa M. Saied

**Affiliations:** 1 Department of Environmental Studies, Institute of Graduate Studies and Research, Alexandria University, Alexandria, Egypt; 2 Department of Food Technology, Arid Lands Cultivation Research Institute (ALCRI), City of Scientific Research and Technological Applications (SRTA-City), Alexandria, Egypt; 3 Department of Plant Protection and Biomolecular Diagnosis, Arid Lands Cultivation Research Institute (ALCRI), City of Scientific Research and Technological Applications (SRTA-City), Alexandria, Egypt; 4 Food Industry Technology Program, Faculty of Industrial and Energy Technology, Borg Al Arab Technological University (BATU), Alexandria, Egypt; 5 Zoology Department, Faculty of Science, Port Said University, Port Said, Egypt; 6 Department of Protein Research, Genetic Engineering and Biotechnology Research Institute (GEBRI), City of Scientific Research and Technological Applications (SRTA-City), Alexandria, Egypt; 7 Department of Pharmaceutical Chemistry, College of Pharmacy, Prince Sattam Bin Abdulaziz University, Alkharj, Saudi Arabia; 8 Department of Zoology, College of Science, King Saud University, Riyadh, Saudi Arabia; 9 Department of Pharmaceutical Sciences., College of Pharmacy, Princess Nourah bint Abdulrahman University, Riyadh, Saudi Arabia; 10 Anatomy and Embryology Department, Faculty of Medicine, Tanta University, Tanta, Egypt; 11 Smart-Health Initiative and Red Sea Research Center, Division of Biological and Environmental Sciences and Engineering, King Abdullah University of Science and Technology, Thuwal, Saudi Arabia; 12 Chemistry Department (Biochemistry Division), Faculty of Science, Suez Canal University, Ismailia, Egypt; 13 Institute for Chemistry, Humboldt Universität zu Berlin, Berlin, Germany

**Keywords:** food additives, sodium nitrite, Saussurea costus, phytochemical profile, hepatorenal protective, metabolic analysis, histopathology analysis, immunohistochemistry analysis

In the published article, there was an error in Figure 11 as published. During retrospective review of the original source files, the authors identified an inadvertent misplacement in the representative immunohistochemical panels corresponding to G1 and G3 during preparation of the final composite figure, resulting in overlapping regions between these two representative panels.

**FIGURE 11:**
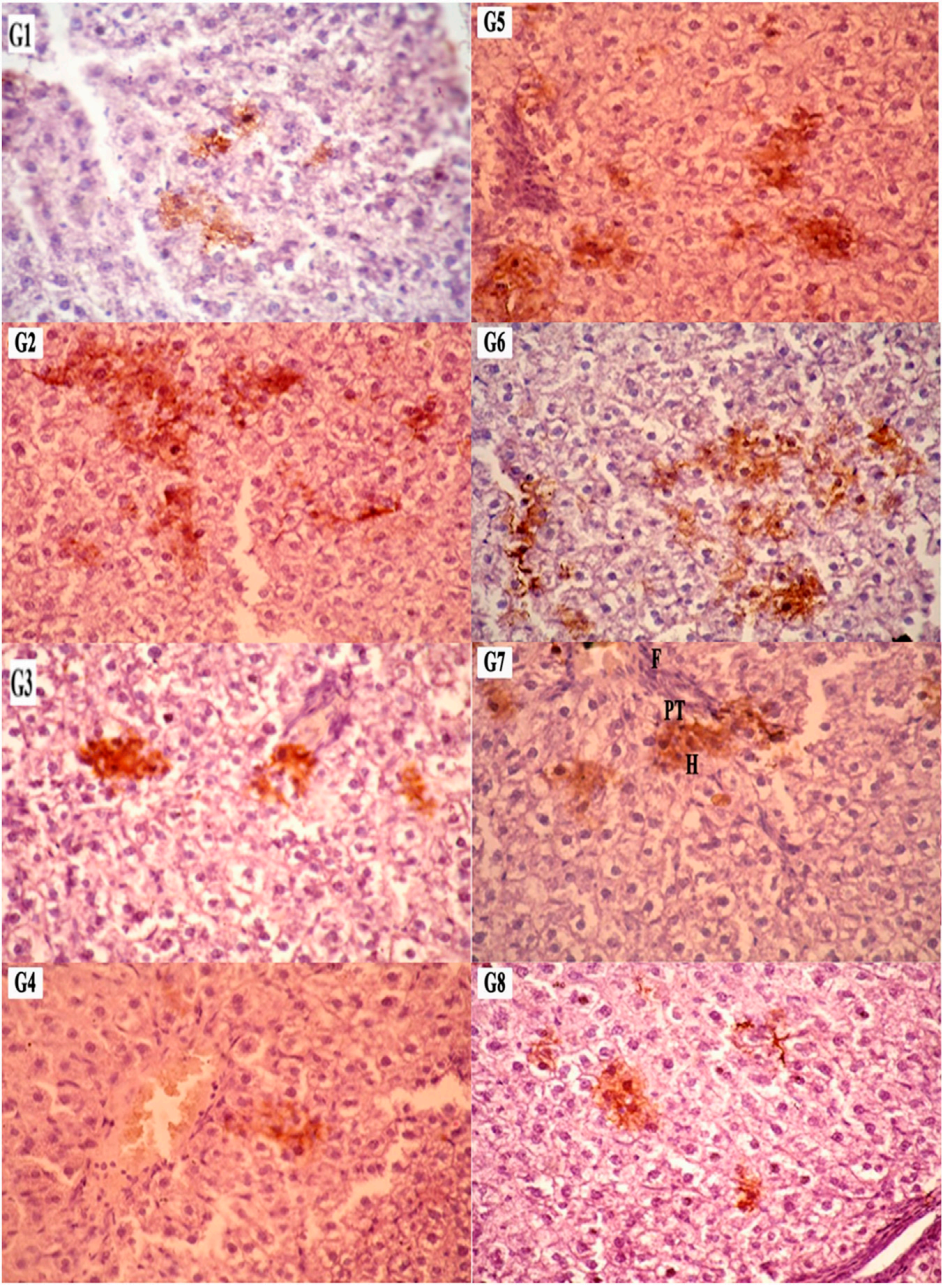
Photomicrographs of TGF-β immunoreactivity expression as brownish color in rat liver sections in different groups. G1: Control G2: SCREE (200 mg/kg BW), G3: SCREE (400 mg/kg BW), G4: SCREE (high dose), G5: NaNO_2_, G6: SCREE (200 mg/kg BW) + NaNO_2_, G7: SCREE (400 mg/kg BW) + NaNO_2_, and G8: SCREE (600 mg/kg BW) + NaNO_2_, PT: portal tract, H: hepatocyte, F: fibrotic cells (DAB 400x stain).

The corrected Figure 11 and its caption appear below.

The original version of this article has been updated.

