# Supplementary material for: Supplementation of Saussurea costus root alleviates sodium nitrite-induced hepatorenal toxicity by modulating metabolic profile, inflammation, and apoptosis
Source: Front Pharmacol. 2024 May 30;15:1378249. doi: 10.3389/fphar.2024.1378249 (PMC11177093; doi:10.3389/fphar.2024.1378249)
Supplement: Supplementary file 1 [file Presentation1.pdf]

Supporting Information

Supplementation of *Saussurea costus* root alleviates sodium nitrite-induced hepatorenal toxicity by modulating metabolic profile, inflammation, and apoptosis

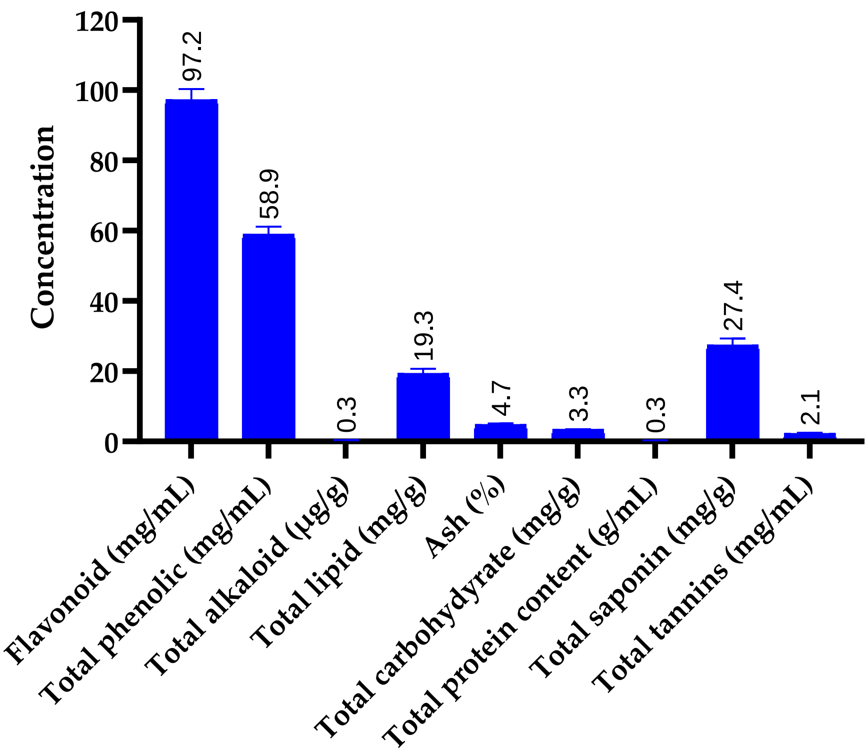

Figure S1. Phytochemical and proximate analysis of ethanolic *S. costus* root extract.

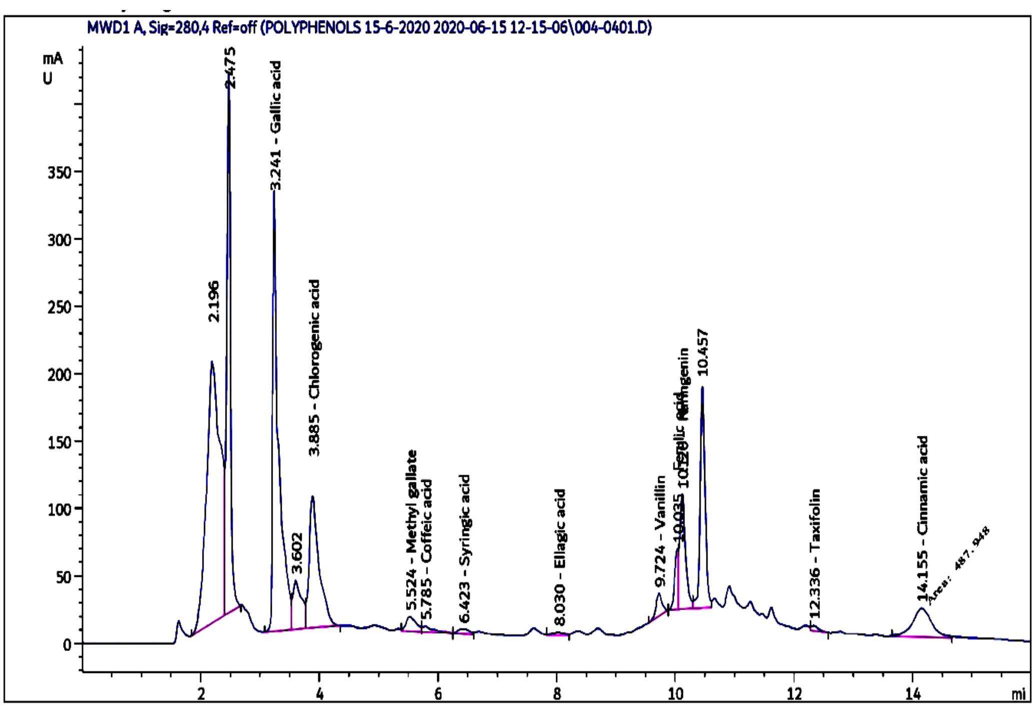

Figure S2. HPLC chromatogram of the main phenolic compounds found in the ethanolic extract of the roots of *S. costus*.

Table S1: The list of primers utilized in QRT- PCR and DD-PCR analysis

Reaction type	Primer	Sequences 5′-3′	References
QRT-PCR	IL-4(F)	CGAGTTGACCGTAACAGACAT	[1]
	IL-4(R)	CGTCTTTAGCCTTTCCAAGAAG	
	BcL-2(F)	TGTCACCACAAACAGAGACTAAAGC	[2]
	BcL-2(R)	CTTCTGCTACAAATAAGAATCGATCTC	
	P53(F)	CATCGAATTCTGGAAACTTTCCACTTGAT	[3]
	P53(R)	GTAGGAATTCGTCCCAAGCAATGGATGAT	
	TNF-α (F)	ACTGAACTTCGGGGTGATTG	[4]
	TNF-α (R)	GCTTGGTGGTTTGCTACGAC	
	β-actin (F)	TCGTGCGTGACATTAAAGAG	[5]
	β-actin (R)	ATTGCCGATAGTGATGACCT	
DD-PCR	P4	AAGAGCCCGT	[3]
	P5	AACGCGCAAC	
	G23	GTAGGCGTCG	
	C24	GGCTCGTACC	
	NAR	CTCGGCGCCATC	

Table S2: The list of metabolites detected by HPLC analysis in the SCREE.

polyphenols	Ret.Time [min]	Area	Conc.(µg/g)
Gallic acid	3.241	2262.34	7881.15
Chlorogenic acid	3.885	1143.01	3265.11
Catechin	4.313	0.00	0.00
Methyl gallate	5.524	130.58	81.74
Coffeic acid	5.785	50.96	59.77
Syringic acid	6.423	41.85	51.71
Pyro catechol	7.041	0.00	0.00
Rutin	7.275	0.00	0.00
Ellagic acid	8.030	31.61	71.85
Coumaric acid	8.932	0.00	0.00
Vanillin	9.724	119.32	115.05
Ferulic acid	10.035	173.52	194.62
Naringenin	10.120	593.41	1197.63
Taxifolin	12.336	33.94	99.03
Cinnamic acid	14.155	487.95	198.16
Kaempferol	14.657	0.00	0.00

Table S3: Effect of sodium nitrite and low, medium and high doses of *Saussurea costus* and their combination with sodium nitrite on Body weight, body weight gain (BWG), liver and kidney weight of rats.

Groups \ Parameters		Initial B.W(g)	Final B.W (g)	BWG (g)	Liver Wt.(g)	Relative liver wt.(%)	Kidney Wt.(g)	Relative kidney wt.(%)
Experimental groups	control	151 ±2.005^{a}	205 ±3.030ab	53 ±1.23ab	4.64 ±0.127ab	2.22 ±0.086cd	1.20 ±0.019ab	0.575 ±0.023a
	SC 200	149 ±2.530^{a}	210 ±6.618a	61 ±1.86a	4.45 ±0.164b	2.19±0.103cde	1.24±0.034ab	0.609±0.015a
	SC 400	154 ±2.181^{a}	211 ±5.928a	57 ±2.17ab	4.38 ±0.174b	2.07 ±0.092de	1.26 ±0.068ab	0.5297±0.043a
	SC 600	150 ±2.632^{a}	213 ±7.510a	63 ±1.71a	3.78 ±0.193c	1.85 ±0.160e	1.33 ±0.058a	0.648±0.042a
	NaNO2	149 ±2.337^{e}	175 ±4.562c	26 ±0.71c	5.13 ±0.130a	2.88 ±0.028a	0.95 ±.057d	0.534 ±0.032a
	SC 200+NaNO2	153 ±1.895^{a}	189 ±6.230bc	36±1.30bc	5.04±0.170a	2.73±0.109ab	1.01±0.035cd	0.546±0.019a
	SC400+NaNO2	153 ±2.732^{a}	196 ±5.738ab	43 ±0.63abc	4.85 ±0.115ab	2.43 ±0.070bc	1.12±0.055bcd	0.559 ±0.017a
	SC600+NaNO2	150 ±3.355^{a}	200 ±5.078ab	47 ±1.08ab	4.55 ±0.144ab	2.27 ±0.048cd	1.18±0.055abc	0.591±0.033a

Table S4: The list of detected metabolites in SCREE analysis by UPLC/T-TOF–MS/MS spectrometer in the positive ionization mode.

NO	Title	RT (min)	Precursor (*m/z*)	Area	Error (PPM)	Adduct	Reference (*m/z*)	Formula	Ontology
**1**	4-Aminophenol	0.89825	110.0082	508711	0.9	$[M+H]^+$	110.06004	C_6H_7NO	Aniline and substituted anilines
**2**	L-Arginine	0.950083 3	175.1199	205027	-0.2	$[M+H]^+$	175.11896	$C_6H_{14}N_4O_2$	L-alpha-amino acids
**3**	Zearalenone	0.950083 3	319.1552	200911	-9.1	$[M+H]^+$	319.15399	$C_{18}H_{22}O_5$	Zearalenones
**4**	S-Lactoylglutathione	0.989416 7	380.0954	821613	0.6	$[M+H]^+$	380.11221	$C_{13}H_{21}N_3O_8S$	Oligopeptides
**5**	Choline	1.028417	104.1072	3664216	-0.3	$[M]^+$	104.10645	$C_5H_{14}NO$	Cholines
**6**	3,4-Dimethoxycinnamic acid	1.04125	208.9685	57309	18.4	$[M+H]^+$	209.08084	$C_{11}H_{12}O_4$	Coumaric acids and derivatives
**7**	L-β-homotryptophan-HCl	1.09225	219.0265	2495170	0.4	$[M+H]^+$	219.11281	$C_{12}H_{14}N_2O_2$	Beta amino acids and derivatives
**8**	Xanthosine-5'-monophosphate	1.09225	365.104	564369	1.7	$[M+H]^+$	365.04929	$C_{10}H_{13}N_4O_9P$	Purine ribonucleoside monophosphates
**9**	α-Lactose	1.117733	343.1213	583581	6.6	$[M+H]^+$	343.12347	$C_{12}H_{22}O_{11}$	O-glycosyl compounds
**10**	Trigonelline	1.130233	138.053	163947	10	$[M+H]^+$	138.05496	$C_7H_7NO_2$	Alkaloids and derivatives
**11**	L- Proline	1.143233	116.0685	6683844	14.6	$[M+H]^+$	116.0706	$C_5H_9NO_2$	Proline and derivatives
**12**	3-Chloro-L-tyrosine	1.155917	216.1162	39515	26.4	$[M+H]^+$	216.04219	$C_9H_{10}ClNO_3$	Tyrosine and derivatives
**13**	Adenine	1.1944	136.0628	106874	-3.7	$[M+H]^+$	136.06177	$C_5H_5N_5$	6-aminopurines
**14**	Guanosine	1.220067	284.103	30855	2.2	$[M+H]^+$	284.09894	$C_{10}H_{13}N_5O_5$	Purine nucleosides
**15**	L-β-Homothreonine	1.2599	134.0443	76501	-0.7	$[M+H]^+$	134.08118	$C_5H_{11}NO_3$	Beta amino acids and derivatives
**16**	β-Nicotinamide mononucleotide	1.349733	335.0423	227914	1.6	$[M+H]^+$	335.06387	$C_{11}H_{15}N_2O_8P$	Nicotinamide nucleotides

**17**	D-Alloisoleucine	1.388567	132.1009	134614	1.1	$[M+H]^+$	132.1019	$C_6H_{13}NO_2$	Isoleucine and derivatives
**18**	Nicotinic acid	1.427883	124.0359	23662	20.7	$[M+H]^+$	124.03931	$C_6H_5NO_2$	Pyridinecarboxylic acids
**19**	Adenosine 3':5'-cyclicmonophosphate	1.532717	330.0566	1103257	6.5	$[M+H]^+$	330.05978	$C_{10}H_{12}N_5O_6P$	3',5'-cyclic purine nucleotides
**20**	Nicotinamide	1.558717	123.0536	14361	12.6	$[M+H]^+$	123.05529	$C_6H_6N_2O$	Nicotinamides
**21**	L-5-Oxoproline	1.611217	130.0489	882512	1.8	$[M+H]^+$	130.04987	$C_5H_7NO_3$	Alpha amino acids and derivatives
**22**	Piperidine	1.690383	86.06098	96568	-8.6	$[M+H]^+$	86.09643	$C_5H_{11}N$	Piperidines
**23**	Mannose 6-phosphate	1.73005	261.034	46460	9.5	$[M+H]^+$	261.03699	$C_6H_{13}O_9P$	Hexose phosphates
**24**	3-Methyladenine	1.760767	150.0923	67190	-6.4	$[M+H]^+$	150.07742	$C_6H_7N_5$	6-aminopurines
**25**	L-β-Homoleucine	1.910533	146.0899	37547	11.3	$[M+H]^+$	146.11755	$C_7H_{15}NO_2$	Beta amino acids and derivatives
**26**	Glycine-Betaine	1.961033	118.0871	148059	-1.8	$[M+H]^+$	118.08626	$C_5H_{11}NO_2$	Alpha amino acids
**27**	Pipecolate	2.094017	130.0845	100975	14	$[M+H]^+$	130.08626	$C_6H_{11}NO_2$	Alpha amino acids
**28**	L-(-)-Phenylalanine	2.14685	166.0845	253323	7.7	$[M+H]^+$	166.08626	$C_9H_{11}NO_2$	Phenylalanine and derivatives
**29**	*N*-Acetylneuraminate	2.159517	310.1273	435258	2.2	$[M+H]^+$	310.11325	$C_{11}H_{19}NO_9$	N-acylneuraminic acids
**30**	Histidinol	2.536333	142.0827	36665	18.2	$[M+H]^+$	142.09749	$C_6H_{11}N_3O$	Aralkylamines
**31**	*p*-Aminobenzoic acid	4.05375	138.0549	28759	-1.1	$[M+H]^+$	138.05496	$C_7H_7NO_2$	Aminobenzoic acids
**32**	Naringenin	4.394233	273.0913	102012	-11.2	$[M+H]^+$	273.07574	$C_{15}H_{12}O_5$	Flavanones
**33**	Histidine	4.532733	156.1007	33485	3.4	$[M+H]^+$	156.07675	$C_6H_9N_3O_2$	Histidine and derivatives
**34**	Cinnamaldehyde	4.700217	133.0635	99765	0.2	$[M+H]^+$	133.06479	C_9H_8O	Cinnamaldehydes
**35**	Phlorizin	4.713383	437.1581	53916	-0.7	$[M+H]^+$	437.14423	$C_{21}H_{24}O_{10}$	Flavonoid O-glycosides
**36**	4-Methyl-5-thiazoleethanol	4.87905	144.0792	48710	0.7	$[M+H]^+$	144.04776	C_6H_9NOS	4,5-disubstituted thiazoles
**37**	Uric acid	4.87905	169.0809	15425	-19.7	$[M+H]^+$	169.03561	$C_5H_4N_4O_3$	Xanthines

**38**	Chlorogenic acid	5.048367	355.0869	630878	43.1	$[M+H]^+$	355.10236	$C_{16}H_{18}O_9$	Quinic acids and derivatives
**39**	(-)-Riboflavin	5.0737	377.0838	132512	0.7	$[M+H]^+$	377.14557	$C_{17}H_{20}N_4O_6$	Flavins
**40**	*o*-Aminobenzoic acid	5.1647	138.0533	31474	8.1	$[M+H]^+$	138.05496	$C_7H_7NO_2$	Aminobenzoic acids
**41**	Procyanidin B2	5.217367	579.1418	15896	11.2	$[M+H]^+$	579.14972	$C_{30}H_{26}O_{12}$	Biflavonoids and polyflavonoids
**42**	4-Hydroxy-3-methoxybenzaldehyde	5.54135	153.0827	12431	48.9	$[M+H]^+$	153.05463	$C_8H_8O_3$	Methoxyphenols
**43**	Catechin	5.658667	291.0861	28714	-0.6	$[M+H]^+$	291.0863	$C_{15}H_{14}O_6$	Catechins
**44**	Cholic acid	5.6715	409.1872	54437	-2	$[M+H]^+$	409.29486	$C_{24}H_{40}O_5$	Trihydroxy bile acids, alcohols and derivatives
**45**	L-Tryptophan	5.903316	205.0833	89806	12.1	$[M+H]^+$	205.09715	$C_{11}H_{12}N_2O_2$	Indolyl carboxylic acids and derivatives
**46**	3,3-D2-flavanone	5.94265	225.0716	97015	4	$[M+H]^+$	225.091	$C_{15}H_{12}O_2$	Flavanones
**47**	3-Hydroxyanthranilic acid	6.22565	154.0853	24204	0.7	$[M+H]^+$	154.04987	$C_7H_7NO_3$	Hydroxybenzoic acid derivatives
**48**	5-Hydroxyindoleacetic acid	6.251633	192.1021	9327	-6.1	$[M+H]^+$	192.06552	$C_{10}H_9NO_3$	Indole-3-acetic acid derivatives
**49**	3-(4-Hydroxy-3-methoxyphenyl)prop-2-enoic acid	6.332133	195.1122	25586	-54.1	$[M+H]^+$	195.06519	$C_{10}H_{10}O_4$	Hydroxycinnamic acids
**50**	trans-Zeatin riboside	6.373633	352.2427	53148	13	$[M+H]^+$	352.16156	$C_{15}H_{21}N_5O_5$	Purine nucleosides
**51**	Maritimetin-6-O-glucoside	6.647617	449.18	23565	-1	$[M+H]^+$	449.10785	$C_{21}H_{20}O_{11}$	Aurone O-glycosides
**52**	Pelargonidin-3,5-di-O-glucoside	6.647617	595.1999	14227	0.9	$[M]^+$	595.16522	$C_{27}H_{31}O_{15}$	Anthocyanidin-5-O-glycosides
**53**	2'-Deoxyadenosine 5'-monophosphate	6.9431	332.1848	39064	0.7	$[M+H]^+$	332.07544	$C_{10}H_{14}N_5O_6P$	Purine 2'-deoxyribonucleoside monophosphates
**54**	1H-Indole-3-carboxylic acid	7.050267	162.0892	127206	7.8	$[M+H]^+$	162.05496	$C_9H_7NO_2$	Indolecarboxylic acids and derivatives

**55**	Caffeic acid	7.050267	181.1096	63259	-22.6	$[M+H]^+$	181.04953	$C_9H_8O_4$	Hydroxycinnamic acids
**56**	Syringaldehyde	7.117767	183.0663	162580	-5.9	$[M+H]^+$	183.06519	$C_9H_{10}O_4$	Methoxyphenols
**57**	Cyanidin-3-glucoside	7.17075	449.1052	226538	4.2	$[M]^+$	449.1073	$C_{21}H_{21}O_{11}$	Anthocyanidin-3-O-glycosides
**58**	Amantadine	7.35625	152.0678	42890	13.7	$[M+H]^+$	152.14337	$C_{10}H_{17}N$	Monoalkylamines
**59**	3-Methylxanthine	7.419417	167.0692	26190	2	$[M+H]^+$	167.05635	$C_6H_6N_4O_2$	Xanthines
**60**	*o*-Methoxy benzoic acids	7.748233	153.1263	20199	4.6	$[M+H]^+$	153.05463	$C_8H_8O_3$	O-methoxybenzoic acids and derivatives
**61**	apigenin-7-*O*-glucoside	7.912384	433.1081	660019	8.9	$[M+H]^+$	433.11292	$C_{21}H_{20}O_{10}$	Flavonoid-7-O-glycosides
**62**	Procyanidin B1	7.964716	579.1388	11483	14.4	$[M+H]^+$	579.14972	$C_{30}H_{26}O_{12}$	Biflavonoids and polyflavonoids
**63**	Peonidine-3-*O*-glucoside chloride	8.112866	463.1234	30276	0.5	$[M]^+$	463.12292	$C_{22}H_{23}O_{11}$	Anthocyanidin-3-O-glycosides
**64**	12-Oxo-10,15(*Z*)-Phytodienoic Acid	8.139533	293.1788	31904	4.3	$[M+H]^+$	293.21112	$C_{18}H_{28}O_3$	Prostaglandins and related compounds
**65**	3-Formylindol	8.1927	146.0609	83882	-3.9	$[M+H]^+$	146.06004	C_9H_7NO	Indoles
**66**	Okanin-4'-*O*-glucoside	8.271533	451.1935	116722	0.1	$[M+H]^+$	451.12347	$C_{21}H_{22}O_{11}$	Flavonoid O-glycosides
**67**	Sinapyl aldehyde	8.393867	209.0828	190119	-6.4	$[M+H]^+$	209.08084	$C_{11}H_{12}O_4$	Methoxyphenols
**68**	4-Hydroxy-3-methoxycinnamaldehyde	8.420016	179.0705	42010	0.9	$[M+H]^+$	179.07027	$C_{10}H_{10}O_3$	Methoxyphenols
**69**	Genistein	8.47285	271.0577	76090	4.6	$[M+H]^+$	271.06009	$C_{15}H_{10}O_5$	Isoflavones
**70**	Riboflavin-5-monophosphate sodium salt	8.52435	457.1466	17752	0.8	$[M+H]^+$	457.11188	$C_{17}H_{21}N_4O_9P$	Flavin nucleotides
**71**	D-(+)-Glucosamine hydrochloride	8.620017	180.0826	48467	17.9	$[M+H]^+$	180.08665	$C_6H_{13}NO_5$	Hexoses
**72**	Sissotrin	9.034333	447.1415	66292	0.2	$[M+H]^+$	447.12857	$C_{22}H_{22}O_{10}$	Isoflavonoid O-glycosides
**73**	D-2-Aminoadipic acid	9.166483	162.0908	83588	0.8	$[M+H]^+$	162.07608	$C_6H_{11}NO_4$	L-alpha-amino acids

**74**	S-Adenosyl-L-homocysteine	9.303817	385.1696	139852	7.7	$[M+H]^+$	385.12888	$C_{14}H_{20}N_6O_5S$	5'-deoxy-5'-thionucleosides
**75**	Acacetin-7-*O*-rutinoside	9.303817	593.1765	9937	11.3	$[M+H]^+$	593.18646	$C_{28}H_{32}O_{14}$	Flavonoid-7-O-glycosides
**76**	Scopoletin	9.316816	193.0485	610938	1.3	$[M+H]^+$	193.04953	$C_{10}H_8O_4$	7-hydroxycoumarins
**77**	Quercetin-4'-glucoside	9.463966	465.1342	33475	-0.6	$[M+H]^+$	465.10275	$C_{21}H_{20}O_{12}$	Flavonoid O-glycosides
**78**	Naringenin-7-*O*-glucoside	9.735467	435.1888	24905	-1.6	$[M+H]^+$	435.12857	$C_{21}H_{22}O_{10}$	Flavonoid-7-O-glycosides
**79**	Ononin	9.816116	431.1292	177465	5	$[M+H]^+$	431.13367	$C_{22}H_{22}O_9$	Isoflavonoid O-glycosides
**80**	Apigenin	9.929116	271.0583	788675	2.9	$[M+H]^+$	271.06009	$C_{15}H_{10}O_5$	Flavones
**81**	Luteolin	9.941783	287.0626	122277	-16.5	$[M+H]^+$	287.05502	$C_{15}H_{10}O_6$	Flavones
**82**	4'-Hydroxyisoflavone-7-*O*-glucoside	10.07893	417.1099	19885	8.5	$[M+H]^+$	417.11801	$C_{21}H_{20}O_9$	Isoflavonoid O-glycosides
**83**	3' 4' 5 7-Tetrahydroxyflavanone	10.92645	289.1409	1126044	0.5	$[M+H]^+$	289.07068	$C_{15}H_{12}O_6$	Flavanones
**84**	Acacetin	11.05405	285.0754	1473894	1	$[M+H]^+$	285.07574	$C_{16}H_{12}O_5$	4'-O-methylated flavonoids
**85**	Baicalein-7-*O*-glucuronide	11.79602	447.1595	205857	3.8	$[M+H]^+$	447.09219	$C_{21}H_{18}O_{11}$	Flavonoid-7-O-glucuronides
**86**	Formononetin	12.21233	269.1156	415852	-0.8	$[M+H]^+$	269.08084	$C_{16}H_{12}O_4$	4'-O-methylisoflavones
**87**	Gossypin	12.25098	481.1096	13677	-0.3	$[M+H]^+$	481.09766	$C_{21}H_{20}O_{13}$	Flavonoid-8-O-glycosides
**88**	S-Adenosyl-L-methionine	12.89928	399.1802	182474	-0.9	$[M+H]^+$	399.1445	$C_{15}H_{22}N_6O_5S$	5'-deoxy-5'-thionucleosides
**89**	Calciferol	13.15295	397.1597	395931	6.8	$[M+H]^+$	397.3465	$C_{28}H_{44}O$	Vitamin D and derivatives
**90**	1,3-Dimethylurate	13.44827	197.128	18407	6.1	$[M+H]^+$	197.06691	$C_7H_8N_4O_3$	Xanthines

**91**	α-D-glucose-1-phosphate dipotassium salt dihydate	14.35725	261.1778	345933	14.7	$[M+H]^+$	261.03699	$C_6H_{13}O_9P$	Monosaccharide phosphates
**92**	Scoulerin	14.6057	328.1895	454629	5	$[M+H]^+$	328.15433	$C_{19}H_{21}NO_4$	Protoberberine alkaloids and derivatives
**93**	DL-α,epsilon-Diaminopimelic acid	14.94135	191.1423	74230	1.3	$[M+H]^+$	191.10263	$C_7H_{14}N_2O_4$	Alpha amino acids
**94**	Daidzein	16.87375	255.1357	3149348	0.3	$[M+H]^+$	255.06519	$C_{15}H_{10}O_4$	Isoflavones
**95**	Urocanic acid	16.87375	139.0725	264946	13.3	$[M+H]^+$	139.0502	$C_6H_6N_2O_2$	Imidazolyl carboxylic acids and derivatives
**96**	3-(4-hydroxyphenyl)prop-2-enoic acid	16.88658	165.0909	263389	-125.2	$[M+H]^+$	165.05463	$C_9H_8O_3$	Hydroxycinnamic acids
**97**	Isoguvacine	16.91208	128.0603	185598	10.9	$[M+H]^+$	128.0706	$C_6H_9NO_2$	Hydropyridines
**98**	Hinokitiol	17.25157	165.0693	290066	0.7	$[M+H]^+$	165.091	$C_{10}H_{12}O_2$	Tropolones
**99**	Pyridoxamine	17.26423	169.0997	197287	3	$[M+H]^+$	169.09715	$C_8H_{12}N_2O_2$	Pyridoxamine 5'-phosphates
**100**	all-*trans*-Retinoic acid	18.645	301.225	19747	-18.7	$[M+H]^+$	301.21622	$C_{20}H_{28}O_2$	Retinoids
**101**	6,7-DIHYDROXYCOUMARIN	18.72048	179.1426	77536	0.4	$[M+H]^+$	179.03389	$C_9H_6O_4$	6,7-dihydroxycoumarins
**102**	3 5 7-Trihydroxy-4'-methoxyflavone	19.68043	301.1411	2547620	0.6	$[M+H]^+$	301.07068	$C_{16}H_{12}O_6$	Flavonols
**103**	2'-Deoxycytidine	19.90458	228.2313	726579	2.9	$[M+H]^+$	228.09789	$C_9H_{13}N_3O_4$	Pyrimidine 2'-deoxyribonucleosides
**104**	Leupeptin hemisulfate salt	21.7125	427.2616	44926	1.7	$[M+H]^+$	427.30273	$C_{20}H_{38}N_6O_4$	Dipeptides

Table S5: The list of detected metabolites in SCREE analysis by UPLC/T-TOF–MS/MS spectrometer in the negative ionization mode.

NO	Title	RT (min)	Precursor (*m/z*)	Area	Error (PPM)	Adduct	Reference (*m/z*)	Formula	Ontology
**1**	Quercetin	0.8016334	300.9552	7955	-5.6	$[M-H]^-$	301.03537	$C_{15}H_{10}O_7$	Flavonols
**2**	D-(+)-Malic acid	0.8788	133.0137	5064285	-0.8	$[M-H]^-$	133.01425	$C_4H_6O_5$	Beta hydroxy acids and derivatives
**3**	Maleic acid	0.8788	115.0028	975943	0.6	$[M-H]^-$	115.00368	$C_4H_4O_4$	Dicarboxylic acids and derivatives
**4**	Succinic acid	0.8918	117.0198	167201	-7.4	$[M-H]^-$	117.01933	$C_4H_6O_4$	Dicarboxylic acids and derivatives
**5**	Mannose 6-phosphate	0.8918	259.0194	115209	4.7	$[M-H]^-$	259.02243	$C_6H_{13}O_9P$	Hexose phosphates
**6**	DL-γ-Hydroxybutyric acid	0.9173	103.0052	48571	-14.4	$[M-H]^-$	103.04007	$C_4H_8O_3$	Beta hydroxy acids and derivatives
**7**	Gluconate	0.9553	195.05	2141325	3.1	$[M-H]^-$	195.05103	$C_6H_{12}O_7$	hydroxy acids and derivatives
**8**	Citrate	0.9553	191.0549	1712133	2.6	$[M-H]^-$	191.01973	$C_6H_8O_7$	Tricarboxylic acids and derivatives
**9**	L-Glutamic acid	0.9553	146.0452	73018	-0.2	$[M-H]^-$	146.04588	$C_5H_9NO_4$	Glutamic acid and derivatives
**10**	Lactic acid	0.9683	89.02406	141830	-0.3	$[M-H]^-$	89.02441	$C_3H_6O_3$	Alpha hydroxy acids and derivatives
**11**	Glyceric acid	0.9683	105.0187	115839	0.4	$[M-H]^-$	105.01933	$C_3H_6O_4$	Sugar acids and derivatives
**12**	L-(+)-Tartrate	0.9683	149.0437	40005	2.7	$[M-H]^-$	149.00916	$C_4H_6O_6$	Sugar acids and derivatives
**13**	L-5-Oxoproline	0.9808	128.0347	704771	0	$[M-H]^-$	128.03532	$C_5H_7NO_3$	Alpha amino acids and derivatives
**14**	L-β-Homolysine	1.019617	159.0279	20945	5.2	$[M-H]^-$	159.11391	$C_7H_{16}N_2O_2$	Beta amino acids and derivatives

**15**	Baicalein-7-*O*-glucuronide	1.045133	445.1202	29479	-0.1	$[M-H]^-$	445.07764	$C_{21}H_{18}O_{11}$	Flavonoid-7-O-glucuronides
**16**	Hesperetin-7-*O*-neohesperidoside	1.058283	609.1917	60901	-1	$[M-H]^-$	609.1825	$C_{28}H_{34}O_{15}$	Flavonoid-7-O-glycosides
**17**	Sucrose	1.10895	341.1085	8329249	0.7	$[M-H]^-$	341.10895	$C_{12}H_{22}O_{11}$	O-glycosyl compounds
**18**	D-(+)-Raffinose	1.10895	503.1643	990184	-3.9	$[M-H]^-$	503.16177	$C_{18}H_{32}O_{16}$	Oligosaccharides
**19**	Galactinol Dihydrate	1.147283	341.0901	415084	53.7	$[M-H]^-$	341.10895	$C_{12}H_{22}O_{11}$	O-glycosyl compounds
**20**	Dihydroorotate	1.19845	157.0503	29495	-0.7	$[M-H]^-$	157.02548	$C_5H_6N_2O_4$	Alpha amino acids and derivatives
**21**	Chlorogenic acid	1.211617	353.0851	1833928	6.4	$[M-H]^-$	353.0878	$C_{16}H_{18}O_9$	Quinic acids and derivatives
**22**	Uridine	1.211617	243.0607	137955	1.7	$[M-H]^-$	243.06226	$C_9H_{12}N_2O_6$	Pyrimidine nucleosides
**23**	γ-Terpinene	1.211617	135.0427	40432	-98	$[M-H]^-$	135.11792	$C_{10}H_{16}$	Branched unsaturated hydrocarbons
**24**	2,5-Di Hydroxybenzoic acid	1.211617	153.0165	32037	6.2	$[M-H]^-$	153.01933	$C_7H_6O_4$	Hydroxybenzoic acid derivatives
**25**	trans-4-Hydroxy-L-proline	1.3661	130.0836	94671	16.5	$[M-H]^-$	130.05096	$C_5H_9NO_3$	Proline and derivatives
**26**	Adenine	1.4551	134.0448	104149	10	$[M-H]^-$	134.04723	$C_5H_5N_5$	6-aminopurines
**27**	Isonicotinate	1.843583	122.0218	226429	15.4	$[M-H]^-$	122.02475	$C_6H_5NO_2$	Pyridinecarboxylic acids
**28**	L-(-)-Phenylalanine	2.003567	164.0716	78690	-0.6	$[M-H]^-$	164.0717	$C_9H_{11}NO_2$	Phenylalanine and derivatives
**29**	*P*- Hydroxybenzoic acid	2.450317	137.024	92476	-0.9	$[M-H]^-$	137.02441	$C_7H_6O_3$	Hydroxybenzoic acid derivatives

**30**	3,4-Di Hydroxybenzoic acid	4.188617	153.0146	19647	19.9	$[M-H]^-$	153.01933	$C_7H_6O_4$	Hydroxybenzoic acid derivatives
**31**	Caffeic acidD	4.239617	179.0349	134397	-0.1	$[M-H]^-$	179.03499	$C_9H_8O_4$	Hydroxycinnamic acids
**32**	D-(-)-Quinic acid	4.252283	191.0591	142582	-14.6	$[M-H]^-$	191.05611	$C_7H_{12}O_6$	Quinic acids and derivatives
**33**	Acacetin	4.539117	283.0686	30943	1.8	$[M-H]^-$	283.06119	$C_{16}H_{12}O_5$	4'-O-methylated flavonoids
**34**	Isorhamnetin-3-O-rutinoside	4.933084	623.126	45928	-0.7	$[M-H]^-$	623.16174	$C_{28}H_{32}O_{16}$	Flavonoid-3-O-glycosides
**35**	1,2-Benzenediol	4.933084	109.0294	15524	0.1	$[M-H]^-$	109.0295	$C_6H_6O_2$	Catechols
**36**	3'-methoxy-4',5,7-trihydroxyflavonol	5.075917	315.1057	79765	4.2	$[M-H]^-$	315.05103	$C_{16}H_{12}O_7$	Flavonols
**37**	DL-5-Hydroxylysine	5.195117	161.022	358425	10.2	$[M-H]^-$	161.09317	$C_6H_{14}N_2O_3$	Alpha amino acids
**38**	Suberic acid	5.435233	173.0498	49810	-16.9	$[M-H]^-$	173.08194	$C_8H_{14}O_4$	Medium-chain fatty acids
**39**	Glycyl-L-proline	5.632216	171.1006	35925	3.8	$[M-H]^-$	171.07751	$C_7H_{12}N_2O_3$	Dipeptides
**40**	Gibberelin A3	6.098017	345.1001	239778	2.2	$[M-H]^-$	345.13437	$C_{19}H_{22}O_6$	C19-gibberellin 6-carboxylic acids
**41**	*O*- Aminobenzoic acid	6.22535	136.0148	28890	6.5	$[M-H]^-$	136.04041	$C_7H_7NO_2$	Aminobenzoic acids
**42**	Carnosine	6.480834	225.055	13344	0.6	$[M-H]^-$	225.09932	$C_9H_{14}N_4O_3$	Hybrid peptides
**43**	Syringaldehyde	6.5195	181.049	136585	4	$[M-H]^-$	181.05063	$C_9H_{10}O_4$	Methoxyphenols
**44**	Hinokitiol	6.545	163.0767	84981	0.1	$[M-H]^-$	163.07645	$C_{10}H_{12}O_2$	Tropolones
**45**	Maritimetin-6-*O*-glucoside	7.025983	447.0921	577112	0.4	$[M-H]^-$	447.09329	$C_{21}H_{20}O_{11}$	Aurone O-glycosides
**46**	Datiscin	7.025983	593.1527	40519	-0.6	$[M-H]^-$	593.15118	$C_{27}H_{30}O_{15}$	Flavonoid-3-O-glycosides
**47**	β- Indoleacetic acid	7.076633	174.0582	63351	-11.1	$[M-H]^-$	174.05605	$C_{10}H_9NO_2$	Indole-3-acetic acid derivatives

**48**	Eriodictyol-7-O-glucoside	7.089633	449.1786	27021	4.8	[M-H]$^-$	449.10895	$C_{21}H_{22}O_{11}$	Flavonoid-7-O-glycosides
**49**	Inosine	7.255967	267.16	136110	0.4	[M-H]$^-$	267.07349	$C1_{0}H_{12}N_{4}O_{5}$	Purine nucleosides
**50**	3 5 7-Trihydroxy-4'-methoxyflavone	7.541117	299.0556	445854	0.8	[M-H]$^-$	299.05612	$C_{16}H_{12}O_{6}$	Flavonols
**51**	Daidzein-8-C-glucoside	7.554117	415.1964	190308	0.5	[M-H]$^-$	415.10345	$C_{21}H_{20}O_{9}$	Isoflavonoid C-glycosides
**52**	Gibberellin A4	7.579617	331.1766	20286	-0.4	[M-H]$^-$	331.15509	$C_{19}H_{24}O_{5}$	C19-gibberellin 6-carboxylic acids
**53**	Apigenin-7-O-glucoside	7.702683	431.101	3585188	-4.3	[M-H]$^-$	431.09836	$C_{21}H_{20}O_{10}$	Flavonoid-7-O-glycosides
**54**	2'-Deoxyinosine	8.011583	251.1655	806060	-0.8	[M-H]$^-$	251.07858	$C_{10}H_{12}N_{4}O_{4}$	Purine 2'-deoxyribonucleosides
**55**	L-β-Homoisoleucine	8.05025	144.0453	239559	-0.9	[M-H]$^-$	144.103	$C_{7}H_{15}NO_{2}$	Beta amino acids and derivatives
**56**	Pelargonidin-3-O-glucoside	8.100917	431.0977	3749567	0.7	[M-2H]$^-$	431.09781	$C_{21}H_{21}O_{10}$	Anthocyanidin-3-O-glycosides
**57**	Sinapyl aldehyde	8.177417	207.0677	131933	-6	[M-H]$^-$	207.06628	$C_{11}H_{12}O_{4}$	Methoxyphenols
**58**	4-Hydroxy-3-methoxycinnamaldehyde	8.177417	177.0558	29038	-0.2	[M-H]$^-$	177.05573	$C_{10}H_{10}O_{3}$	Methoxyphenols
**59**	Naringenin-7-O-glucoside	8.31725	433.1527	84432	-88.2	[M-H]$^-$	433.11401	$C_{21}H_{22}O_{10}$	Flavonoid-7-O-glycosides
**60**	Thymidine-5'-monophosphate	8.31725	321.1274	21865	19.2	[M-H]$^-$	321.04932	$C_{10}H_{15}N_{2}O_{8}P$	Pyrimidine 2'-deoxyribonucleoside monophosphates
**61**	Isocitrate	8.508067	191.035	2382439	-0.2	[M-H]$^-$	191.01973	$C_{6}H_{8}O_{7}$	Tricarboxylic acids and derivatives
**62**	3-Methylxanthine	8.5204	165.0554	88020	-0.2	[M-H]$^-$	165.04179	$C_{6}H_{6}N_{4}O_{2}$	Xanthines
**63**	Luteolin	8.860717	285.0393	1899609	3.4	[M-H]$^-$	285.04047	$C_{15}H_{10}O_{6}$	Flavones

**64**	3' 4' 5 7-Tetrahydroxyflavanone	8.962367	287.0428	57037	10.1	$[M-H]^-$	287.05612	$C_{15}H_{12}O_6$	Flavanones
**65**	D-(+)-Galacturonic acid	9.038533	193.0494	125570	0.9	$[M-H]^-$	193.03537	$C_6H_{10}O_7$	Glucuronic acid derivatives
**66**	Riboflavin-5?-monophosphate sodium salt hydrate	9.189366	455.1911	1742561	1.1	$[M-H]^-$	455.09735	$C_{17}H_{21}N_4O_9P$	Flavin nucleotides
**67**	Scopoletin	9.426683	191.0359	181842	-82.2	$[M-H]^-$	191.03499	$C_{10}H_8O_4$	7-hydroxycoumarins
**68**	Sodium Deoxycholate	9.502183	391.1435	627399	-7.3	$[M-H]^-$	391.28537	$C_{24}H_{40}O_4$	Dihydroxy bile acids, alcohols and derivatives
**69**	Anserine	9.740334	239.0679	30147	0.9	$[M-H]^-$	239.11496	$C_{10}H_{16}N_4O_3$	Hybrid peptides
**70**	Naringenin	9.9405	271.0567	56123	0.6	$[M-H]^-$	271.06119	$C_{15}H_{12}O_5$	Flavanones
**71**	Luteolin-7-O-glucoside	9.990483	447.0917	61039	1.3	$[M-H]^-$	447.09329	$C_{21}H_{20}O_{11}$	Flavonoid-7-O-glycosides
**72**	2'-Deoxyinosine 5'-monophosphate	10.06582	330.9789	152908	0.9	$[M-H]^-$	331.04492	$C_{10}H_{13}N_4O_7P$	Purine 2'-deoxyribonucleoside monophosphates
**73**	Cytidine-5'-monophosphate	11.20675	322.1512	30314	8.7	$[M-H]^-$	322.04459	$C_9H_{14}N_3O_8P$	Pyrimidine ribonucleoside monophosphates
**74**	L-(+)-Rhamnose	12.67718	163.0764	63628	0.5	$[M-H]^-$	163.0612	$C_6H_{12}O_5$	Hexoses
**75**	*E*-4,5'-dihydroxy-3-methoxy-3'-glucopyranosylstilbene	12.89367	419.1707	1243786	0.1	$[M-H]^-$	419.13477	$C_{21}H_{24}O_9$	Stilbene glycosides
**76**	Apigenin	13.38215	269.0452	38379868	0.8	$[M-H]^-$	269.04553	$C_{15}H_{10}O_5$	Flavones
**77**	Esculin	15.78452	339.199	272306	0.7	$[M-H]^-$	339.07214	$C_{15}H_{16}O_9$	Coumarin glycosides

**78**	γ , γ -Dimethyallyl pyrophosphate ammonium salt	17.16195	245.1184	606731	-0.4	$[M-H]^-$	244.99855	$C_5H_{12}O_7P_2$	Isoprenoid phosphates
**79**	Cytidine-3',5'-cyclicmonophosphate	17.18728	304.1194	97099	-0.7	$[M-H]^-$	304.034	$C_9H_{12}N_3O_7P$	Pentose phosphates
**80**	Inosine-5'-monophosphate	17.6646	347.1306	1385509	-4.6	$[M-H]^-$	347.03983	$C_{10}H_{13}N_4O_8P$	Purine ribonucleoside monophosphates
**81**	Daidzein	18.30638	253.0493	904209	2.3	$[M-H]^-$	253.05063	$C_{15}H_{10}O_4$	Isoflavones
**82**	γ-Linolenic acid	19.07068	277.2178	15435236	-0.7	$[M-H]^-$	277.21732	$C_{18}H_{30}O_2$	Lineolic acids and derivatives
**83**	Etidronate	19.63115	205.1561	15597	15.1	$[M-H]^-$	204.96725	$C_2H_8O_7P_2$	Bisphosphonates

References

1. Makled, S. O.; Hamdan, A. M.; El-Sayed, A.-F. M.; Hafez, E. E., Evaluation of marine psychrophile, Psychrobacter namhaensis SO89, as a probiotic in Nile tilapia (Oreochromis niloticus) diets. *Fish & shellfish immunology* **2017,** 61, 194-200.
2. Yamagata, K.; Izawa, Y.; Onodera, D.; Tagami, M., Chlorogenic acid regulates apoptosis and stem cell marker-related gene expression in A549 human lung cancer cells. *Molecular and Cellular Biochemistry* **2018,** 441, (1), 9-19.
3. Hamad, G.; Hafez, E., Amino Acids Diets as Model for Investigating Cancer Induced by Acrylamide Produced during Wrong Food Cooking. *SOJ Biochem* **2018,** 4, (1), 1-14.
4. Khan, M. A.; Alam, A.; Husain, S.; Ahmed, S.; Nazamuddin, M.; Ahmed, Z. Q., a potent herb of Unani medicine: A review. *Int J Curr Pharm Res* **2013,** 5, (4), 1-4.
5. Habotta, O. A.; Ateya, A.; Saleh, R. M.; El-Ashry, E. S., Thiamethoxam-induced oxidative stress, lipid peroxidation, and disturbance of steroidogenic genes in male rats: Palliative role of Saussurea lappa and Silybum marianum. *Environmental Toxicology* **2021,** 36, (10), 2051-2061.
